# Biocontrol of Postharvest Soft Rot in Kiwifruit by Antagonistic Fungi from Kiwifruit Tissues and Rhizosphere Soil: Screening, Identification, and Mechanisms

**DOI:** 10.3390/jof12080600

**Published:** 2026-08-12

**Authors:** Jiqing Lei, Hong Li, Yinna Shi, Lulu Yang, Jinyong Deng, Ning Ji, Rui Wang

**Affiliations:** 1 Department of Food Science and Engineering, Guiyang University, Guiyang 550005, China; jiqingleigyu@163.com (J.L.); lihong@gyu.edu.cn (H.L.); syn1354890492@163.com (Y.S.); yanglulu@gyu.edu.cn (L.Y.); dengjinuong@gyu.edu.cn (J.D.); jining552100@163.com (N.J.); 2 Guizhou Key Laboratory of New Quality Processing and Storage of Ecological Specialty Food, Guiyang University, Guiyang 550005, China

**Keywords:** kiwifruit, soft rot, antagonistic fungi, biocontronl

## Abstract

Postharvest soft rot is a major constraint in kiwifruit production, causing substantial economic losses and raising food safety concerns. Therefore, the development of sustainable biocontrol alternatives to conventional chemical treatments is urgently needed. In this study, thirty-seven fungi were isolated from healthy kiwifruit tissues and rhizosphere soil and screened for their biocontrol potential. Three highly effective antagonistic strains were ultimately identified: *Mucor circinelloides* N2-1-1, *Aspergillus niger* Pb-2-2, and *Trichoderma hamatum* Nd-1-1. In vitro antagonism assays showed that *A. niger* Pb-2-2 exhibited the strongest and broadest-spectrum inhibitory activity against four postharvest pathogens, namely *Fusarium* sp., *Alternaria* sp., *Botryosphaeriaceae* sp., and *Phomopsis* sp. In contrast, in vivo protection assays demonstrated that *T. hamatum* Nd-1-1 almost provided the highest control efficacy against all four pathogens in kiwifruit fruit. Fermentation broth protection experiments further revealed that *A. niger* Pb-2-2 produced stable and broad-spectrum antimicrobial metabolites. Although *M. circinelloides* N2-1-1 showed the weakest antagonistic activity among the three fungi, this is the first report indicating that *M. circinelloides* has antagonistic activity against phytopathogenic fungi. Physiological analyses indicated that all three antagonistic fungi alleviated the inhibitory effects of pathogens on superoxide dismutase, catalase, and ascorbate peroxidase activities, helped maintain reactive oxygen species homeostasis, and activated the phenylpropanoid pathway, thereby enhancing disease resistance in kiwifruit. These findings provide a theoretical basis and promising microbial resources for the biological control of postharvest soft rot in kiwifruit.

## 1. Introduction

Kiwifruit (*Actinidia chinensis*), a prominent species of the Actinidiaceae family within the order Ericales, is not only native to China but has also emerged as a globally economically important crop, celebrated for its superior nutritional profile and proven benefits for digestive, immune, and metabolic health [1,2]. However, the sustainable expansion of the kiwifruit industry is currently facing a critical bottleneck of fungal soft rot. This disease has evolved into a persistent and devastating epidemic across China’s major production regions, with incidence rates escalating to 40% to 50% [3]. As a primary limiting factor, soft rot causes precipitous economic losses and threatens the long-term viability of the sector.

The etiology of this disease is complex; *Fusarium* sp., *Alternaria* sp., *Phomopsis* sp., and *Botryosphaeriaceae* sp. have been identified as the predominant pioneer pathogens [4,5,6,7], often co-occurring with other agents such as *Lasiodiplodia theobromae*, *Neofusicoccum parvum*, *Penicillium expansum*, and *Botrytis cinerea* [8,9,10]. Currently, disease management remains heavily reliant on synthetic fungicides. Agents such as copper succinate, Amobam, hexaconazole, and trifloxystrobin-tebuconazole combinations are widely deployed [11,12,13]. Notably, the combination of calcium and azoxystrobin has shown efficacy, achieving control rates of up to 67.24% under optimal conditions. Concurrently, alternative strategies involving gaseous signaling molecules like hydrogen sulfide (H_2_S) and natural antifungal agents like natamycin have demonstrated potential in inducing systemic resistance or exerting direct antifungal activity [14,15]. Despite these interventions, the long-term, intensive application of chemical fungicides has precipitated severe adverse consequences, including the emergence of pathogen resistance and the disruption of the orchard micro-ecological balance. This has triggered a vicious cycle of disease recurrence and chemical escalation, exacerbating environmental contamination and raising alarming food safety concerns regarding residue accumulation. In response to the growing global imperative for ecological safety and food quality, there is an urgent need to transition toward environmentally sound and economically viable biological control strategies.

Recent progress in biological control has focused largely on bacteria and yeasts. For instance, the fermentation broth of the endophytic bacterium *Bacillus amyloliquefaciens* M9 and the cell-free supernatant of Bacillus subtilis BS-1 have shown efficacy in inhibiting *Botryosphaeria dothidea* and maintaining fruit quality [16,17]. Similarly, the yeast *Meyerozyma guilliermondii* 39 has proven effective as a postharvest agent by boosting host defense enzymes [18]. Although recent studies have demonstrated that postharvest biocontrol can suppress kiwifruit decay through direct antibiosis, nutrient and space competition, volatile or diffusible antifungal metabolites, and induction of host defense responses, the existing literature remains fragmented in several important respects. Most investigations have focused on a single bacterial or yeast antagonist and one dominant pathogen, particularly *Botryosphaeria dothidea*, *Alternaria alternata*, *Botrytis cinerea*, or *Diaporthe* sp., rather than addressing the pathogen complex associated with kiwifruit soft rot [15,19,20,21]. In addition, fungal antagonists remain substantially less explored than bacterial and yeast biocontrol agents. The available studies on the relationships among in vitro antagonism, in vivo fruit protection, fermentation-broth-mediated inhibition, and host defense responses within the same experimental framework remain insufficiently integrated.

Therefore, the present study aims to investigate antagonistic fungi isolated from kiwifruit tissues and rhizosphere soil, and to evaluate their biocontrol potential against four major pathogen groups associated with postharvest soft rot, including *Fusarium* sp., *Alternaria* sp., *Botryosphaeriaceae* sp., and *Phomopsis* sp. By integrating broad-spectrum antifungal assessment with analyses of antagonistic mechanisms and host-resistance induction, this study seeks to bridge the gap between the discovery of individual biocontrol strains and the development of a mechanistically supported, broad-spectrum biological control strategy for kiwifruit soft rot.

## 2. Materials and Methods

### 2.1. Isolation and Purification of Antagonistic Fungi from Kiwifruit Tissues and Rhizosphere Soil

Samples (leaves, bark and rhizosphere soil) were collected from organic orchards in Guizhou Province, China (no pesticides for >3 years). Plant tissues were surface-sterilized (1% NaOCl for 10 min, 75% ethanol for 30 s, sterile water rinse ×4), transferred to PDA (Hope-Bio, Qingdao, Shandong, China) plates, and incubated at 28 °C for 7 days [22]. Soil fungi were isolated using a dilution method [23]: 10 g soil in 90 mL water, shaken (180 rpm, 1 h), diluted (10^−3^–10^−6^ g/mL), plated on PDA (100 µL), incubated (28 °C, 7 days). Colonies were purified via monosporic isolation.

### 2.2. Screening for Antagonistic Fungi as Biocontrol Agents Through Dual-Culture Assays

The dual-culture assay [24] was used to screen antagonistic fungi. Four pathogenic fungal strains responsible for kiwifruit soft rot, namely *Fusarium* sp., *Alternaria* sp., *Phomopsis* sp. and *Botryosphaeriaceae* sp., were sourced from the culture collection of the authors’ laboratory, where they had been previously isolated and preserved. Pathogen mycelial plugs (5 mm) were placed on PDA plates with antagonistic fungal plugs inoculated 2 cm away. Controls contained only pathogens. All treatments were triplicated and incubated at 28 ± 2 °C for 5 days.

### 2.3. Determination of the Inhibition of Antagonistic Fungi on the Pathogenic Fungi In Vivo

Kiwifruits (*Actinidia chinensis* cv. Hongyang) at commercial maturity, weighing 80–100 g and free from mechanical damage, visible lesions, and surface defects, were selected and surface-disinfected with 1% (*v*/*v*) sodium hypochlorite for 10 min, followed by 75% (*v*/*v*) ethanol for 30 s, and rinsed three times with sterile distilled water. Uniform wounds (3 mm diameter × 3 mm depth) were created on the equatorial surface of each fruit using a sterile cork borer. For the treatment group, each wound was first inoculated with 10 μL of antagonist spore suspension (1 × 10^7^ spores/mL), whereas control wounds received no antagonist treatment. After incubation at ambient temperature for 2 h, 10 μL of pathogen spore suspension (1 × 10^7^ spores/mL) was applied to all wounds. Inoculated fruits were placed in plastic boxes and incubated at 28 ± 1 °C under 80–85% relative humidity. Each treatment consisted of three biological replicates with six fruits per replicate (18 fruits per treatment), and pathogen-only inoculated fruits served as the negative control. Lesion diameters were measured on days 3, 5, and 7 post-inoculation [25].

### 2.4. Antifungal Activity of Fermentation Broths Against Pathogenic Fungi

Fermentation broths from antagonistic fungi cultured in Sabouraud Dextrose Broth (SDB; Shanghai Bio-way technology Co., Ltd., Shanghai, China; 5 g/L tryptone, 5 g/L animal tissue digest,20 g/L glucose, pH 5.4–5.8), Potato Dextrose Broth (PDB; Shanghai Bio-way technology Co., Ltd., Shanghai, China; 5 g/L potato infusion powder, 20 g/L glucose, 1 g/L chloramphenicol, pH 5.8–6.2) and Martin-Modified Medium (MMM; Shanghai Bio-way technology Co., Ltd., Shanghai, China; 5 g/L peptone, 2 g/L yeast extract powder, 20 g/L glucose, 1 g/L K_2_HPO_4_, 0.5 g/L MgSO_4_, pH 6.2~6.6). For fermentation, 100 mL of each medium was dispensed into 250 mL Erlenmeyer flasks. A 5 mm diameter mycelial plug of purified antagonistic fungus was inoculated into each flask. The flasks were incubated in a rotary shaker at 180 rpm and 28 °C for 7 days. After fermentation, the crude culture liquid was first vacuum-filtered with a Buchner funnel to remove fungal mycelia and spores. The resulting filtrate was further sterilized using 0.22 μm membrane filters (Millipore, Burlington, MA, USA, GSWP02500) to obtain sterile fermentation broths. Kiwifruits (*Actinidia chinensis* cv. Hongyang) at commercial maturity, weighing 80–100 g and free from mechanical damage, visible lesions, and surface defects, were surface-disinfected with 0.1% (*v*/*v*) NaOCl for 1 min, followed by 75% (*v*/*v*) ethanol for 2–3 min, and rinsed with sterile distilled water. Uniform wounds (3 mm diameter × 3 mm depth) were created on the equatorial surface of each fruit. Each wound was first treated with 10 μL of sterile fermentation broth, while the control received an equal volume of sterile water. After 2 h at ambient temperature, 10 μL of pathogen spore suspension (1 × 10^7^ spores/mL) was applied to all wounds. Inoculated fruits were incubated at 28 °C under 90–95% relative humidity. Each treatment consisted of three biological replicates, each containing six fruits (18 fruits per treatment). Disease incidence and lesion diameters were recorded 7 days post-inoculation [26].

### 2.5. Enzyme Activities

Kiwifruit samples for enzyme assays were prepared following the spore suspension inoculation procedure described above, with three groups established for each assay: a blank control consisting of healthy, unwounded fruit without any inoculation, a pathogen-only control in which wounds were inoculated solely with the pathogen spore suspension, and a co-inoculation treatment in which wounds received the antagonist spore suspension first, followed by the pathogen after 2 h, exactly as in the in vivo inhibition assay. At each sampling time point (days 3, 5, and 7 post-inoculation), fruit tissues surrounding the wound site were excised, immediately snap-frozen in liquid nitrogen, and stored at −80 °C until analysis. For enzyme extraction, frozen tissue was homogenized on ice in a pre-chilled mortar with the appropriate extraction buffer and centrifuged at 12,000× *g* for 30 min at 4 °C. The supernatant was collected for spectrophotometric assays. Superoxide dismutase (SOD) was extracted from 2.0 g of tissue in 5 mL of ice-cold buffer (0.1 mM EDTA, 1 mM ascorbic acid, 2% PVPP, all reagents were purchased from Shanghai Macklin Biochemical Co., Ltd., Shanghai, China), with one unit defined as the amount of enzyme inhibiting the photochemical reduction in NBT by 50% per minute per gram of fresh weight [27]. Ascorbate peroxidase (APX) was extracted from 1.0 g of tissue in 5 mL of the same buffer, and one unit was defined as a decrease of 0.01 in absorbance at 290 nm per minute per gram of fresh weight [28]. Catalase (CAT) was extracted from 3.0 g of tissue in 5 mL of ice-cold buffer (5 mM DTT, 5% PVP, all reagents were purchased from Shanghai Macklin Biochemical Co., Ltd., Shanghai, China), with one unit corresponding to a decrease of 0.01 in absorbance at 240 nm per minute per gram of fresh weight [29]. Phenylalanine ammonia-lyase (PAL) was extracted from 4.0 g of tissue in 5 mL of ice-cold buffer (40 g/L PVP, 2 mM EDTA, 5 mM β-mercaptoethanol, Shanghai Yuanye Bio-Technology Co., Ltd., Shanghai, China), and one unit was defined as an increase of 0.01 in absorbance at 290 nm per hour per gram of fresh weight [30]. All enzyme activities were expressed as U/g fresh weight. Reactions were conducted at 25 °C in a total volume of 3 mL using 1 cm path-length cuvettes, with uniform illumination maintained for the SOD NBT photoreduction assay and continuous absorbance recording at the respective wavelengths for CAT, APX, and PAL to monitor activity changes.

### 2.6. Identification of Antagonistic Fungi from Kiwifruit Through Polyphasic Analysis

Mycelial plugs (5 × 5 mm) were inoculated on PDA plates, incubated at 28 °C for 5–7 days to observe colony characteristics. After 7–14 days of incubation, mycelia and spores were examined microscopically. Genomic DNA was extracted using the Rapid Fungi Genomic DNA Isolation Kit (Sangon Biotech, Shanghai, China). PCR amplification targeted the ITS region and specific primers (Table 1) using a 25 µL reaction mix: 1 µL DNA, 0.5 µL each primer, 12.5 µL Taq PCR Master Mix, and ddH_2_O. Cycling: 95 °C for 5 min; 35 cycles of 95 °C/30 s, 50–55 °C/30 s, 72 °C/1 min; 72 °C/5 min. PCR products were commercially sequenced, and the obtained sequences were subjected to homology searches usingthe BLASTN algorithm via the online NCBI BLAST server (https://blast.ncbi.nlm.nih.gov, accessed on 17 June 2026) with default parameters against the non-redundant (nr) nucleotide database. Reference sequences showing more than 90% similarity to the obtained sequences were selected for phylogenetic analysis. Sequence alignment was performed using MEGA 11. Different gene loci were concatenated in PhyloSuite, and a Bayesian phylogenetic tree was reconstructed using MrBayes based on the combined multilocus dataset [31]. The concatenation order was calmodulin + ITS for strain Pb-2-2, actin + ITS for strain Nd-1-1, and CFS + mcm7 + ITS for strain N2-1-1.

### 2.7. Statistical Analysis

All statistical analyses were performed to evaluate the significance of the observed effects. The lesion diameter and enzyme activity data were analyzed with GraphPad Prism (version 9.5, GraphPad Software, San Diego, CA, USA) using a two-way ANOVA, factoring in treatment and time, followed by Tukey’s multiple range test at *p* < 0.05.

## 3. Results

### 3.1. Isolation and Screening of Kiwifruit Soft Rot Antagonistic Fungi

Dual-culture assays showed that, among the 37 fungal isolates obtained from kiwifruit, N2-1-1, Nd-1-1, and Pb-2-2 displayed the strongest and most consistent antagonistic activity against the four soft rot pathogens tested, including *Fusarium* sp., *Alternaria* sp., *Botryosphaeriaceae* sp., and *Phomopsis* sp. (Table 2).

**Figure 1:**
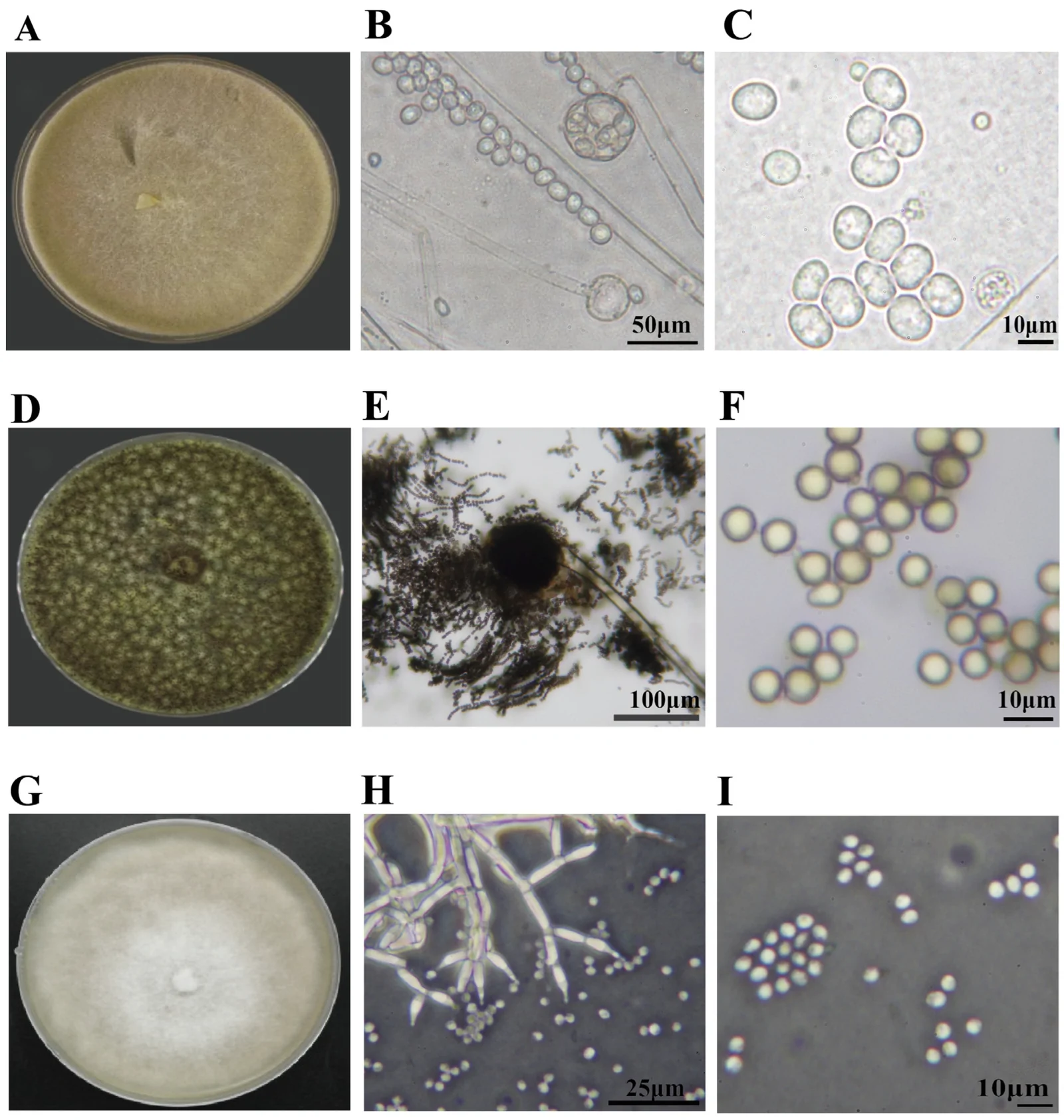
Morphological characteristics of the three antagonistic fungal isolates. Note: (**A**–**C**) Strain N2-1-1, showing a cottony colony, aseptate hyphae, and spherical to ellipsoidal sporangiospores. (**D**–**F**) Strain Pb-2-2, showing grayish-green to yellowish-green colonies and spherical to subglobose conidia arranged in chains. (**G**–**I**) Strain Nd-1-1, showing white cottony colonies and phialides producing spherical to subglobose conidia. Scale bars: (**B**) = 50 μm, (**C**) = 10 μm, (**E**) = 100 μm, (**F**) = 10 μm, (**H**) = 25 μm, and (**I**) = 10 μm.

**Figure 2:**
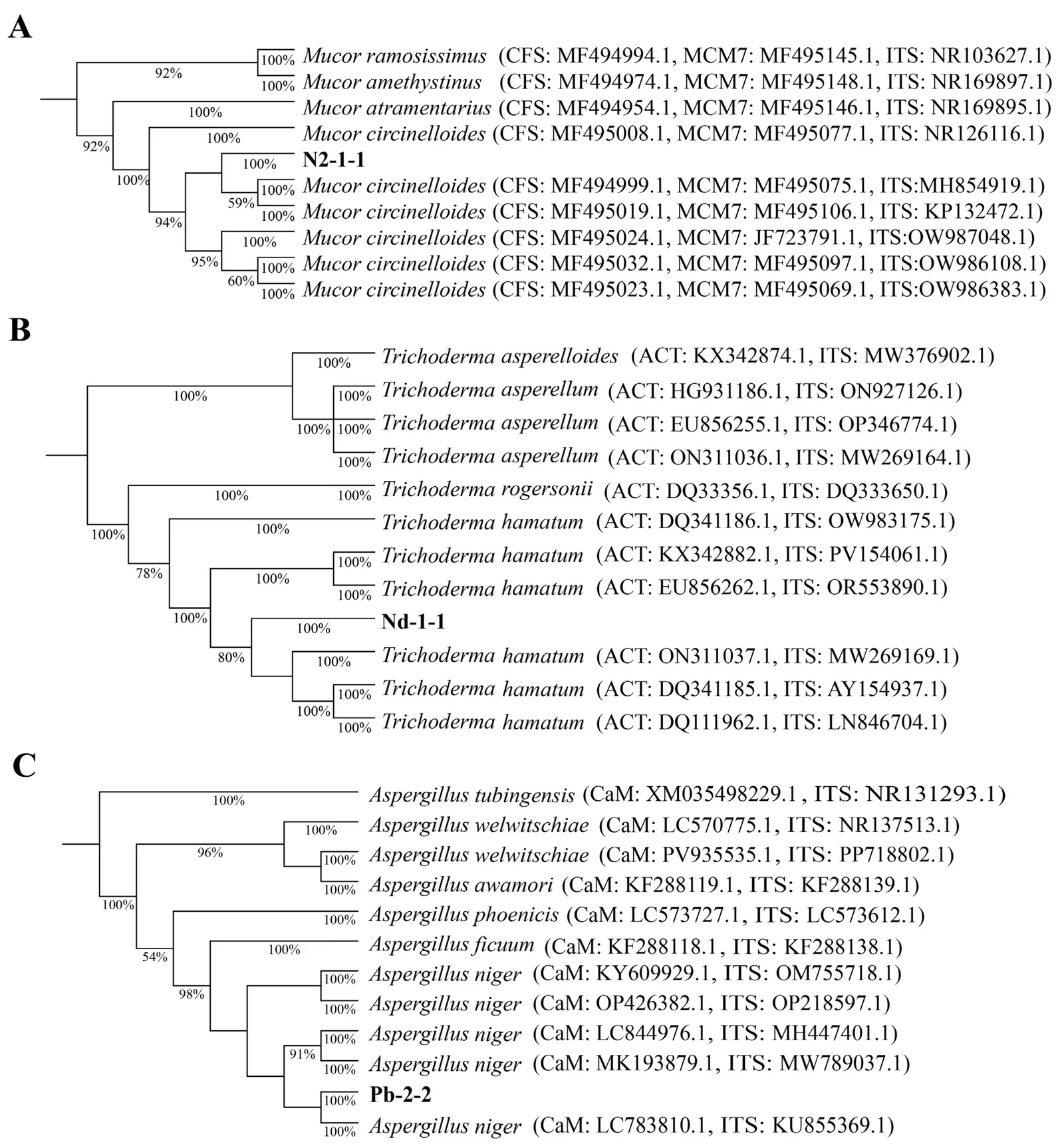
Phylogenetic identification of the three antagonistic fungal isolates based on multilocus sequence analysis. Note: (**A**) Strain N2-1-1, inferred from concatenated CFS, *mcm*7 and rDNA-ITS gene sequences. (**B**) Strain Nd-1-1, inferred from concatenated actin and rDNA-ITS gene sequences. (**C**) Strain Pb-2-2, inferred from concatenated calmodulin and rDNA-ITS gene sequences. Bootstrap support values from 1000 replicates are shown at the nodes; only values >50% are shown. Accession numbers are provided after strain names.

The three antagonistic fungi were identified through a combination of morphological and phylogenetic analyses. Strain N2-1-1 was identified as *Mucor circinelloides*, exhibiting cottony colonies with aseptate hyphae and spherical to ellipsoidal sporangiospores [37] (Figure 1A–C), and was confirmed based on rDNA-ITS (PX396488), CFS (PV976146), and mcm7 (PV976147) gene sequences (Figure 2A). Strain Pb-2-2 was identified as *Aspergillus niger*, with grayish green to yellowish green colonies and spherical to subglobose conidia arranged in chains [38] (Figure 1D–F), and this was supported by rDNA-ITS (PX396490) and calmodulin (PV976151) gene sequences (Figure 2B). Strain Nd-1-1 was identified as *Trichoderma hamatum*, showing white to green cottony colonies and producing phialides with spherical to subglobose conidia [39] (Figure 1G–I), and was determined based on rDNA-ITS (PX396489) and actin (PV976149) gene sequences (Figure 2C).

### 3.2. Effects of Spore Suspensions of Antagonistic Fungi on Pathogenic Fungi of Kiwifruit Soft Rot In Vivo

**Figure 3:**
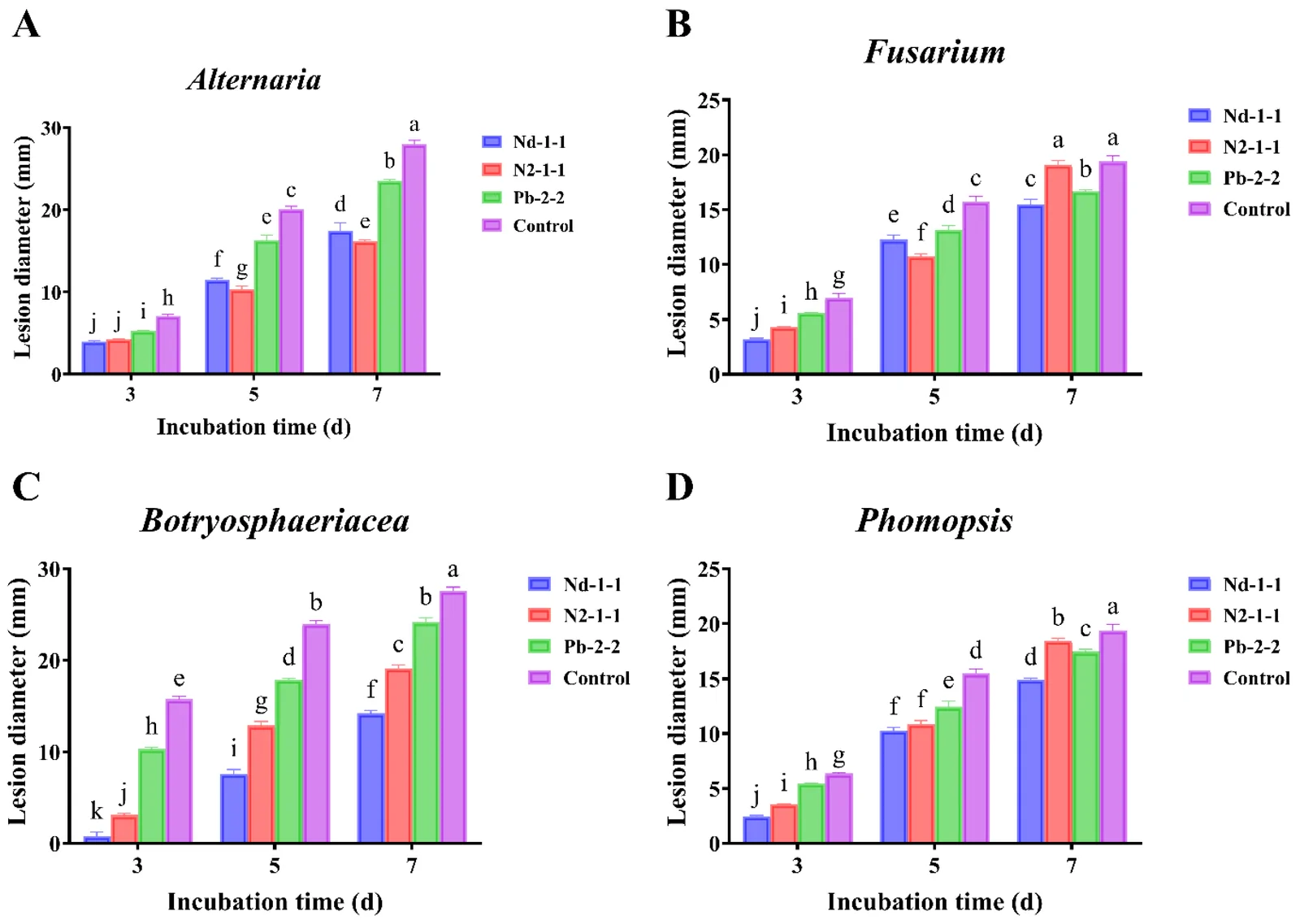
Inhibitory effects of spore suspensions of three antagonistic fungi on lesion development caused by four soft rot pathogens in kiwifruit. Note: Lesion diameters on kiwifruit caused by (**A**) Alternaria, (**B**) Phomopsis, (**C**) Fusarium, and (**D**) Botryosphaeriaceae were determined after treatment with spore suspensions of three antagonistic fungi (Nd-1-1, N2-1-1, Pb-2-2) at 3, 5 and 7 days post-inoculation in vivo. All data are presented as mean ± standard deviation (SD, *n* ≥ 3). Different lowercase letters above the bars indicate significant differences among treatments at the same incubation time, as determined by two-way ANOVA followed by Tukey’s multiple range test (*p* < 0.05) (Table A1 and Table A4).

In the spore suspension inoculation assay, the three antagonistic strains exhibited distinct temporal patterns of disease suppression across the four pathogens. Against *Alternaria* sp. (Figure 3A), *M. circinelloides* N2-1-1 consistently restricted lesion expansion from day 3 onward, outperforming *T. hamatum* Nd-1-1 by day 7, while *A. niger* Pb-2-2, despite an exceptionally stable and reproducible inhibition, allowed a late-stage surge in lesion development that markedly diminished its overall efficacy. In contrast, *T. hamatum* Nd-1-1 established early dominance against the remaining three pathogens and sustained the strongest suppression through the experimental period. For *Fusarium* sp. (Figure 3B), *T. hamatum* Nd-1-1 maintained lesions at a notably lower level than the other strains and the control, with *A. niger* Pb-2-2 displaying a moderate, tightly clustered response that contrasted with the progressive weakening observed in *M. circinelloides* N2-1-1. Against *Botryosphaeriaceae* sp. (Figure 3C), *T. hamatum* Nd-1-1 exerted the most powerful and sustained control, virtually preventing lesion progression, whereas *M. circinelloides* N2-1-1 and *A. niger* Pb-2-2 proved progressively less effective, with the latter showing only marginal reduction relative to the control. A similar hierarchy was evident for *Phomopsis* sp. (Figure 3D), where *T. hamatum* Nd-1-1 again limited lesion size most effectively, while the other two strains converged toward the control level over time. Notably, although *A. niger* Pb-2-2 was rarely the most suppressive strain, it consistently produced the least variable lesion diameters across replicates, confirming a uniquely stable antagonistic phenotype.

### 3.3. Effects of Fermentation Broth on Pathogenic Fungi of Kiwifruit Soft Rot

**Figure 4:**
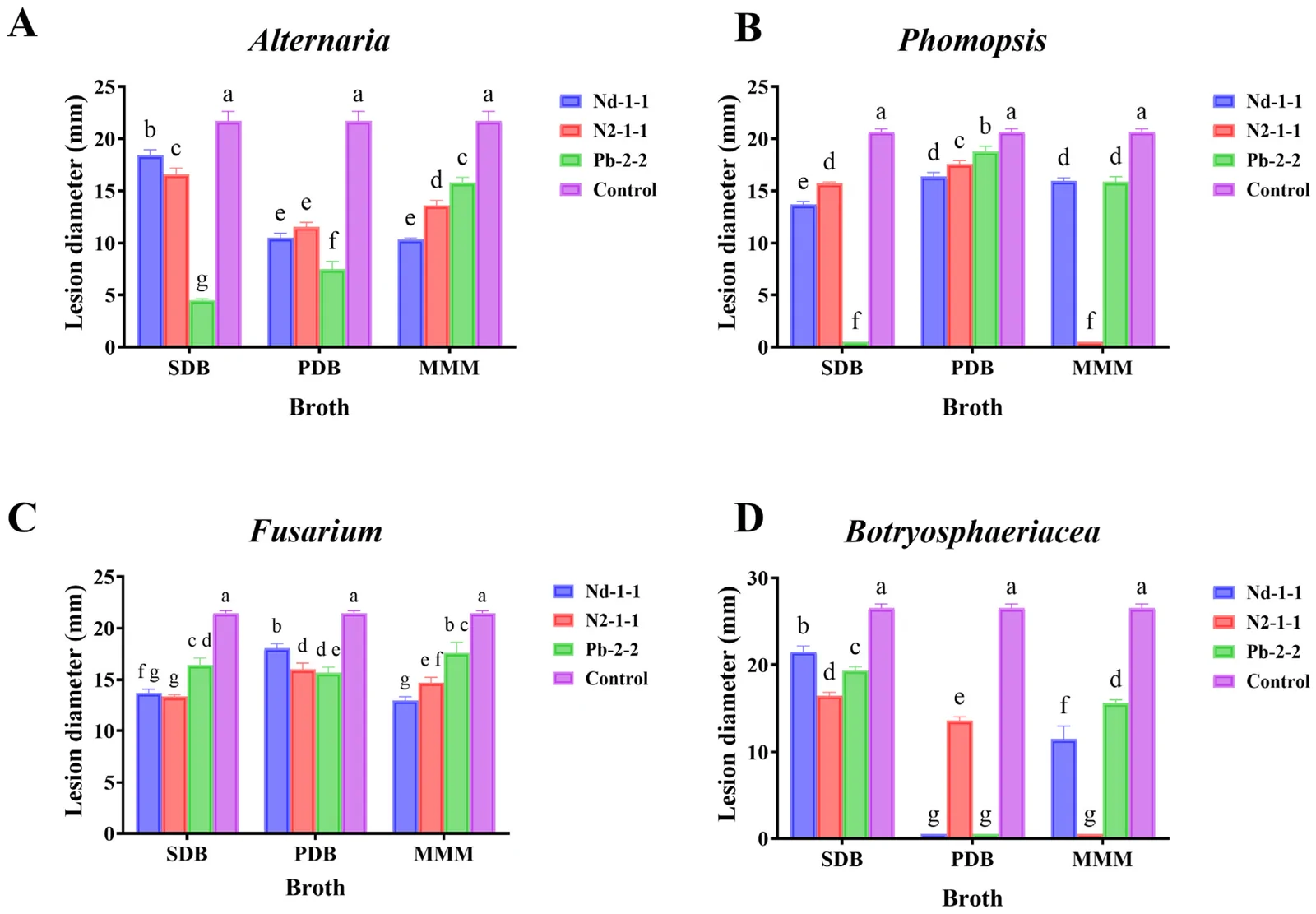
Inhibitory effects of fermentation broth from three antagonistic fungi cultured in different media on lesion expansion caused by four soft rot pathogens in kiwifruit. Note: Lesion diameters on kiwifruit caused by (**A**) Alternaria, (**B**) Phomopsis, (**C**) Fusarium, and (**D**) Botryosphaeriaceae were determined after treatment with fermentation broth of three antagonistic fungi (Nd-1-1, N2-1-1, Pb-2-2) grown in three different media: SDB (Sabouraud Dextrose Broth), PDB (Potato Dextrose Broth), and MMM (Minimal Medium). All data are presented as mean ± standard deviation (SD, *n* ≥ 3). Different lowercase letters above the bars indicate significant differences among treatments within the same medium, as determined by two-way ANOVA followed by Tukey’s multiple range test (*p* < 0.05) (Table A2 and Table A5).

Fermentation assays confirmed that all three antagonistic fungi secreted antimicrobial metabolites capable of reducing lesion diameter relative to the control, yet the extent of suppression varied dramatically with strain, culture medium, and pathogen identity. Complete arrest of lesion development was restricted to specific combinations. The SDB filtrate of *A. niger* Pb-2-2 and the MMM filtrate of *M. circinelloides* N2-1-1 abolished disease caused by *Phomopsis* sp. (Figure 4B), while against *Botryosphaeriaceae* sp. (Figure 4D), such thorough inhibition additionally required the PDB filtrate of *T. hamatum* Nd-1-1. In most other pairings, the filtrates afforded only partial protection, and in a subset of cases the reduction was marginal, most conspicuously for *T. hamatum* Nd-1-1 in SDB against *Botryosphaeriaceae* sp., where lesions remained large despite treatment. The impact of the culture medium was especially evident for *T. hamatum* Nd-1-1; its PDB filtrate could eliminate *Botryosphaeriaceae* lesions entirely, whereas its SDB filtrate was among the least effective treatments across the whole dataset (Figure 4D). Among the four pathogens, *Fusarium* sp. (Figure 4C) proved consistently the least responsive; no treatment achieved lesion clearance, and even the strongest filtrates only moderately slowed expansion, leaving lesion sizes well above those recorded for other pathogens. Conversely, *Alternaria* sp. (Figure 4A) was highly susceptible to *A. niger* Pb-2-2 filtrates, particularly in SDB, which nearly prevented symptom development, and the same strain in PDB also exerted strong suppression. *T. hamatum* Nd-1-1 and *M. circinelloides* N2-1-1 provided moderate control against *Alternaria*, with their PDB and MMM filtrates outperforming other media. For *Phomopsis* sp., the exceptionally low lesion diameters achieved by *A. niger* Pb-2-2 in SDB and M. *circinelloides* N2-1-1 in MMM contrasted with the more modest effects of *T. hamatum* Nd-1-1, whose best performance occurred in SDB.

### 3.4. Effects of Antagonistic Fungi on the Resistance of Kiwifruit Fruits

**Figure 5:**
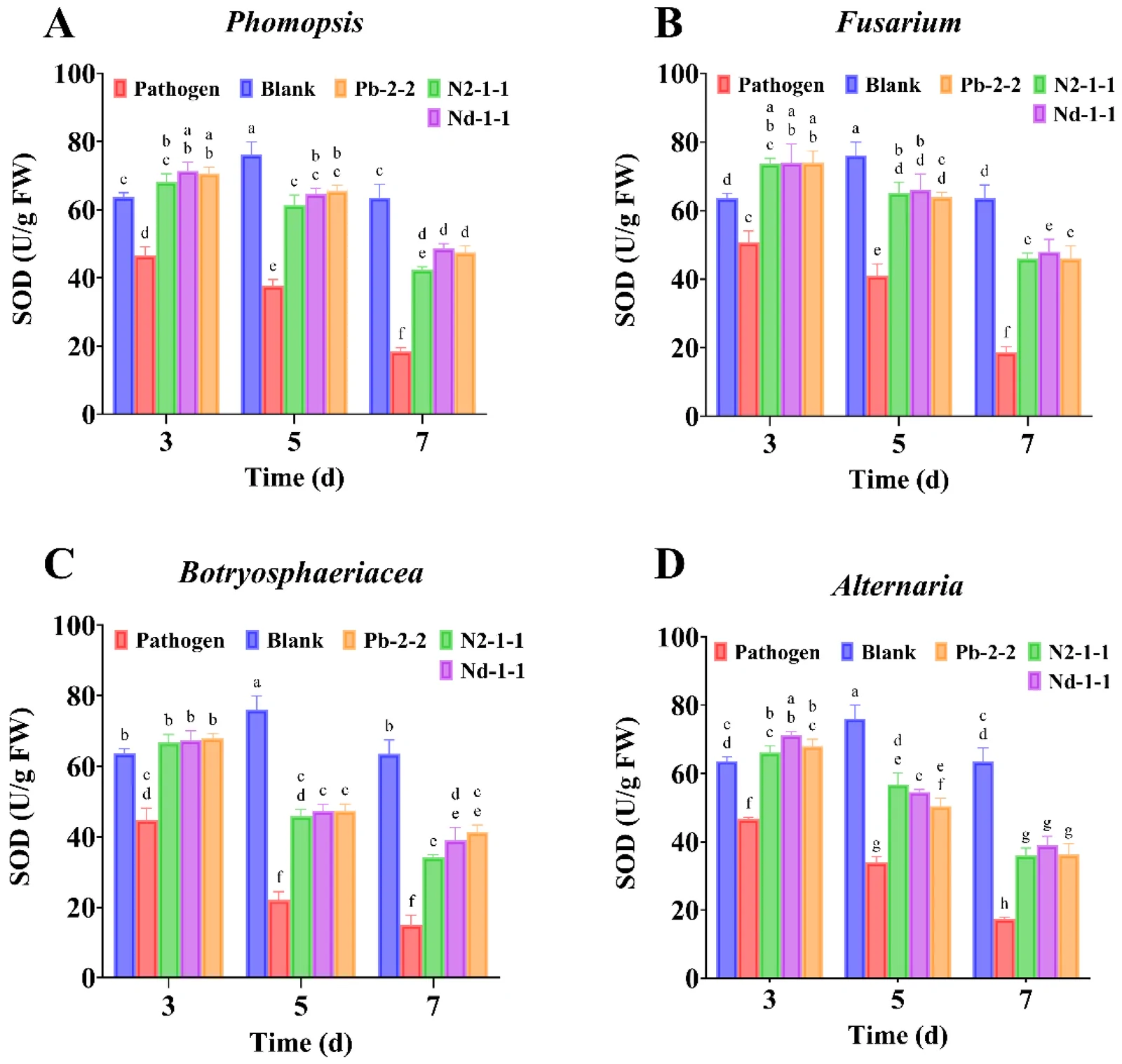
Effects of three antagonistic fungi on SOD enzyme activity in kiwifruit fruits inoculated with four soft rot pathogens. Note: SOD enzyme activity (U/g FW) was measured in kiwifruit fruits after inoculation with different pathogens. The pathogens were: (**A**) Phomopsis, (**B**) Fusarium, (**C**) Botryosphaeriaceae, and (**D**) Alternaria. Treatments included a blank control (Blank) and three antagonistic fungi (Pb-2-2, N2-1-1, Nd-1-1). All data are presented as mean ± standard deviation (SD, *n* ≥ 3). Different lowercase letters above the bars indicate significant differences among treatments within the same pathogen, as determined by two-way ANOVA followed by Tukey’s multiple range test (*p* < 0.05).

**Figure 6:**
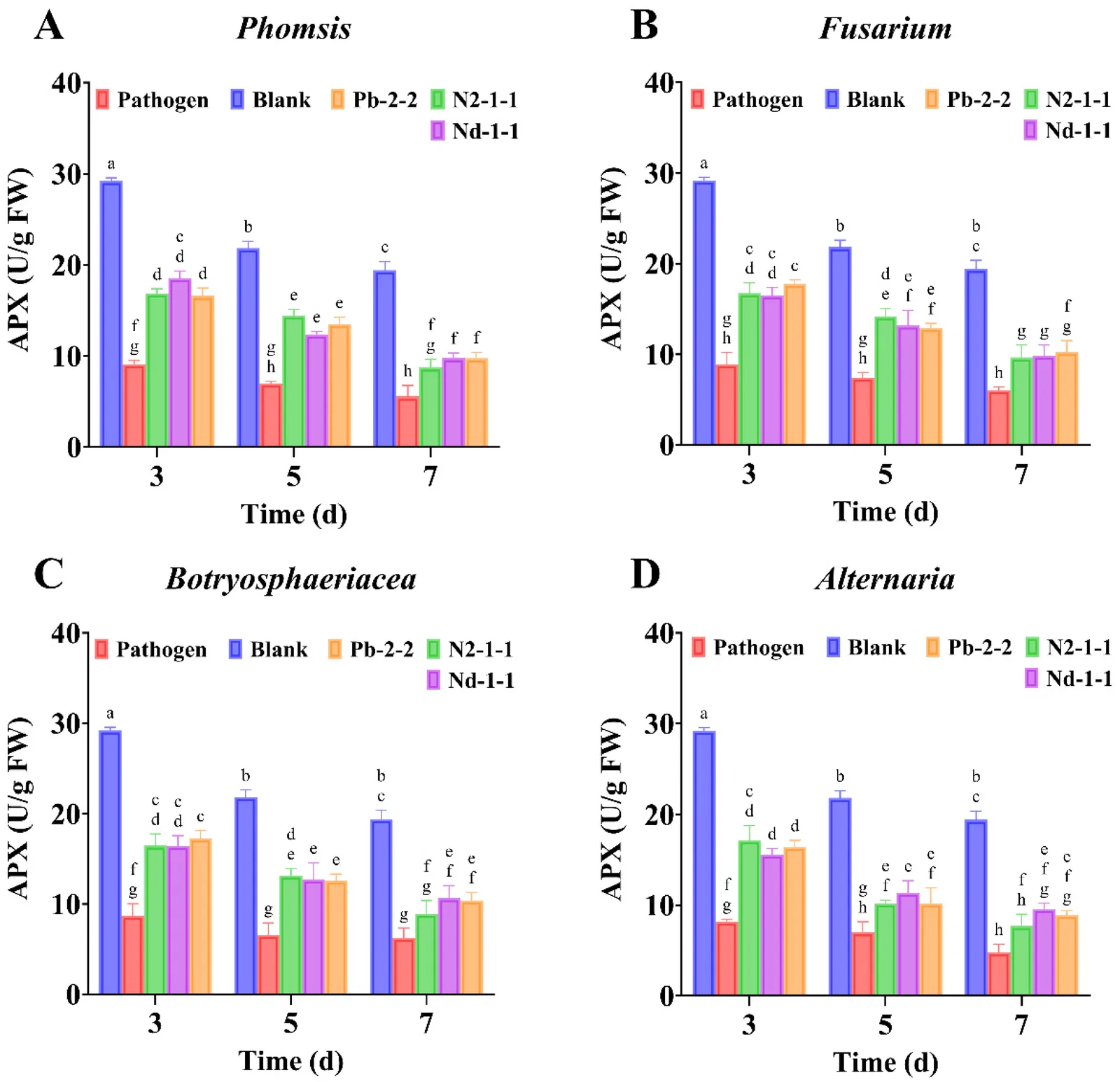
Effects of three antagonistic fungi on APX enzyme activity in kiwifruit fruits inoculated with four soft rot pathogens. Note: APX enzyme activity (U/g FW) was measured in kiwifruit fruits after inoculation with different pathogens. The pathogens were: (**A**) Phomopsis, (**B**) Fusarium, (**C**) Botryosphaeriaceae, and (**D**) Alternaria. Treatments included a blank control (Blank) and three antagonistic fungi (Pb-2-2, N2-1-1, Nd-1-1). All data are presented as mean ± standard deviation (SD, *n* ≥ 3). Different lowercase letters above the bars indicate significant differences among treatments within the same pathogen, as determined by two-way ANOVA followed by Tukey’s multiple range test (*p* < 0.05).

To evaluate how biocontrol fungi modulate kiwifruit defenses, SOD, CAT, APX and PAL activities were monitored following pathogen inoculation alone and antagonist–pathogen co-inoculation. Statistical analyses indicated highly significant effects of treatment, time and their interaction on all enzymes assayed (Table A3, Table A6, Table A7, Table A8 and Table A9). Relative to the blank group, pathogen inoculation alone significantly reduced SOD (Figure 5) and APX (Figure 6) activities, indicating compromised antioxidant capacity and perturbed ROS homeostasis under infection pressure, whereas co-inoculation effectively maintained SOD and APX activities, suggesting mitigation of oxidative stress and improved maintenance of ROS balance.

**Figure 7:**
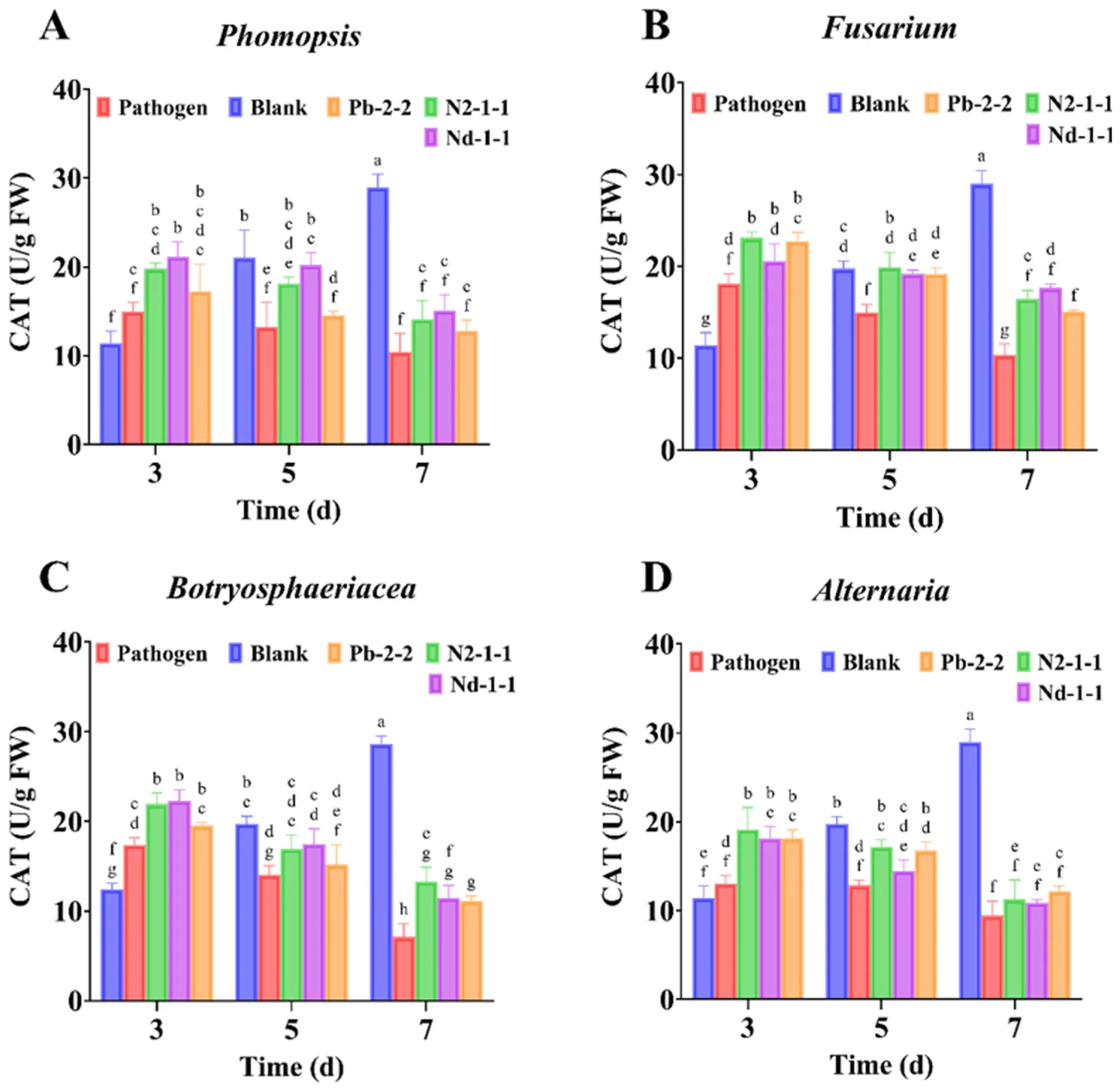
Effects of three antagonistic fungi on CAT enzyme activity in kiwifruit fruits inoculated with four soft rot pathogens. Note: CAT enzyme activity (U/g FW) was measured in kiwifruit fruits after inoculation with different pathogens. Note: (**A**) *Phomopsis*, (**B**) *Fusarium*, (**C**) *Botryosphaeriaceae*, and (**D**) *Alternaria*. Treatments included a blank control (Blank) and three antagonistic fungi (Pb-2-2, N2-1-1, Nd-1-1). All data are presented as mean ± standard deviation (SD, *n* ≥ 3). Different lowercase letters above the bars indicate significant differences among treatments within the same pathogen, as determined by two-way ANOVA followed by Tukey’s multiple range test (*p* < 0.05).

**Figure 8:**
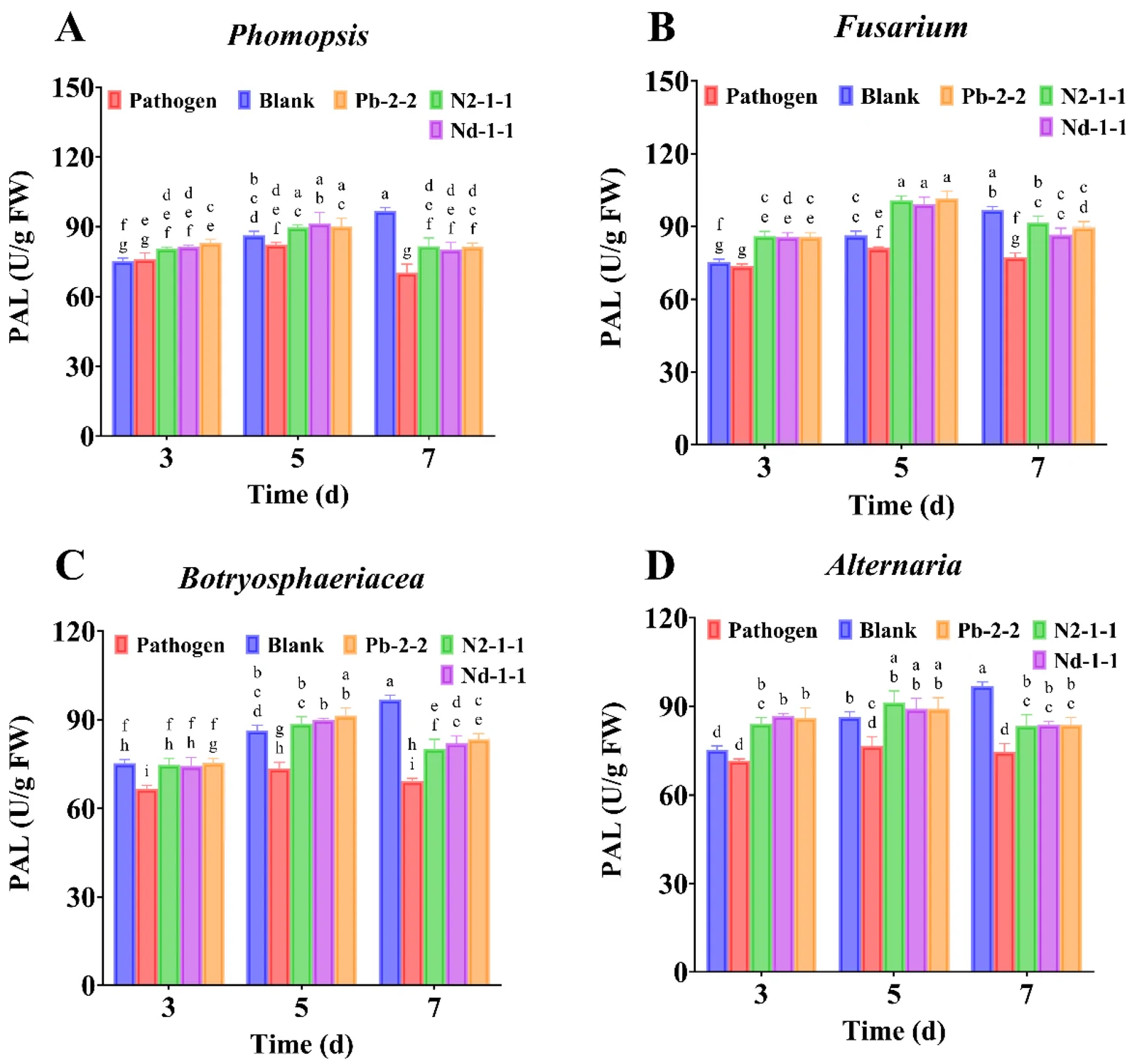
Effects of three antagonistic fungi on PAL enzyme activity in kiwifruit fruits inoculated with four soft rot pathogens. Note: PAL enzyme activity (U/g FW) was measured in kiwifruit fruits after inoculation with different pathogens. The pathogens were: (**A**) *Phomopsis*, (**B**) *Fusarium*, (**C**) *Botryosphaeriaceae*, and (**D**) *Alternaria*. Treatments included a blank control (Blank) and three antagonistic fungi (Pb-2-2, N2-1-1, Nd-1-1). All data are presented as mean ± standard deviation (SD, *n* ≥ 3). Different lowercase letters above the bars indicate significant differences among treatments within the same pathogen, as determined by two-way ANOVA followed by Tukey’s multiple range test (*p* < 0.05).

CAT (Figure 7) exhibited distinct temporal patterns between non-inoculated and inoculated fruit: CAT increased steadily in the blank group, whereas it increased initially and then declined continuously under pathogen-only inoculation, becoming significantly lower than the blank group by day 7. Under co-inoculation, CAT followed a similar trajectory to the pathogen-only treatment but was significantly higher at day 3, indicating a transient enhancement of H_2_O_2_-scavenging capacity early during infection (Figure 7). For phenylpropanoid metabolism, PAL (Figure 8) responses were pathogen-dependent and stage-dependent: *Botryosphaeriaceae* sp. and *Alternatia* sp. reduced PAL at the early stage (day 3), while *Fusarium* sp. and *Phomopsis* sp. decreased PAL mainly at middle and late stages (days 5–7); under co-inoculation, PAL generally increased initially and then declined (Figure 7). Notably, no significant differences were detected among the three antagonistic fungal strains (*T. hamatum* Nd-1-1, *M. circinelloides* N2-1-1, and *A. niger* Pb-1-1) in their capacity to maintain the activities of SOD, APX, and PAL. Although inter-strain variation was observed in the magnitude of early-stage (day 3) CAT activity enhancement, the overall temporal trends in modulating the four defense enzyme activities were highly consistent across all three antagonists. This finding suggests that these distinct biocontrol agents may elicit convergent host defense outputs and/or that the fruit defense response has reached a plateau-like saturation level. Although co-inoculation generally maintained host defense-related enzyme activities, these parameters did not clearly discriminate among the three antagonists, suggesting that differences in in vivo efficacy may depend more on ecological establishment and direct antagonistic traits than on final enzyme levels alone.

## 4. Discussion

Postharvest soft rot poses a serious and persistent threat to the extensive kiwifruit (*Actinidia chinensis*) industry in China, inflicting considerable economic damage. A promising alternative for control lies in the diverse native microorganisms that colonize kiwifruit surfaces, which serve as a valuable source of antagonistic microbes [40]. These naturally occurring yeasts, bacteria, and filamentous fungi exhibit inherent competitive advantages against pathogens in the postharvest environment, positioning them as safe and effective biocontrol agents [41]. This potential prompted the present study to isolate antagonistic fungi from kiwifruit, screen their biocontrol activity against soft rot pathogens, and preliminarily characterize their modes of action.

A total of 37 fungal isolates were obtained from kiwifruit leaves, bark, and rhizosphere soil, and their antagonistic activities against four major groups of soft-rot-associated pathogens, namely *Fusarium* sp., *Alternaria* sp., *Botryosphaeriaceae* sp., and *Phomopsis* sp., were evaluated. Among these isolates, *Mucor circinelloides* N2-1-1, *Aspergillus niger* Pb-2-2, and *Trichoderma hamatum* Nd-1-1 showed strong biocontrol potential. (Table 3).

Fungi from the genus *Mucor* are more frequently reported as pathogens [42,43] than as biocontrol agents, and their potential for biological control remains largely unexplored. *M. hiemalis* [44] was previously reported as an antagonist against inflorescence brown rot of date palm caused by *Thielaviopsis paradoxa*, where its protective effect was attributed primarily to competitive colonization and spatial exclusion on floral tissues, without evidence of phytopathogenicity. Similarly, *M. moelleri* [45] was further shown to possess both antagonistic activity toward plant pathogens and plant growth-promoting properties, highlighting the functional versatility of this genus. In addition, *Mucor moelleri* AA1 inhibited *Athelia rolfsii* and *Colletotrichum* spp. in dual-culture and liquid co-culture assays, reduced disease development in tomato pot experiments, and promoted plant growth. Volatile compounds, β-1,3-glucanase, protease, and catalase produced by this strain may jointly contribute to its antagonistic and growth-promoting effects [45]. Cell-free culture supernatant of *Mucor lusitanicus* inhibited *Fusarium oxysporum*, *F. solani*, and *Alternaria solani* and exerted antifungal effects in soil and tomato fruit. The Rfs enzyme involved in rhizoferrin biosynthesis affected the antifungal activity of the culture supernatant [46]. *Mucor circinelloides* N2-1-1 showed strong inhibitory activity in dual-culture assays, and its fermentation filtrate strongly inhibited *Botryosphaeriaceae* and *Phomopsis*; the present study expands the range of *Mucor* species with potential biocontrol activity.

*Aspergillus niger* can inhibit plant-pathogenic fungi through multiple mechanisms, including rapid growth and niche occupation, competition for nutrients and space, mycoparasitism, extracellular hydrolytic enzymes, and diffusible or volatile antifungal metabolites [47,48,49]. Previous studies have shown that *A. niger* hyphae can attach to, coil around, form penetration structures in, and damage *Fusarium* hyphae. In addition, the extracellular chitinase produced by *A. niger* LOCK 62 strongly inhibited *Fusarium culmorum* and F. *solani*, but showed little or no activity against *F. oxysporum*, *Alternaria alternata*, and *Botrytis cinerea* [50,51]. *A. niger* Pb-2-2 exhibited strong and relatively consistent inhibition against all four pathogen groups tested in the dual-culture assay. This suggests that inhibition by *A. niger* Pb-2-2 in the dual-culture system may have been mainly driven by rapid growth, niche occupation, and competition for nutrients and space. Compared with the dual-culture assay, the protective effect of the *A. niger* Pb-2-2 spore suspension in kiwifruit was substantially reduced, possibly because of limitations in spore germination and colonization of fruit tissues. Moreover, *A. niger* Pb-2-2 fermentation filtrate provided better protection against all four pathogens in inoculated kiwifruit, and its inhibition of lesion expansion remained strongly pathogen-dependent, with greater effects against *Alternaria*, *Phomopsis*, and *Botryosphaeriaceae* than against *Fusarium*. This result is consistent with previous reports showing selective inhibition of different pathogens by extracellular chitinase from *A. niger* LOCK 62 [50].

*Trichoderma* sp. is a kind of classical biocontrol fungus, and numerous studies have reported its role in the control of field diseases and postharvest fungal diseases of fruits and vegetables. *Trichoderma* sp. is generally considered to antagonize plant-pathogenic fungi through rapid growth and spatial occupation, nutrient competition, mycoparasitism, extracellular hydrolytic enzymes, and antimicrobial metabolites. For example, *Trichoderma* Tha739 can grow alongside and coil around *Colletotrichum* hyphae, resulting in hyphal deformation [52]. Strain T88 can penetrate and damage the hyphal cells of apple ring-rot pathogens through mycoparasitism, causing cytoplasmic thinning and structural damage [53]. In tomato fruit, *Trichoderma* can also cover, coil around, and degrade the sporangiophores and sporangia of *Rhizopus* [54].

Consistent with these findings, *T. hamatum* Nd-1-1 showed strong inhibitory activity in the dual-culture assay. Its inhibition rates against *Fusarium* sp., *Alternaria* sp., *Botryosphaeriaceae* sp., and *Phomopsis* sp. were 78.23%, 78.37%, 78.61%, and 60.10%, respectively. The weaker inhibition of *Phomopsis* suggests that this pathogen may be relatively less sensitive to the mycoparasitism, cell-wall-degrading enzymes, or other antagonistic factors produced by *T. hamatum* Nd-1-1.

Similar to *A. niger* Pb-2-2 and *M*. *circinelloides* N2-1-1, the direct protective effect of the *T. hamatum* Nd-1-1 spore suspension was substantially reduced in fruit. However, the Nd-1-1 spore suspension showed the highest inhibition among the three strains against *Fusarium* sp., *Botryosphaeriaceae* sp., and *Phomopsis* sp., and the second-highest activity against *Alternaria* sp. Thus, although the in vivo efficacy of *T. hamatum* Nd-1-1 spore suspension was lower than its activity in the dual-culture assay, its overall efficacy as a living spore preparation remained higher than that of *A. niger* Pb-2-2 and *M*. *circinelloides* N2-1-1. It suggests that *T. hamatum* Nd-1-1 may be better adapted to the kiwifruit environment and may have stronger spore germination, wound colonization, or local competitive ability than the other two strains.

Previous studies have shown that *Trichoderma* sp. can produce chitinase, β-1,3-glucanase, protease, and several potentially active compounds after recognizing pathogen cell walls, including 6-pentyl-α-pyrone, harzianic acid, isoharzianic acid, viridiofungin A, harzianopyridone, harzianolide, and peptaibols [55,56,57]. Synergistic interactions may occur between cell-wall-degrading enzymes and these active compounds. In the present study, *T. hamatum* Nd-1-1 fermentation filtrate inhibited *Fusarium* sp., *Alternaria* sp., *Botryosphaeriaceae* sp., and *Phomopsis* sp. by 45.73%, 65.56%, 98.11%, and 33.80%, respectively. Further studies could compare the antifungal activities of the original filtrate, pH-neutralized filtrate, heat-treated filtrate, and protease-treated filtrate, together with fractionation, metabolomic analysis, and extracellular enzyme assays, to identify the active components in *T. hamatum* Nd-1-1 fermentation filtrate.

SOD catalyzes the dismutation of superoxide radicals (O_2_•^−^) into oxygen and hydrogen peroxide (H_2_O_2_) [58]. APX has a high affinity for H_2_O_2_ and mainly removes low concentrations of H_2_O_2_ in chloroplasts and the cytoplasm [59], whereas CAT has a high processing capacity and primarily decomposes high concentrations of H_2_O_2_ in peroxisomes [60]. Following pathogen infection, fruit tissues generally produce superoxide anions and hydrogen peroxide, resulting in an early oxidative burst. Moderate levels of ROS can function as defense signals and participate in the activation of MAPK, SA, JA, and ethylene signaling pathways, whereas excessive ROS can cause membrane lipid peroxidation, membrane damage, and oxidative stress in fruit tissues [61]. In the present study, pathogen infection decreased SOD and APX activities in kiwifruit, whereas treatment with the antagonistic fungi partially alleviated these decreases and enhanced CAT activity. These results suggest that *M. circinelloides* N2-1-1, *A. niger* Pb-2-2, and *T. hamatum* Nd-1-1 may coordinate ROS homeostasis by maintaining SOD and APX activities and enhancing CAT responses. This may reduce excessive O_2_•^−^ accumulation and promote the timely removal of excess H_2_O_2_. In this process, an appropriate level of H_2_O_2_ may remain available as a defense signal, while excessive H_2_O_2_ can be efficiently decomposed by CAT, thereby reducing oxidative damage and maintaining redox homeostasis in fruit cells. Similarly, previous studies have shown that biocontrol microorganisms can enhance antioxidant enzyme systems in infected fruits and help maintain relatively stable ROS levels under pathogen stress [62,63].

PAL is a key rate-limiting enzyme in the phenylpropanoid pathway, and changes in its activity reflect the capacity of fruit tissues to synthesize phenolic compounds, lignin, and other defense-related secondary metabolites. Compared with pathogen-only inoculation, co-inoculation with the antagonistic fungi and pathogens maintained or increased PAL activity during the early stage of infection. At 3–5 days after inoculation, PAL activity generally approached, reached, or even exceeded that of uninoculated fruit. This indicates that the antagonistic fungi delayed pathogen-mediated suppression of phenylpropanoid metabolism and supported early defense responses in kiwifruit. Previous studies have shown that antagonistic fungi or yeasts can promote phenylpropanoid metabolism by increasing PAL activity and enhancing the synthesis of phenolic and other antimicrobial secondary metabolites [64,65].

However, during the late stage of infection, PAL activity in the co-inoculation treatments, although still significantly higher than that in pathogen-only fruit, generally failed to remain at or above the level of healthy controls. It is noteworthy that the effects of the three antagonistic fungi on SOD, CAT, APX, and PAL activities were not statistically different at most sampling times, which does not indicate that the three strains have identical biocontrol mechanisms. Rather, it may suggest that they regulate the fruit defense system in a partially convergent manner, whereas their main differences occur at the levels of direct antagonism, tissue colonization, and metabolite activity.

## 5. Conclusions

This study isolated, screened, and identified antagonistic fungi associated with kiwifruit and demonstrated that *Trichoderma hamatum* Nd-1-1, *Aspergillus niger* Pb-2-2, and *Mucor circinelloides* N2-1-1 exhibited significant antagonistic activity against the major fungal pathogens of kiwifruit soft rot disease. Among them, *A. niger* Pb-2-2 showed the strongest and most stable antifungal performance in vitro, whereas *T. hamatum* Nd-1-1 achieved the greatest disease suppression in vivo. All three antagonistic fungi showed strong and broad-spectrum antifungal activity in the dual-culture assay; however, their spore suspensions were substantially less effective in fruit than in vitro. This indicates that inhibition rates obtained from dual-culture assays cannot fully predict the practical efficacy of antagonistic fungi in fruit, which is also influenced by spore germination, wound colonization, and adaptation to the fruit environment. Fermentation assays further revealed that all three antagonists were capable of secreting antifungal secondary metabolites, although the inhibitory spectrum and stability of these metabolites were markedly influenced by culture medium, fermentation duration, and target pathogen. Moreover, these antagonistic fungi may enhance kiwifruit resistance to soft rot through modulation of host antioxidant defense systems and phenylpropanoid metabolism, as reflected by the maintenance of SOD and APX activities, the transient induction of CAT activity at the early stage of infection, and the upregulation of PAL activity. These responses likely contribute to the alleviation of oxidative damage and the activation of host defense.

Overall, our study identified three antagonistic fungi with biocontrol potential against multiple pathogens associated with kiwifruit soft rot. This is the first report of the biocontrol potential of *Mucor circinelloides* strain N2-1-1, thereby expanding the current understanding of the potential application of *Mucor* sp. in biological disease control. However, though reports on *Mucor* sp. as biocontrol agents remain limited, the potential of *Mucor circinelloides* N2-1-1 for controlling kiwifruit soft rot requires further investigation. Before practical application can be achieved, further studies are required to identify the active metabolites, optimize fermentation and formulation strategies, and validate efficacy under commercial storage conditions. In addition, comprehensive biosafety assessments, particularly regarding toxigenic potential, allergenicity, and storage safety, are essential to ensure safe and feasible application in sustainable postharvest management of kiwifruit soft rot.

## Figures and Tables

**Table 1:** Primers and their sequences for the identification of endophytic fungal species.

Gene	Primer	Sequence (5′-3′)
internal transcribed spacer region of nuclear ribosomal	ITS [32]	ITS1: TCCGTAGGTGAACCTCCGG
		ITS4: TCCTCCGCTTATTGATATGC
*cyclopropane-fatty-acyl-phospholipid synthase gene*	CFS [33]	F: TTYTCYCGIDEOXYITTYGCTCCTCGT
		R: ACCARATRAARTCYTCRTATTGCCA
*minichromosome maintenance complex component 7*	*mcm*7 [34]	F: ACIDEOXYIMGIDEOXYIGTIDEOXYITCVGAYGTHAARCC
		R: GAYTTDGCIDEOXYIACIDEOXYICCIDEOXYIGGRTCWCCCAT
calmodulin gene	CaM [35]	F: CCGACAAGGARGCCTTC
		R: CCGATRGGTCATRACGTGG
*a* *ctin*	*act* [36]	F: TGGCACCACACCTTCTACAATGA
		R: TCTCCTCTGCATACGGTCGGA

Note: The headings Primer and Sequence (5′-3′) are bolded to highlight the core column headings for clear identification and retrieval of primer names and their corresponding sequences. F: forward primer; R: reverse primer.

**Table 2:** Inhibition Rates of Antagonistic Fungi Against Kiwifruit Soft Rot Pathogens through Dual-Culture Plate Assay.

Antagonistic Fungi	Sources	Pathogen			
		*Fusarium*	*Alternaria*	*Botryos* *-phaeriaceae*	*Phomopsis*
N3-1-1	Soil	+++	+++	+++	++
Y5-1-1	Leaf	+++	+++	+++	++
Nd-1-2	Soil	+++	+++	+++	++
Pa-1-1	Bark	+	++	++	+
Pb-2-2	Bark	++++	++++	++++	++++
Pb-2-1	Bark	++	++	+	+
Nd-1-1	Soil	++++	++++	+++	++++
N2-1-1	Soil	++++	++++	++++	++
Y1-2-1	Leaf	++	+++	+++	++
Ya-1-1	Leaf	+	++	+	-
Pb-3-2	Bark	++	++	+	+
Pb-3-1	Bark	++	++	++	+
N5-3-1	Soil	+++	+++	+++	+++
P4-1-2	Bark	++	++	++	++
Pb-1-1	Bark	+++	+++	+++	+++
N3-1-2	Soil	+++	+++	+++	+++
Y4-2-2	Leaf	++	++	++	++
Nc-2-2	Soil	+++	+++	++	++
Y4-1-1	Leaf	+++	+++	+++	+++
Na-1-1	Soil	+++	+++	++	++
P1-1-1	Bark	+++	+++	+++	+++
P4-1-2	Bark	++	++	++	++
Y12-1	Leaf	-	+	-	-
Y8-1	Leaf	+++	+++	+++	+++
Y10-1	Leaf	++	++	+	-
Y13-1	Leaf	++	++	+	-
Y14-1	Leaf	-	-	-	-
Y9-1	Leaf	++	+++	++	++
Y18-1	Leaf	++	++	+	++
P5-a	Bark	++	++	++	++
Y28-1	Leaf	+++	+++	++	+
Pa-C	Bark	++	++	+	-
Pc-b	Bark	+++	+++	+++	+++
Y25-1	Bark	+++	+++	+++	+++
Pa-b	Bark	++	+++	+	-
P5-b	Bark	+++	+++	++	++
Y27-2	Bark	++	+++	++	+

Note: The antagonistic effect is graded as follows: ++++ indicates >70% inhibition; +++ indicates 50–70% inhibition; ++ indicates 30–50% inhibition; + indicates 10–30% inhibition; and - indicates no antagonistic effect (<10% inhibition).

**Table 3:** Inhibition rates (%) of antagonistic strains against four phytopathogenic fungi under three biocontrol evaluation systems.

Pathogen	Dual Culture Assay			Spore Suspension Assay			Fermentation Filtrates		
	Nd-1-1	N2-1-1	Pb-2-2	Nd-1-1	N2-1-1	Pb-2-2	Nd-1-1	N2-1-1	Pb-2-2
*Fusarium*	78.23 ± 0.76 b	79.81 ± 0.56 a	80.90 ± 9.47 a	20.16 ± 3.65 a	1.67 ± 2.11 c	13.98 ± 2.49 b	45.73 ± 0.80 a	44.15 ± 0.97 a	34.42 ± 1.34 b
*Alternaria*	78.37 ± 0.40 c	80.29 ± 0.73 b	81.85 ± 0.51 a	37.58 ± 2.75 b	42.17 ± 0.81 a	15.84 ± 1.88 c	65.56 ± 0.91 b	61.63 ± 0.88 c	84.98 ± 0.64 a
*Botryosphaeriaceae*	78.61 ± 0.63 b	79.34 ± 0.66 ab	80.05 ± 0.61 a	48.44 ± 1.64 a	30.57 ± 0.31 b	12.45 ± 0.78 c	98.11 ± 0.03 a	98.11 ± 0.03 a	98.11 ± 0.03 a
*Phomopsis*	60.10 ± 0.48 b	49.78 ± 0.54 c	80.29 ± 0.64 a	38.08 ± 1.05 a	23.32 ± 0.42 c	27.45 ± 1.07 b	33.80 ± 1.69 b	97.57 ± 0.03 a	97.57 ± 0.03 a

Note: Data in the table are grouped according to three independent experimental methods (dual culture assay, spore suspension assay, and fermentation filtrate method), with each group derived from three replicate experiments and results expressed as mean ± standard deviation (*n* = 3). Different lowercase letters within the same column indicate significant differences at the *p* < 0.05 level according to Duncan’s new multiple range test.

## Data Availability

The original contributions presented in this study are included in the article and Appendix A. Further inquiries can be directed to the corresponding author.

## Appendix A

**Table A1:** ANOVA table for antibacterial activity of spore suspension.

Fungi	Source	SS	DF	MS	F (DFn, DFd)	*p* Value
*Alternaria*	Time × Treatment	113.3	6	18.89	F (6, 36) = 115.5	*p* < 0.0001
	Time	2102	2	1051	F (2, 36) = 6428	*p* < 0.0001
	Treatment	514.1	3	171.4	F (3, 36) = 1048	*p* < 0.0001
	Residual	5.885	36	0.1635		
*Fusarium*	Time × Treatment	38.75	6	6.459	F (6, 36) = 53.11	*p* < 0.0001
	Time	1304	2	651.9	F (2, 36) = 5361	*p* < 0.0001
	Treatment	88.39	3	29.46	F (3, 36) = 242.3	*p* < 0.0001
	Residual	4.378	36	0.1216		
*Botryosphaeriaceae*	Time × Treatment	26.6	6	4.434	F (6, 36) = 30.41	*p* < 0.0001
	Time	1536	2	767.8	F (2, 36) = 5265	*p* < 0.0001
	Treatment	1535	3	511.8	F (3, 36) = 3510	*p* < 0.0001
	Residual	5.25	36	0.1458		
*Phomopsis*	Time × Treatment	20.05	6	3.342	F (6, 36) = 36.46	*p* < 0.0001
	Time	1390	2	695.1	F (2, 36) = 7585	*p* < 0.0001
	Treatment	129	3	43.01	F (3, 36) = 469.3	*p* < 0.0001
	Residual	3.299	36	0.09164		

Note: A Two-way ANOVA was conducted with time as the within-subject factor and treatment as the between-subject factor. The main effects of time and treatment, as well as their interaction, were tested. The treatment factor consisted of five groups: Blank (no pathogen and no treatment), Pathogen (pathogen only), N-2-1-1, Nd-1-1, and Pb-2-2 (antagonistic fungi and pathogen). Each treatment group included three independent subjects, and each subject was measured at three time points. The degrees of freedom for each F-ratio are indicated in parentheses.

**Table A2:** In vivo Lesion Sizes of Pathogens Treated with Fermentation Broths from Antagonistic Fungi.

Fungi	Source	SS	DF	MS	F (DFn, DFd)	*p* Value
*Alternaria*	Time × Treatment	429.3	6	71.55	F (6, 36) = 232.7	*p* < 0.0001
	Time	68.87	2	34.44	F (2, 36) = 112.0	*p* < 0.0001
	Treatment	3030	3	1010	F (3, 36) = 3285	*p* < 0.0001
	Residual	11.07	36	0.3075		
*Fusarium*	Time × Treatment	61.44	6	10.24	F (6, 36) = 37.60	*p* < 0.0001
	Time	20.66	2	10.33	F (2, 36) = 37.93	*p* < 0.0001
	Treatment	675.9	3	225.3	F (3, 36) = 827.4	*p* < 0.0001
	Residual	9.804	36	0.2723		
*Botryosphaeriaceae*	Time × Treatment	1296	6	216	F (6, 36) = 632.9	*p* < 0.0001
	Time	961.8	2	480.9	F (2, 36) = 1409	*p* < 0.0001
	Treatment	2184	3	727.9	F (3, 36) = 2133	*p* < 0.0001
	Residual	12.29	36	0.3413		
*Phomopsis*	Time × Treatment	1175	6	195.8	F (6, 36) = 1947	*p* < 0.0001
	Time	314.6	2	157.3	F (2, 36) = 1563	*p* < 0.0001
	Treatment	679.8	3	226.6	F (3, 36) = 2253	*p* < 0.0001
	Residual	3.622	36	0.1006		

Note: A Two-way ANOVA was conducted with time as the within-subject factor and treatment as the between-subject factor. The main effects of time and treatment, as well as their interaction, were tested. The treatment factor consisted of five groups: Blank (no pathogen and no treatment), Pathogen (pathogen only), N-2-1-1, Nd-1-1, and Pb-2-2 (antagonistic fungi and pathogen). Each treatment group included three independent subjects, and each subject was measured at three time points. The degrees of freedom for each F-ratio are indicated in parentheses.

**Table A3:** ANOVA table for enzyme activity.

Enzyme	Fungi	Source	SS	DF	MS	F (DFn, DFd)	*p* Value
SOD	*Alternaria*	Time × Treatment	850.4	8	106.3	F (8, 30) = 20.03	*p* < 0.0001
		Time	3491	2	1746	F (2, 30) = 329.0	*p* < 0.0001
		Treatment	6055	4	1514	F (4, 30) = 285.3	*p* < 0.0001
		Residual	159.2	30	5.306		
	*Fusarium*	Time × Treatment	1077	8	134.6	F (8, 30) = 12.46	*p* < 0.0001
		Time	4317	2	2158	F (2, 30) = 199.8	*p* < 0.0001
		Treatment	5322	4	1331	F (4, 30) = 123.2	*p* < 0.0001
		Residual	324.1	30	10.8		
	*Botryosphaeriaceae*	Time × Treatment	1633	8	204.1	F (8, 30) = 30.81	*p* < 0.0001
		Time	4187	2	2094	F (2, 30) = 316.1	*p* < 0.0001
		Treatment	7555	4	1889	F (4, 30) = 285.2	*p* < 0.0001
		Residual	198.7	30	6.623		
	*Phomopsis*	Time × Treatment	1413	8	176.6	F (8, 20) = 34.09	*p* < 0.0001
		Time	4718	2	2359	F (1.827, 18.27) = 455.4	*p* < 0.0001
		Treatment	5690	4	1423	F (4, 10) = 242.7	*p* < 0.0001
		Residual	103.6	20	5.18		
CAT	*Alternaria*	Time × Treatment	697.7	8	87.21	F (8, 30) = 37.98	*p* < 0.0001
		Time	29.81	2	14.91	F (2, 30) = 6.490	*p* = 0.0045
		Treatment	358.1	4	89.54	F (4, 30) = 38.99	*p* < 0.0001
		Residual	68.9	30	2.297		
	*Fusarium*	Time × Treatment	706.9	8	88.36	F (8, 30) = 77.97	*p* < 0.0001
		Time	16.77	2	8.384	F (2, 30) = 7.397	*p* = 0.0024
		Treatment	190	4	47.49	F (4, 30) = 41.90	*p* < 0.0001
		Residual	34	30	1.133		
	*Botryosphaeriaceae*	Time × Treatment	812.4	8	101.6	F (8, 30) = 63.33	*p* < 0.0001
		Time	142.8	2	71.41	F (2, 30) = 44.54	*p* < 0.0001
		Treatment	271.4	4	67.85	F (4, 30) = 42.32	*p* < 0.0001
		Residual	48.1	30	1.603		
	*Phomopsis*	Time × Treatment	633.8	8	79.23	F (8, 30) = 23.40	*p* < 0.0001
		Time	10.18	2	5.092	F (2, 30) = 1.504	*p* = 0.2385
		Treatment	333.4	4	83.35	F (4, 30) = 24.62	*p* < 0.0001
		Residual	101.6	30	3.385		
APX	*Alternaria*	Time × Treatment	56.77	8	7.096	F (8, 30) = 13.99	*p* < 0.0001
		Time	414.6	2	207.3	F (2, 30) = 408.8	*p* < 0.0001
		Treatment	1235	4	308.7	F (4, 30) = 608.8	*p* < 0.0001
		Residual	15.21	30	0.5071		
	*Fusarium*	Time × Treatment	52.22	8	6.528	F (8, 30) = 6.641	*p* < 0.0001
		Time	346.1	2	173.1	F (2, 30) = 176.1	*p* < 0.0001
		Treatment	1202	4	300.6	F (4, 30) = 305.8	*p* < 0.0001
		Residual	29.49	30	0.9829		
	*Botryosphaeriaceae*	Time × Treatment	52.32	8	6.54	F (8, 30) = 4.999	*p* = 0.0005
		Time	328.3	2	164.1	F (2, 30) = 125.5	*p* < 0.0001
		Treatment	1250	4	312.5	F (4, 30) = 238.9	*p* < 0.0001
		Residual	39.25	30	1.308		
	*Phomopsis*	Time × Treatment	54.42	8	6.803	F (8, 30) = 6.873	*p* < 0.0001
		Time	416.5	2	208.2	F (2, 30) = 210.4	*p* < 0.0001
		Treatment	1387	4	346.6	F (4, 30) = 350.2	*p* < 0.0001
		Residual	29.69	30	0.9898		
PAL	*Alternaria*	Time × Treatment	678.6	8	84.83	F (8, 30) = 12.11	*p* < 0.0001
		Time	256.1	2	128.1	F (2, 30) = 18.28	*p* < 0.0001
		Treatment	1031	4	257.8	F (4, 30) = 36.81	*p* < 0.0001
		Residual	210.1	30	7.004		
	*Fusarium*	Time × Treatment	681.8	8	85.22	F (8, 30) = 22.14	*p* < 0.0001
		Time	1181	2	590.4	F (2, 30) = 153.4	*p* < 0.0001
		Treatment	1493	4	373.3	F (4, 30) = 96.97	*p* < 0.0001
		Residual	115.5	30	3.85		
	*Botryosphaeriaceae*	Time × Treatment	528	8	66	F (8, 30) = 16.29	*p* < 0.0001
		Time	1275	2	637.6	F (2, 30) = 157.3	*p* < 0.0001
		Treatment	1414	4	353.4	F (4, 30) = 87.22	*p* < 0.0001
		Residual	121.6	30	4.052		
	*Phomopsis*	Time × Treatment	824.7	8	103.1	F (8, 30) = 17.29	*p* < 0.0001
		Time	600.3	2	300.2	F (2, 30) = 50.34	*p* < 0.0001
		Treatment	558.6	4	139.6	F (4, 30) = 23.42	*p* < 0.0001
		Residual	178.9	30	5.963		

Note: A Two-way ANOVA was conducted with time as the within-subject factor and treatment as the between-subject factor. The main effects of time and treatment, as well as their interaction, were tested. The treatment factor consisted of five groups: Blank (no pathogen and no treatment), Pathogen (pathogen only), N-2-1-1, Nd-1-1, and Pb-2-2 (antagonistic fungi and pathogen). Each treatment group included three independent subjects, and each subject was measured at three time points. The degrees of freedom for each F-ratio are indicated in parentheses.

**Table A4:** Tukey’s multiple comparisons test for antibacterial activity of spore suspension.

Pathogen	Comparison	Mean Difference	95.00% CI	SE of Difference	q	*df*	Adjusted *p*
*Alternaria*	3: Nd-1-1 vs. 3: N2-1-1	−0.3112	−1.309 to 0.6866	0.2859	1.54	36.00	0.9933
	3: Nd-1-1 vs. 3: Pb-2-2	−1.335	−2.333 to −0.3371	0.2859	6.604	36.00	0.0021
	3: Nd-1-1 vs. 3: Control	−3.114	−4.112 to −2.116	0.2859	15.4	36.00	<0.0001
	3: Nd-1-1 vs. 5: Nd-1-1	−7.558	−8.555 to −6.560	0.2859	37.38	36.00	<0.0001
	3: Nd-1-1 vs. 5: N2-1-1	−6.364	−7.362 to −5.366	0.2859	31.48	36.00	<0.0001
	3: Nd-1-1 vs. 5: Pb-2-2	−12.37	−13.36 to −11.37	0.2859	61.17	36.00	<0.0001
	3: Nd-1-1 vs. 5: Control	−16.1	−17.09 to −15.10	0.2859	79.62	36.00	<0.0001
	3: Nd-1-1 vs. 7: Nd-1-1	−13.51	−14.50 to −12.51	0.2859	66.8	36.00	<0.0001
	3: Nd-1-1 vs. 7: N2-1-1	−12.21	−13.21 to −11.22	0.2859	60.42	36.00	<0.0001
	3: Nd-1-1 vs. 7: Pb-2-2	−19.57	−20.57 to −18.57	0.2859	96.81	36.00	<0.0001
	3: Nd-1-1 vs. 7: Control	−24.01	−25.01 to −23.01	0.2859	118.8	36.00	<0.0001
	3: N2-1-1 vs. 3: Pb-2-2	−1.024	−2.022 to −0.02587	0.2859	5.064	36.00	0.0401
	3: N2-1-1 vs. 3: Control	−2.803	−3.800 to −1.805	0.2859	13.86	36.00	<0.0001
	3: N2-1-1 vs. 5: Nd-1-1	−7.246	−8.244 to −6.248	0.2859	35.84	36.00	<0.0001
	3: N2-1-1 vs. 5: N2-1-1	−6.053	−7.050 to −5.055	0.2859	29.94	36.00	<0.0001
	3: N2-1-1 vs. 5: Pb-2-2	−12.06	−13.05 to −11.06	0.2859	59.63	36.00	<0.0001
	3: N2-1-1 vs. 5: Control	−15.79	−16.78 to −14.79	0.2859	78.08	36.00	<0.0001
	3: N2-1-1 vs. 7: Nd-1-1	−13.19	−14.19 to −12.20	0.2859	65.26	36.00	<0.0001
	3: N2-1-1 vs. 7: N2-1-1	−11.9	−12.90 to −10.90	0.2859	58.88	36.00	<0.0001
	3: N2-1-1 vs. 7: Pb-2-2	−19.26	−20.26 to −18.26	0.2859	95.27	36.00	<0.0001
	3: N2-1-1 vs. 7: Control	−23.7	−24.69 to −22.70	0.2859	117.2	36.00	<0.0001
	3: Pb-2-2 vs. 3: Control	−1.779	−2.777 to −0.7809	0.2859	8.799	36.00	<0.0001
	3: Pb-2-2 vs. 5: Nd-1-1	−6.223	−7.220 to −5.225	0.2859	30.78	36.00	<0.0001
	3: Pb-2-2 vs. 5: N2-1-1	−5.029	−6.027 to −4.031	0.2859	24.87	36.00	<0.0001
	3: Pb-2-2 vs. 5: Pb-2-2	−11.03	−12.03 to −10.03	0.2859	54.57	36.00	<0.0001
	3: Pb-2-2 vs. 5: Control	−14.76	−15.76 to −13.76	0.2859	73.02	36.00	<0.0001
	3: Pb-2-2 vs. 7: Nd-1-1	−12.17	−13.17 to −11.17	0.2859	60.2	36.00	<0.0001
	3: Pb-2-2 vs. 7: N2-1-1	−10.88	−11.88 to −9.881	0.2859	53.81	36.00	<0.0001
	3: Pb-2-2 vs. 7: Pb-2-2	−18.24	−19.23 to −17.24	0.2859	90.21	36.00	<0.0001
	3: Pb-2-2 vs. 7: Control	−22.67	−23.67 to −21.67	0.2859	112.2	36.00	<0.0001
	3: Control vs. 5: Nd-1-1	−4.444	−5.442 to −3.446	0.2859	21.98	36.00	<0.0001
	3: Control vs. 5: N2-1-1	−3.25	−4.248 to −2.252	0.2859	16.08	36.00	<0.0001
	3: Control vs. 5: Pb-2-2	−9.253	−10.25 to −8.255	0.2859	45.77	36.00	<0.0001
	3: Control vs. 5: Control	−12.98	−13.98 to −11.98	0.2859	64.22	36.00	<0.0001
	3: Control vs. 7: Nd-1-1	−10.39	−11.39 to −9.393	0.2859	51.4	36.00	<0.0001
	3: Control vs. 7: N2-1-1	−9.1	−10.10 to −8.102	0.2859	45.01	36.00	<0.0001
	3: Control vs. 7: Pb-2-2	−16.46	−17.46 to −15.46	0.2859	81.41	36.00	<0.0001
	3: Control vs. 7: Control	−20.89	−21.89 to −19.90	0.2859	103.4	36.00	<0.0001
	5: Nd-1-1 vs. 5: N2-1-1	1.194	0.1959 to 2.192	0.2859	5.905	36.00	0.0085
	5: Nd-1-1 vs. 5: Pb-2-2	−4.809	−5.807 to −3.811	0.2859	23.79	36.00	<0.0001
	5: Nd-1-1 vs. 5: Control	−8.539	−9.537 to −7.541	0.2859	42.24	36.00	<0.0001
	5: Nd-1-1 vs. 7: Nd-1-1	−5.948	−6.945 to −4.950	0.2859	29.42	36.00	<0.0001
	5: Nd-1-1 vs. 7: N2-1-1	−4.656	−5.654 to −3.658	0.2859	23.03	36.00	<0.0001
	5: Nd-1-1 vs. 7: Pb-2-2	−12.01	−13.01 to −11.02	0.2859	59.43	36.00	<0.0001
	5: Nd-1-1 vs. 7: Control	−16.45	−17.45 to −15.45	0.2859	81.37	36.00	<0.0001
	5: N2-1-1 vs. 5: Pb-2-2	−6.003	−7.000 to −5.005	0.2859	29.69	36.00	<0.0001
	5: N2-1-1 vs. 5: Control	−9.733	−10.73 to −8.735	0.2859	48.14	36.00	<0.0001
	5: N2-1-1 vs. 7: Nd-1-1	−7.141	−8.139 to −6.143	0.2859	35.32	36.00	<0.0001
	5: N2-1-1 vs. 7: N2-1-1	−5.85	−6.848 to −4.852	0.2859	28.94	36.00	<0.0001
	5: N2-1-1 vs. 7: Pb-2-2	−13.21	−14.21 to −12.21	0.2859	65.33	36.00	<0.0001
	5: N2-1-1 vs. 7: Control	−17.64	−18.64 to −16.65	0.2859	87.28	36.00	<0.0001
	5: Pb-2-2 vs. 5: Control	−3.73	−4.728 to −2.732	0.2859	18.45	36.00	<0.0001
	5: Pb-2-2 vs. 7: Nd-1-1	−1.139	−2.137 to −0.1409	0.2859	5.633	36.00	0.0143
	5: Pb-2-2 vs. 7: N2-1-1	0.1525	−0.8454 to 1.150	0.2859	0.7543	36.00	>0.9999
	5: Pb-2-2 vs. 7: Pb-2-2	−7.205	−8.203 to −6.207	0.2859	35.64	36.00	<0.0001
	5: Pb-2-2 vs. 7: Control	−11.64	−12.64 to −10.64	0.2859	57.58	36.00	<0.0001
	5: Control vs. 7: Nd-1-1	2.591	1.593 to 3.589	0.2859	12.82	36.00	<0.0001
	5: Control vs. 7: N2-1-1	3.883	2.885 to 4.880	0.2859	19.2	36.00	<0.0001
	5: Control vs. 7: Pb-2-2	−3.475	−4.473 to −2.477	0.2859	17.19	36.00	<0.0001
	5: Control vs. 7: Control	−7.911	−8.909 to −6.913	0.2859	39.13	36.00	<0.0001
	7: Nd-1-1 vs. 7: N2-1-1	1.291	0.2934 to 2.289	0.2859	6.387	36.00	0.0033
	7: Nd-1-1 vs. 7: Pb-2-2	−6.066	−7.064 to −5.068	0.2859	30.01	36.00	<0.0001
	7: Nd-1-1 vs. 7: Control	−10.5	−11.50 to −9.505	0.2859	51.95	36.00	<0.0001
	7: N2-1-1 vs. 7: Pb-2-2	−7.358	−8.355 to −6.360	0.2859	36.39	36.00	<0.0001
	7: N2-1-1 vs. 7: Control	−11.79	−12.79 to −10.80	0.2859	58.34	36.00	<0.0001
	7: Pb-2-2 vs. 7: Control	−4.436	−5.434 to −3.438	0.2859	21.94	36.00	<0.0001
	3: Nd-1-1 vs. 3: N2-1-1	−1.1	−1.961 to −0.2393	0.2466	6.309	36.00	0.0038
	3: Nd-1-1 vs. 3: Pb-2-2	−2.378	−3.238 to −1.517	0.2466	13.64	36.00	<0.0001
	3: Nd-1-1 vs. 3: Control	−3.771	−4.632 to −2.911	0.2466	21.63	36.00	<0.0001
	3: Nd-1-1 vs. 5: Nd-1-1	−9.048	−9.908 to −8.187	0.2466	51.89	36.00	<0.0001
*Fusarium*	3: Nd-1-1 vs. 5: N2-1-1	−7.549	−8.409 to −6.688	0.2466	43.29	36.00	<0.0001
	3: Nd-1-1 vs. 5: Pb-2-2	−9.964	−10.82 to −9.103	0.2466	57.14	36.00	<0.0001
	3: Nd-1-1 vs. 5: Control	−12.53	−13.39 to −11.67	0.2466	71.85	36.00	<0.0001
	3: Nd-1-1 vs. 7: Nd-1-1	−12.26	−13.12 to −11.40	0.2466	70.3	36.00	<0.0001
	3: Nd-1-1 vs. 7: N2-1-1	−15.85	−16.71 to −14.99	0.2466	90.9	36.00	<0.0001
	3: Nd-1-1 vs. 7: Pb-2-2	−13.46	−14.32 to −12.60	0.2466	77.18	36.00	<0.0001
	3: Nd-1-1 vs. 7: Control	−16.18	−17.04 to −15.32	0.2466	92.79	36.00	<0.0001
	3: N2-1-1 vs. 3: Pb-2-2	−1.278	−2.138 to −0.4168	0.2466	7.327	36.00	0.0005
	3: N2-1-1 vs. 3: Control	−2.671	−3.532 to −1.811	0.2466	15.32	36.00	<0.0001
	3: N2-1-1 vs. 5: Nd-1-1	−7.948	−8.808 to −7.087	0.2466	45.58	36.00	<0.0001
	3: N2-1-1 vs. 5: N2-1-1	−6.449	−7.309 to −5.588	0.2466	36.99	36.00	<0.0001
	3: N2-1-1 vs. 5: Pb-2-2	−8.864	−9.724 to −8.003	0.2466	50.84	36.00	<0.0001
	3: N2-1-1 vs. 5: Control	−11.43	−12.29 to −10.57	0.2466	65.54	36.00	<0.0001
	3: N2-1-1 vs. 7: Nd-1-1	−11.16	−12.02 to −10.30	0.2466	63.99	36.00	<0.0001
	3: N2-1-1 vs. 7: N2-1-1	−14.75	−15.61 to −13.89	0.2466	84.59	36.00	<0.0001
	3: N2-1-1 vs. 7: Pb-2-2	−12.36	−13.22 to −11.50	0.2466	70.87	36.00	<0.0001
	3: N2-1-1 vs. 7: Control	−15.08	−15.94 to −14.22	0.2466	86.48	36.00	<0.0001
	3: Pb-2-2 vs. 3: Control	−1.394	−2.254 to −0.5331	0.2466	7.993	36.00	0.0001
	3: Pb-2-2 vs. 5: Nd-1-1	−6.67	−7.531 to −5.809	0.2466	38.25	36.00	<0.0001
	3: Pb-2-2 vs. 5: N2-1-1	−5.171	−6.032 to −4.311	0.2466	29.66	36.00	<0.0001
	3: Pb-2-2 vs. 5: Pb-2-2	−7.586	−8.447 to −6.726	0.2466	43.51	36.00	<0.0001
	3: Pb-2-2 vs. 5: Control	−10.15	−11.01 to −9.289	0.2466	58.21	36.00	<0.0001
	3: Pb-2-2 vs. 7: Nd-1-1	−9.88	−10.74 to −9.019	0.2466	56.66	36.00	<0.0001
	3: Pb-2-2 vs. 7: N2-1-1	−13.47	−14.33 to −12.61	0.2466	77.27	36.00	<0.0001
	3: Pb-2-2 vs. 7: Pb-2-2	−11.08	−11.94 to −10.22	0.2466	63.55	36.00	<0.0001
	3: Pb-2-2 vs. 7: Control	−13.8	−14.66 to −12.94	0.2466	79.15	36.00	<0.0001
	3: Control vs. 5: Nd-1-1	−5.276	−6.137 to −4.416	0.2466	30.26	36.00	<0.0001
	3: Control vs. 5: N2-1-1	−3.778	−4.638 to −2.917	0.2466	21.66	36.00	<0.0001
	3: Control vs. 5: Pb-2-2	−6.193	−7.053 to −5.332	0.2466	35.52	36.00	<0.0001
	3: Control vs. 5: Control	−8.756	−9.617 to −7.896	0.2466	50.22	36.00	<0.0001
	3: Control vs. 7: Nd-1-1	−8.486	−9.347 to −7.626	0.2466	48.67	36.00	<0.0001
	3: Control vs. 7: N2-1-1	−12.08	−12.94 to −11.22	0.2466	69.27	36.00	<0.0001
	3: Control vs. 7: Pb-2-2	−9.686	−10.55 to −8.826	0.2466	55.55	36.00	<0.0001
	3: Control vs. 7: Control	−12.41	−13.27 to −11.55	0.2466	71.16	36.00	<0.0001
	5: Nd-1-1 vs. 5: N2-1-1	1.499	0.6381 to 2.359	0.2466	8.596	36.00	<0.0001
	5: Nd-1-1 vs. 5: Pb-2-2	−0.9163	−1.777 to −0.05559	0.2466	5.255	36.00	0.0286
	5: Nd-1-1 vs. 5: Control	−3.48	−4.341 to −2.619	0.2466	19.96	36.00	<0.0001
	5: Nd-1-1 vs. 7: Nd-1-1	−3.21	−4.071 to −2.349	0.2466	18.41	36.00	<0.0001
	5: Nd-1-1 vs. 7: N2-1-1	−6.803	−7.663 to −5.942	0.2466	39.01	36.00	<0.0001
	5: Nd-1-1 vs. 7: Pb-2-2	−4.41	−5.271 to −3.549	0.2466	25.29	36.00	<0.0001
	5: Nd-1-1 vs. 7: Control	−7.131	−7.992 to −6.271	0.2466	40.9	36.00	<0.0001
	5: N2-1-1 vs. 5: Pb-2-2	−2.415	−3.276 to −1.554	0.2466	13.85	36.00	<0.0001
	5: N2-1-1 vs. 5: Control	−4.979	−5.839 to −4.118	0.2466	28.55	36.00	<0.0001
	5: N2-1-1 vs. 7: Nd-1-1	−4.709	−5.569 to −3.848	0.2466	27.01	36.00	<0.0001
	5: N2-1-1 vs. 7: N2-1-1	−8.301	−9.162 to −7.441	0.2466	47.61	36.00	<0.0001
	5: N2-1-1 vs. 7: Pb-2-2	−5.909	−6.769 to −5.048	0.2466	33.89	36.00	<0.0001
	5: N2-1-1 vs. 7: Control	−8.63	−9.491 to −7.769	0.2466	49.5	36.00	<0.0001
	5: Pb-2-2 vs. 5: Control	−2.564	−3.424 to −1.703	0.2466	14.7	36.00	<0.0001
	5: Pb-2-2 vs. 7: Nd-1-1	−2.294	−3.154 to −1.433	0.2466	13.16	36.00	<0.0001
	5: Pb-2-2 vs. 7: N2-1-1	−5.886	−6.747 to −5.026	0.2466	33.76	36.00	<0.0001
	5: Pb-2-2 vs. 7: Pb-2-2	−3.494	−4.354 to −2.633	0.2466	20.04	36.00	<0.0001
	5: Pb-2-2 vs. 7: Control	−6.215	−7.076 to −5.354	0.2466	35.64	36.00	<0.0001
	5: Control vs. 7: Nd-1-1	0.27	−0.5907 to 1.131	0.2466	1.549	36.00	0.993
	5: Control vs. 7: N2-1-1	−3.323	−4.183 to −2.462	0.2466	19.06	36.00	<0.0001
	5: Control vs. 7: Pb-2-2	−0.93	−1.791 to −0.06934	0.2466	5.334	36.00	0.0248
	5: Control vs. 7: Control	−3.651	−4.512 to −2.791	0.2466	20.94	36.00	<0.0001
	7: Nd-1-1 vs. 7: N2-1-1	−3.593	−4.453 to −2.732	0.2466	20.6	36.00	<0.0001
	7: Nd-1-1 vs. 7: Pb-2-2	−1.2	−2.061 to −0.3393	0.2466	6.882	36.00	0.0012
	7: Nd-1-1 vs. 7: Control	−3.921	−4.782 to −3.061	0.2466	22.49	36.00	<0.0001
	7: N2-1-1 vs. 7: Pb-2-2	2.393	1.532 to 3.253	0.2466	13.72	36.00	<0.0001
*Botryosphaeriaceae*	7: N2-1-1 vs. 7: Control	−0.3287	−1.189 to 0.5319	0.2466	1.885	36.00	0.9687
	7: Pb-2-2 vs. 7: Control	−2.721	−3.582 to −1.861	0.2466	15.61	36.00	<0.0001
	3: Nd-1-1 vs. 3: N2-1-1	−2.335	−3.277 to −1.393	0.2700	12.23	36.00	<0.0001
	3: Nd-1-1 vs. 3: Pb-2-2	−9.565	−10.51 to −8.623	0.2700	50.09	36.00	<0.0001
	3: Nd-1-1 vs. 3: Control	−15.02	−15.96 to −14.08	0.2700	78.65	36.00	<0.0001
	3: Nd-1-1 vs. 5: Nd-1-1	−6.804	−7.746 to −5.861	0.2700	35.63	36.00	<0.0001
	3: Nd-1-1 vs. 5: N2-1-1	−12.14	−13.08 to −11.19	0.2700	63.56	36.00	<0.0001
	3: Nd-1-1 vs. 5: Pb-2-2	−17.11	−18.05 to −16.17	0.2700	89.62	36.00	<0.0001
	3: Nd-1-1 vs. 5: Control	−23.2	−24.14 to −22.26	0.2700	121.5	36.00	<0.0001
	3: Nd-1-1 vs. 7: Nd-1-1	−13.46	−14.40 to −12.52	0.2700	70.5	36.00	<0.0001
	3: Nd-1-1 vs. 7: N2-1-1	−18.39	−19.33 to −17.45	0.2700	96.33	36.00	<0.0001
	3: Nd-1-1 vs. 7: Pb-2-2	−23.39	−24.33 to −22.45	0.2700	122.5	36.00	<0.0001
	3: Nd-1-1 vs. 7: Control	−26.82	−27.76 to −25.88	0.2700	140.5	36.00	<0.0001
	3: N2-1-1 vs. 3: Pb-2-2	−7.23	−8.172 to −6.288	0.2700	37.87	36.00	<0.0001
	3: N2-1-1 vs. 3: Control	−12.68	−13.62 to −11.74	0.2700	66.42	36.00	<0.0001
	3: N2-1-1 vs. 5: Nd-1-1	−4.469	−5.411 to −3.526	0.2700	23.4	36.00	<0.0001
	3: N2-1-1 vs. 5: N2-1-1	−9.801	−10.74 to −8.859	0.2700	51.33	36.00	<0.0001
	3: N2-1-1 vs. 5: Pb-2-2	−14.78	−15.72 to −13.83	0.2700	77.39	36.00	<0.0001
	3: N2-1-1 vs. 5: Control	−20.86	−21.80 to −19.92	0.2700	109.3	36.00	<0.0001
	3: N2-1-1 vs. 7: Nd-1-1	−11.13	−12.07 to −10.18	0.2700	58.27	36.00	<0.0001
	3: N2-1-1 vs. 7: N2-1-1	−16.06	−17.00 to −15.12	0.2700	84.1	36.00	<0.0001
	3: N2-1-1 vs. 7: Pb-2-2	−21.05	−22.00 to −20.11	0.2700	110.3	36.00	<0.0001
	3: N2-1-1 vs. 7: Control	−24.49	−25.43 to −23.54	0.2700	128.2	36.00	<0.0001
	3: Pb-2-2 vs. 3: Control	−5.453	−6.395 to −4.510	0.2700	28.56	36.00	<0.0001
	3: Pb-2-2 vs. 5: Nd-1-1	2.761	1.819 to 3.704	0.2700	14.46	36.00	<0.0001
	3: Pb-2-2 vs. 5: N2-1-1	−2.571	−3.514 to −1.629	0.2700	13.47	36.00	<0.0001
	3: Pb-2-2 vs. 5: Pb-2-2	−7.546	−8.489 to −6.604	0.2700	39.52	36.00	<0.0001
	3: Pb-2-2 vs. 5: Control	−13.63	−14.57 to −12.69	0.2700	71.4	36.00	<0.0001
	3: Pb-2-2 vs. 7: Nd-1-1	−3.896	−4.839 to −2.954	0.2700	20.41	36.00	<0.0001
	3: Pb-2-2 vs. 7: N2-1-1	−8.828	−9.770 to −7.885	0.2700	46.23	36.00	<0.0001
	3: Pb-2-2 vs. 7: Pb-2-2	−13.82	−14.77 to −12.88	0.2700	72.4	36.00	<0.0001
	3: Pb-2-2 vs. 7: Control	−17.26	−18.20 to −16.31	0.2700	90.38	36.00	<0.0001
	3: Control vs. 5: Nd-1-1	8.214	7.271 to 9.156	0.2700	43.02	36.00	<0.0001
	3: Control vs. 5: N2-1-1	2.881	1.939 to 3.824	0.2700	15.09	36.00	<0.0001
	3: Control vs. 5: Pb-2-2	−2.094	−3.036 to −1.151	0.2700	10.97	36.00	<0.0001
	3: Control vs. 5: Control	−8.18	−9.122 to −7.238	0.2700	42.84	36.00	<0.0001
	3: Control vs. 7: Nd-1-1	1.556	0.6138 to 2.499	0.2700	8.151	36.00	<0.0001
	3: Control vs. 7: N2-1-1	−3.375	−4.317 to −2.433	0.2700	17.68	36.00	<0.0001
	3: Control vs. 7: Pb-2-2	−8.371	−9.314 to −7.429	0.2700	43.84	36.00	<0.0001
	3: Control vs. 7: Control	−11.8	−12.75 to −10.86	0.2700	61.82	36.00	<0.0001
	5: Nd-1-1 vs. 5: N2-1-1	−5.333	−6.275 to −4.390	0.2700	27.93	36.00	<0.0001
	5: Nd-1-1 vs. 5: Pb-2-2	−10.31	−11.25 to −9.365	0.2700	53.98	36.00	<0.0001
	5: Nd-1-1 vs. 5: Control	−16.39	−17.34 to −15.45	0.2700	85.86	36.00	<0.0001
	5: Nd-1-1 vs. 7: Nd-1-1	−6.658	−7.600 to −5.715	0.2700	34.87	36.00	<0.0001
	5: Nd-1-1 vs. 7: N2-1-1	−11.59	−12.53 to −10.65	0.2700	60.69	36.00	<0.0001
	5: Nd-1-1 vs. 7: Pb-2-2	−16.59	−17.53 to −15.64	0.2700	86.86	36.00	<0.0001
	5: Nd-1-1 vs. 7: Control	−20.02	−20.96 to −19.08	0.2700	104.8	36.00	<0.0001
	5: N2-1-1 vs. 5: Pb-2-2	−4.975	−5.917 to −4.033	0.2700	26.06	36.00	<0.0001
	5: N2-1-1 vs. 5: Control	−11.06	−12.00 to −10.12	0.2700	57.93	36.00	<0.0001
	5: N2-1-1 vs. 7: Nd-1-1	−1.325	−2.267 to −0.3825	0.2700	6.939	36.00	0.0011
	5: N2-1-1 vs. 7: N2-1-1	−6.256	−7.199 to −5.314	0.2700	32.77	36.00	<0.0001
	5: N2-1-1 vs. 7: Pb-2-2	−11.25	−12.19 to −10.31	0.2700	58.93	36.00	<0.0001
	5: N2-1-1 vs. 7: Control	−14.69	−15.63 to −13.74	0.2700	76.91	36.00	<0.0001
	5: Pb-2-2 vs. 5: Control	−6.086	−7.029 to −5.144	0.2700	31.88	36.00	<0.0001
	5: Pb-2-2 vs. 7: Nd-1-1	3.65	2.708 to 4.592	0.2700	19.12	36.00	<0.0001
	5: Pb-2-2 vs. 7: N2-1-1	−1.281	−2.224 to −0.3388	0.2700	6.71	36.00	0.0017
	5: Pb-2-2 vs. 7: Pb-2-2	−6.278	−7.220 to −5.335	0.2700	32.88	36.00	<0.0001
	5: Pb-2-2 vs. 7: Control	−9.71	−10.65 to −8.768	0.2700	50.85	36.00	<0.0001
	5: Control vs. 7: Nd-1-1	9.736	8.794 to 10.68	0.2700	50.99	36.00	<0.0001
	5: Control vs. 7: N2-1-1	4.805	3.863 to 5.747	0.2700	25.17	36.00	<0.0001
	5: Control vs. 7: Pb-2-2	−0.1913	−1.134 to 0.7512	0.2700	1.002	36.00	0.9999
	5: Control vs. 7: Control	−3.624	−4.566 to −2.681	0.2700	18.98	36.00	<0.0001
	7: Nd-1-1 vs. 7: N2-1-1	−4.931	−5.874 to −3.989	0.2700	25.83	36.00	<0.0001
	7: Nd-1-1 vs. 7: Pb-2-2	−9.928	−10.87 to −8.985	0.2700	51.99	36.00	<0.0001
	7: Nd-1-1 vs. 7: Control	−13.36	−14.30 to −12.42	0.2700	69.97	36.00	<0.0001
	7: N2-1-1 vs. 7: Pb-2-2	−4.996	−5.939 to −4.054	0.2700	26.17	36.00	<0.0001
	7: N2-1-1 vs. 7: Control	−8.429	−9.371 to −7.486	0.2700	44.14	36.00	<0.0001
	7: Pb-2-2 vs. 7: Control	−3.433	−4.375 to −2.490	0.2700	17.98	36.00	<0.0001
	3: Nd-1-1 vs. 3: N2-1-1	−1.123	−1.870 to −0.3754	0.2141	7.416	36.00	0.0004
	3: Nd-1-1 vs. 3: Pb-2-2	−3.024	−3.771 to −2.277	0.2141	19.98	36.00	<0.0001
	3: Nd-1-1 vs. 3: Control	−3.931	−4.678 to −3.184	0.2141	25.97	36.00	<0.0001
	3: Nd-1-1 vs. 5: Nd-1-1	−7.823	−8.570 to −7.075	0.2141	51.68	36.00	<0.0001
	3: Nd-1-1 vs. 5: N2-1-1	−8.441	−9.188 to −7.694	0.2141	55.77	36.00	<0.0001
	3: Nd-1-1 vs. 5: Pb-2-2	−10.04	−10.79 to −9.294	0.2141	66.34	36.00	<0.0001
	3: Nd-1-1 vs. 5: Control	−13.06	−13.80 to −12.31	0.2141	86.26	36.00	<0.0001
	3: Nd-1-1 vs. 7: Nd-1-1	−12.47	−13.21 to −11.72	0.2141	82.35	36.00	<0.0001
	3: Nd-1-1 vs. 7: N2-1-1	−16.02	−16.76 to −15.27	0.2141	105.8	36.00	<0.0001
	3: N2-1-1 vs. 5: N2-1-1	−7.319	−8.066 to −6.572	0.2141	48.35	36.00	<0.0001
	3: N2-1-1 vs. 5: Pb-2-2	−8.919	−9.666 to −8.172	0.2141	58.92	36.00	<0.0001
	3: N2-1-1 vs. 5: Control	−11.93	−12.68 to −11.19	0.2141	78.84	36.00	<0.0001
	3: N2-1-1 vs. 7: Nd-1-1	−11.34	−12.09 to −10.60	0.2141	74.94	36.00	<0.0001
*Phomopsis*	3: N2-1-1 vs. 7: N2-1-1	−14.89	−15.64 to −14.15	0.2141	98.39	36.00	<0.0001
	3: N2-1-1 vs. 7: Pb-2-2	−13.9	−14.65 to −13.15	0.2141	91.83	36.00	<0.0001
	3: N2-1-1 vs. 7: Control	−15.86	−16.60 to −15.11	0.2141	104.7	36.00	<0.0001
	3: Pb-2-2 vs. 3: Control	−0.9075	−1.655 to −0.1604	0.2141	5.996	36.00	0.0071
	3: Pb-2-2 vs. 5: Nd-1-1	−4.799	−5.546 to −4.052	0.2141	31.7	36.00	<0.0001
	3: Pb-2-2 vs. 5: N2-1-1	−5.418	−6.165 to −4.670	0.2141	35.79	36.00	<0.0001
	3: Pb-2-2 vs. 5: Pb-2-2	−7.018	−7.765 to −6.270	0.2141	46.36	36.00	<0.0001
	3: Pb-2-2 vs. 5: Control	−10.03	−10.78 to −9.285	0.2141	66.28	36.00	<0.0001
	3: Pb-2-2 vs. 7: Nd-1-1	−9.441	−10.19 to −8.694	0.2141	62.38	36.00	<0.0001
	3: Pb-2-2 vs. 7: N2-1-1	−12.99	−13.74 to −12.24	0.2141	85.83	36.00	<0.0001
	3: Pb-2-2 vs. 7: Pb-2-2	−12	−12.74 to −11.25	0.2141	79.26	36.00	<0.0001
	3: Pb-2-2 vs. 7: Control	−13.95	−14.70 to −13.21	0.2141	92.19	36.00	<0.0001
	3: Control vs. 5: Nd-1-1	−3.891	−4.638 to −3.144	0.2141	25.71	36.00	<0.0001
	3: Control vs. 5: N2-1-1	−4.51	−5.257 to −3.763	0.2141	29.8	36.00	<0.0001
	3: Control vs. 5: Pb-2-2	−6.11	−6.857 to −5.363	0.2141	40.37	36.00	<0.0001
	3: Control vs. 5: Control	−9.125	−9.872 to −8.378	0.2141	60.29	36.00	<0.0001
	3: Control vs. 7: Nd-1-1	−8.534	−9.281 to −7.787	0.2141	56.38	36.00	<0.0001
	3: Control vs. 7: N2-1-1	−12.08	−12.83 to −11.34	0.2141	79.83	36.00	<0.0001
	3: Control vs. 7: Pb-2-2	−11.09	−11.84 to −10.34	0.2141	73.27	36.00	<0.0001
	3: Control vs. 7: Control	−13.05	−13.79 to −12.30	0.2141	86.19	36.00	<0.0001
	5: Nd-1-1 vs. 5: N2-1-1	−0.6187	−1.366 to 0.1284	0.2141	4.088	36.00	0.187
	5: Nd-1-1 vs. 5: Pb-2-2	−2.219	−2.966 to −1.472	0.2141	14.66	36.00	<0.0001
	5: Nd-1-1 vs. 5: Control	−5.234	−5.981 to −4.487	0.2141	34.58	36.00	<0.0001
	5: Nd-1-1 vs. 7: Nd-1-1	−4.643	−5.390 to −3.895	0.2141	30.67	36.00	<0.0001
	5: Nd-1-1 vs. 7: N2-1-1	−8.193	−8.940 to −7.445	0.2141	54.13	36.00	<0.0001
	5: Nd-1-1 vs. 7: Pb-2-2	−7.199	−7.946 to −6.452	0.2141	47.56	36.00	<0.0001
	5: Nd-1-1 vs. 7: Control	−9.155	−9.902 to −8.408	0.2141	60.48	36.00	<0.0001
	5: N2-1-1 vs. 5: Pb-2-2	−1.6	−2.347 to −0.8529	0.2141	10.57	36.00	<0.0001
	5: N2-1-1 vs. 5: Control	−4.615	−5.362 to −3.868	0.2141	30.49	36.00	<0.0001
	5: N2-1-1 vs. 7: Nd-1-1	−4.024	−4.771 to −3.277	0.2141	26.58	36.00	<0.0001
	5: N2-1-1 vs. 7: N2-1-1	−7.574	−8.321 to −6.827	0.2141	50.04	36.00	<0.0001
	5: N2-1-1 vs. 7: Pb-2-2	−6.58	−7.327 to −5.833	0.2141	43.47	36.00	<0.0001
	5: N2-1-1 vs. 7: Control	−8.536	−9.283 to −7.789	0.2141	56.4	36.00	<0.0001
	5: Pb-2-2 vs. 5: Control	−3.015	−3.762 to −2.268	0.2141	19.92	36.00	<0.0001
	5: Pb-2-2 vs. 7: Nd-1-1	−2.424	−3.171 to −1.677	0.2141	16.01	36.00	<0.0001
	5: Pb-2-2 vs. 7: N2-1-1	−5.974	−6.721 to −5.227	0.2141	39.47	36.00	<0.0001
	5: Pb-2-2 vs. 7: Pb-2-2	−4.98	−5.727 to −4.233	0.2141	32.9	36.00	<0.0001
	5: Pb-2-2 vs. 7: Control	−6.936	−7.683 to −6.189	0.2141	45.83	36.00	<0.0001
	5: Control vs. 7: Nd-1-1	0.5913	−0.1559 to 1.338	0.2141	3.906	36.00	0.2386
	5: Control vs. 7: N2-1-1	−2.959	−3.706 to −2.212	0.2141	19.55	36.00	<0.0001
	5: Control vs. 7: Pb-2-2	−1.965	−2.712 to −1.218	0.2141	12.98	36.00	<0.0001
	5: Control vs. 7: Control	−3.921	−4.668 to −3.174	0.2141	25.91	36.00	<0.0001
	7: Nd-1-1 vs. 7: N2-1-1	−3.55	−4.297 to −2.803	0.2141	23.45	36.00	<0.0001
	7: Nd-1-1 vs. 7: Pb-2-2	−2.556	−3.303 to −1.809	0.2141	16.89	36.00	<0.0001
	7: Nd-1-1 vs. 7: Control	−4.513	−5.260 to −3.765	0.2141	29.81	36.00	<0.0001
	7: N2-1-1 vs. 7: Pb-2-2	0.9937	0.2466 to 1.741	0.2141	6.565	36.00	0.0023
	7: N2-1-1 vs. 7: Control	−0.9625	−1.710 to −0.2154	0.2141	6.359	36.00	0.0035
	7: Pb-2-2 vs. 7: Control	−1.956	−2.703 to −1.209	0.2141	12.92	36.00	<0.0001

Note: Tukey’s multiple comparisons test was performed to compare the differences in antifungal activity among different treatment groups (Nd-1-1, N2-1-1, Pb-2-2, and Control) at 3, 5, and 7 days post-inoculation (dpi) against *Alternaria*, *Fusarium*, *Botryosphaeriaceae*, and *Phomopsis* pathogens. Mean difference represents the difference in mean values between the two groups in the comparison. A 95.00% CI indicates the 95% confidence interval for the mean difference (lower bound to upper bound). SE of difference refers to the standard error of the mean difference. The q statistic is the test statistic for Tukey’s test, and df denotes the error degrees of freedom. Adjusted *p* represents the two-sided *p* value adjusted for multiple comparisons to control the family-wise error rate (α = 0.05). All treatment groups showed significantly higher antifungal activity than the Control group at all time points (Adjusted *p* < 0.0001).

**Table A5:** Tukey’s multiple comparisons test for antagonistic activity of antagonistic Fungi fermentation broth.

Pathogen	Comparison	Mean Difference	95.00% CI	SE of Difference	q	*df*	Adjusted *p*
*Alternaria*	SDB: Nd-1-1 vs. SDB: N2-1-1	1.84	0.4715 to 3.209	0.3921	6.637	36.00	0.002
	SDB: Nd-1-1 vs. SDB: Pb-2-2	13.91	12.54 to 15.28	0.3921	50.18	36.00	<0.0001
	SDB: Nd-1-1 vs. SDB: Control	−11.56	−12.93 to −10.19	0.3921	41.7	36.00	<0.0001
	SDB: Nd-1-1 vs. PDB: Nd-1-1	7.928	6.559 to 9.296	0.3921	28.59	36.00	<0.0001
	SDB: Nd-1-1 vs. PDB: N2-1-1	6.909	5.540 to 8.277	0.3921	24.92	36.00	<0.0001
	SDB: Nd-1-1 vs. PDB: Pb-2-2	10.95	9.584 to 12.32	0.3921	39.5	36.00	<0.0001
	SDB: Nd-1-1 vs. PDB: Control	−11.56	−12.93 to −10.19	0.3921	41.7	36.00	<0.0001
	SDB: Nd-1-1 vs. MMM: Nd-1-1	8.094	6.725 to 9.462	0.3921	29.19	36.00	<0.0001
	SDB: Nd-1-1 vs. MMM: N2-1-1	4.804	3.435 to 6.172	0.3921	17.33	36.00	<0.0001
	SDB: Nd-1-1 vs. MMM: Pb-2-2	2.604	1.235 to 3.972	0.3921	9.391	36.00	<0.0001
	SDB: Nd-1-1 vs. MMM: Control	−11.56	−12.93 to −10.19	0.3921	41.7	36.00	<0.0001
	SDB: N2-1-1 vs. SDB: Pb-2-2	12.07	10.70 to 13.44	0.3921	43.54	36.00	<0.0001
	SDB: N2-1-1 vs. SDB: Control	−13.4	−14.77 to −12.03	0.3921	48.33	36.00	<0.0001
	SDB: N2-1-1 vs. PDB: Nd-1-1	6.088	4.719 to 7.456	0.3921	21.96	36.00	<0.0001
	SDB: N2-1-1 vs. PDB: N2-1-1	5.069	3.700 to 6.437	0.3921	18.28	36.00	<0.0001
	SDB: N2-1-1 vs. PDB: Pb-2-2	9.113	7.744 to 10.48	0.3921	32.87	36.00	<0.0001
	SDB: N2-1-1 vs. PDB: Control	−13.4	−14.77 to −12.03	0.3921	48.33	36.00	<0.0001
	SDB: N2-1-1 vs. MMM: Nd-1-1	6.254	4.885 to 7.622	0.3921	22.56	36.00	<0.0001
	SDB: N2-1-1 vs. MMM: N2-1-1	2.964	1.595 to 4.332	0.3921	10.69	36.00	<0.0001
	SDB: N2-1-1 vs. MMM: Pb-2-2	0.7638	−0.6048 to 2.132	0.3921	2.755	36.00	0.7228
	SDB: N2-1-1 vs. MMM: Control	−13.4	−14.77 to −12.03	0.3921	48.33	36.00	<0.0001
	SDB: Pb-2-2 vs. SDB: Control	−25.47	−26.84 to −24.10	0.3921	91.88	36.00	<0.0001
	SDB: Pb-2-2 vs. PDB: Nd-1-1	−5.985	−7.354 to −4.616	0.3921	21.59	36.00	<0.0001
	SDB: Nd-1-1 vs. SDB: N2-1-1	1.84	0.4715 to 3.209	0.3921	6.637	36.00	0.002
	SDB: Nd-1-1 vs. SDB: Pb-2-2	13.91	12.54 to 15.28	0.3921	50.18	36.00	<0.0001
	SDB: Nd-1-1 vs. SDB: Control	−11.56	−12.93 to −10.19	0.3921	41.7	36.00	<0.0001
	SDB: Nd-1-1 vs. PDB: Nd-1-1	7.928	6.559 to 9.296	0.3921	28.59	36.00	<0.0001
	SDB: Nd-1-1 vs. PDB: N2-1-1	6.909	5.540 to 8.277	0.3921	24.92	36.00	<0.0001
	SDB: Nd-1-1 vs. PDB: Pb-2-2	10.95	9.584 to 12.32	0.3921	39.5	36.00	<0.0001
	SDB: Nd-1-1 vs. PDB: Control	−11.56	−12.93 to −10.19	0.3921	41.7	36.00	<0.0001
	SDB: Nd-1-1 vs. MMM: Nd-1-1	8.094	6.725 to 9.462	0.3921	29.19	36.00	<0.0001
	SDB: Nd-1-1 vs. MMM: N2-1-1	4.804	3.435 to 6.172	0.3921	17.33	36.00	<0.0001
	SDB: Nd-1-1 vs. MMM: Pb-2-2	2.604	1.235 to 3.972	0.3921	9.391	36.00	<0.0001
	SDB: Nd-1-1 vs. MMM: Control	−11.56	−12.93 to −10.19	0.3921	41.7	36.00	<0.0001
	SDB: N2-1-1 vs. SDB: Pb-2-2	12.07	10.70 to 13.44	0.3921	43.54	36.00	<0.0001
	SDB: N2-1-1 vs. SDB: Control	−13.4	−14.77 to −12.03	0.3921	48.33	36.00	<0.0001
	SDB: N2-1-1 vs. PDB: Nd-1-1	6.088	4.719 to 7.456	0.3921	21.96	36.00	<0.0001
	SDB: N2-1-1 vs. PDB: N2-1-1	5.069	3.700 to 6.437	0.3921	18.28	36.00	<0.0001
	SDB: N2-1-1 vs. PDB: Pb-2-2	9.113	7.744 to 10.48	0.3921	32.87	36.00	<0.0001
	SDB: N2-1-1 vs. PDB: Control	−13.4	−14.77 to −12.03	0.3921	48.33	36.00	<0.0001
	SDB: N2-1-1 vs. MMM: Nd-1-1	6.254	4.885 to 7.622	0.3921	22.56	36.00	<0.0001
	SDB: N2-1-1 vs. MMM: N2-1-1	2.964	1.595 to 4.332	0.3921	10.69	36.00	<0.0001
	SDB: N2-1-1 vs. MMM: Pb-2-2	0.7638	−0.6048 to 2.132	0.3921	2.755	36.00	0.7228
	SDB: N2-1-1 vs. MMM: Control	−13.4	−14.77 to −12.03	0.3921	48.33	36.00	<0.0001
	SDB: Pb-2-2 vs. SDB: Control	−25.47	−26.84 to −24.10	0.3921	91.88	36.00	<0.0001
	SDB: Pb-2-2 vs. PDB: Nd-1-1	−5.985	−7.354 to −4.616	0.3921	21.59	36.00	<0.0001
	SDB: Pb-2-2 vs. PDB: N2-1-1	−7.004	−8.372 to −5.635	0.3921	25.26	36.00	<0.0001
	SDB: Pb-2-2 vs. PDB: Pb-2-2	−2.96	−4.329 to −1.591	0.3921	10.68	36.00	<0.0001
	SDB: Pb-2-2 vs. PDB: Control	−25.47	−26.84 to −24.10	0.3921	91.88	36.00	<0.0001
	SDB: Pb-2-2 vs. MMM: Nd-1-1	−5.819	−7.187 to −4.450	0.3921	20.99	36.00	<0.0001
	SDB: Pb-2-2 vs. MMM: N2-1-1	−9.109	−10.48 to −7.740	0.3921	32.85	36.00	<0.0001
	SDB: Pb-2-2 vs. MMM: Pb-2-2	−11.31	−12.68 to −9.940	0.3921	40.79	36.00	<0.0001
	SDB: Pb-2-2 vs. MMM: Control	−25.47	−26.84 to −24.10	0.3921	91.88	36.00	<0.0001
	SDB: Control vs. PDB: Nd-1-1	19.49	18.12 to 20.86	0.3921	70.29	36.00	<0.0001
	SDB: Control vs. PDB: N2-1-1	18.47	17.10 to 19.84	0.3921	66.61	36.00	<0.0001
	SDB: Control vs. PDB: Pb-2-2	22.51	21.14 to 23.88	0.3921	81.2	36.00	<0.0001
	SDB: Control vs. PDB: Control	0	−1.369 to 1.369	0.3921	0	36.00	>0.9999
	SDB: Control vs. MMM: Nd-1-1	19.65	18.29 to 21.02	0.3921	70.89	36.00	<0.0001
	SDB: Control vs. MMM: N2-1-1	16.36	15.00 to 17.73	0.3921	59.02	36.00	<0.0001
	SDB: Control vs. MMM: Pb-2-2	14.16	12.80 to 15.53	0.3921	51.09	36.00	<0.0001
	SDB: Control vs. MMM: Control	0	−1.369 to 1.369	0.3921	0	36.00	>0.9999
	PDB: Nd-1-1 vs. PDB: N2-1-1	−1.019	−2.387 to 0.3498	0.3921	3.674	36.00	0.3176
	PDB: Nd-1-1 vs. PDB: Pb-2-2	3.025	1.656 to 4.394	0.3921	10.91	36.00	<0.0001
	PDB: Nd-1-1 vs. PDB: Control	−19.49	−20.86 to −18.12	0.3921	70.29	36.00	<0.0001
	PDB: Nd-1-1 vs. MMM: Nd-1-1	0.1663	−1.202 to 1.535	0.3921	0.5996	36.00	>0.9999
	PDB: Nd-1-1 vs. MMM: N2-1-1	−3.124	−4.492 to −1.755	0.3921	11.27	36.00	<0.0001
	PDB: Nd-1-1 vs. MMM: Pb-2-2	−5.324	−6.692 to −3.955	0.3921	19.2	36.00	<0.0001
	PDB: Nd-1-1 vs. MMM: Control	−19.49	−20.86 to −18.12	0.3921	70.29	36.00	<0.0001
	PDB: N2-1-1 vs. PDB: Pb-2-2	4.044	2.675 to 5.412	0.3921	14.59	36.00	<0.0001
	PDB: N2-1-1 vs. PDB: Control	−18.47	−19.84 to −17.10	0.3921	66.61	36.00	<0.0001
	PDB: N2-1-1 vs. MMM: Nd-1-1	1.185	−0.1835 to 2.554	0.3921	4.274	36.00	0.1435
	PDB: N2-1-1 vs. MMM: N2-1-1	−2.105	−3.474 to −0.7365	0.3921	7.592	36.00	0.0003
	PDB: N2-1-1 vs. MMM: Pb-2-2	−4.305	−5.674 to −2.936	0.3921	15.53	36.00	<0.0001
	PDB: N2-1-1 vs. MMM: Control	−18.47	−19.84 to −17.10	0.3921	66.61	36.00	<0.0001
	PDB: Pb-2-2 vs. PDB: Control	−22.51	−23.88 to −21.14	0.3921	81.2	36.00	<0.0001
	PDB: Pb-2-2 vs. MMM: Nd-1-1	−2.859	−4.227 to −1.490	0.3921	10.31	36.00	<0.0001
	PDB: Pb-2-2 vs. MMM: N2-1-1	−6.149	−7.517 to −4.780	0.3921	22.18	36.00	<0.0001
	PDB: Pb-2-2 vs. MMM: Pb-2-2	−8.349	−9.717 to −6.980	0.3921	30.11	36.00	<0.0001
*Fusarium*	PDB: Pb-2-2 vs. MMM: Control	−22.51	−23.88 to −21.14	0.3921	81.2	36.00	<0.0001
	PDB: Control vs. MMM: Nd-1-1	19.65	18.29 to 21.02	0.3921	70.89	36.00	<0.0001
	PDB: Control vs. MMM: N2-1-1	16.36	15.00 to 17.73	0.3921	59.02	36.00	<0.0001
	PDB: Control vs. MMM: Pb-2-2	14.16	12.80 to 15.53	0.3921	51.09	36.00	<0.0001
	PDB: Control vs. MMM: Control	0	−1.369 to 1.369	0.3921	0	36.00	>0.9999
	MMM: Nd-1-1 vs. MMM: N2-1-1	−3.29	−4.659 to −1.921	0.3921	11.87	36.00	<0.0001
	MMM: Nd-1-1 vs. MMM: Pb-2-2	−5.49	−6.859 to −4.121	0.3921	19.8	36.00	<0.0001
	MMM: Nd-1-1 vs. MMM: Control	−19.65	−21.02 to −18.29	0.3921	70.89	36.00	<0.0001
	MMM: N2-1-1 vs. MMM: Pb-2-2	−2.2	−3.569 to −0.8315	0.3921	7.935	36.00	0.0001
	MMM: N2-1-1 vs. MMM: Control	−16.36	−17.73 to −15.00	0.3921	59.02	36.00	<0.0001
	MMM: Pb-2-2 vs. MMM: Control	−14.16	−15.53 to −12.80	0.3921	51.09	36.00	<0.0001
	SDB: Nd-1-1 vs. SDB: N2-1-1	0.3613	−0.9267 to 1.649	0.3690	1.385	36.00	0.9973
	SDB: Nd-1-1 vs. SDB: Pb-2-2	−2.721	−4.009 to −1.433	0.3690	10.43	36.00	<0.0001
	SDB: Nd-1-1 vs. SDB: Control	−10.19	−11.47 to −8.897	0.3690	39.03	36.00	<0.0001
	SDB: Nd-1-1 vs. PDB: Nd-1-1	−4.346	−5.634 to −3.058	0.3690	16.66	36.00	<0.0001
	SDB: Nd-1-1 vs. PDB: N2-1-1	−2.296	−3.584 to −1.008	0.3690	8.801	36.00	<0.0001
	SDB: Nd-1-1 vs. PDB: Pb-2-2	−1.966	−3.254 to −0.6783	0.3690	7.536	36.00	0.0003
	SDB: Nd-1-1 vs. PDB: Control	−10.19	−11.47 to −8.897	0.3690	39.03	36.00	<0.0001
	SDB: Nd-1-1 vs. MMM: Nd-1-1	0.7363	−0.5517 to 2.024	0.3690	2.822	36.00	0.6934
	SDB: Nd-1-1 vs. MMM: N2-1-1	−1.013	−2.300 to 0.2754	0.3690	3.88	36.00	0.2466
	SDB: Nd-1-1 vs. MMM: Pb-2-2	−3.903	−5.190 to −2.615	0.3690	14.96	36.00	<0.0001
	SDB: Nd-1-1 vs. MMM: Control	−10.19	−11.47 to −8.897	0.3690	39.03	36.00	<0.0001
	SDB: N2-1-1 vs. SDB: Pb-2-2	−3.083	−4.370 to −1.795	0.3690	11.81	36.00	<0.0001
	SDB: N2-1-1 vs. SDB: Control	−10.55	−11.83 to −9.258	0.3690	40.42	36.00	<0.0001
	SDB: N2-1-1 vs. PDB: Nd-1-1	−4.708	−5.995 to −3.420	0.3690	18.04	36.00	<0.0001
	SDB: N2-1-1 vs. PDB: N2-1-1	−2.658	−3.945 to −1.370	0.3690	10.19	36.00	<0.0001
	SDB: N2-1-1 vs. PDB: Pb-2-2	−2.328	−3.615 to −1.040	0.3690	8.92	36.00	<0.0001
	SDB: N2-1-1 vs. PDB: Control	−10.55	−11.83 to −9.258	0.3690	40.42	36.00	<0.0001
	SDB: N2-1-1 vs. MMM: Nd-1-1	0.375	−0.9129 to 1.663	0.3690	1.437	36.00	0.9963
	SDB: N2-1-1 vs. MMM: N2-1-1	−1.374	−2.662 to −0.08583	0.3690	5.265	36.00	0.0281
	SDB: N2-1-1 vs. MMM: Pb-2-2	−4.264	−5.552 to −2.976	0.3690	16.34	36.00	<0.0001
	SDB: N2-1-1 vs. MMM: Control	−10.55	−11.83 to −9.258	0.3690	40.42	36.00	<0.0001
	SDB: Pb-2-2 vs. SDB: Control	−7.464	−8.752 to −6.176	0.3690	28.61	36.00	<0.0001
	SDB: Pb-2-2 vs. PDB: Nd-1-1	−1.625	−2.913 to −0.3371	0.3690	6.228	36.00	0.0045
	SDB: Pb-2-2 vs. PDB: N2-1-1	0.425	−0.8629 to 1.713	0.3690	1.629	36.00	0.9895
	SDB: Pb-2-2 vs. PDB: Pb-2-2	0.755	−0.5329 to 2.043	0.3690	2.894	36.00	0.661
	SDB: Pb-2-2 vs. PDB: Control	−7.464	−8.752 to −6.176	0.3690	28.61	36.00	<0.0001
	SDB: Pb-2-2 vs. MMM: Nd-1-1	3.458	2.170 to 4.745	0.3690	13.25	36.00	<0.0001
	SDB: Pb-2-2 vs. MMM: N2-1-1	1.709	0.4208 to 2.997	0.3690	6.549	36.00	0.0024
	SDB: Pb-2-2 vs. MMM: Pb-2-2	−1.181	−2.469 to 0.1067	0.3690	4.527	36.00	0.0977
	SDB: Pb-2-2 vs. MMM: Control	−7.464	−8.752 to −6.176	0.3690	28.61	36.00	<0.0001
	SDB: Control vs. PDB: Nd-1-1	5.839	4.551 to 7.127	0.3690	22.38	36.00	<0.0001
	SDB: Control vs. PDB: N2-1-1	7.889	6.601 to 9.177	0.3690	30.23	36.00	<0.0001
	SDB: Control vs. PDB: Pb-2-2	8.219	6.931 to 9.507	0.3690	31.5	36.00	<0.0001
	SDB: Control vs. PDB: Control	0	−1.288 to 1.288	0.3690	0	36.00	>0.9999
	SDB: Control vs. MMM: Nd-1-1	10.92	9.633 to 12.21	0.3690	41.86	36.00	<0.0001
	SDB: Control vs. MMM: N2-1-1	9.173	7.885 to 10.46	0.3690	35.15	36.00	<0.0001
	SDB: Control vs. MMM: Pb-2-2	6.283	4.995 to 7.570	0.3690	24.08	36.00	<0.0001
	SDB: Control vs. MMM: Control	0	−1.288 to 1.288	0.3690	0	36.00	>0.9999
	PDB: Nd-1-1 vs. PDB: N2-1-1	2.05	0.7621 to 3.338	0.3690	7.857	36.00	0.0002
	PDB: Nd-1-1 vs. PDB: Pb-2-2	2.38	1.092 to 3.668	0.3690	9.122	36.00	<0.0001
	PDB: Nd-1-1 vs. PDB: Control	−5.839	−7.127 to −4.551	0.3690	22.38	36.00	<0.0001
	PDB: Nd-1-1 vs. MMM: Nd-1-1	5.083	3.795 to 6.370	0.3690	19.48	36.00	<0.0001
	PDB: Nd-1-1 vs. MMM: N2-1-1	3.334	2.046 to 4.622	0.3690	12.78	36.00	<0.0001
	PDB: Nd-1-1 vs. MMM: Pb-2-2	0.4438	−0.8442 to 1.732	0.3690	1.701	36.00	0.9854
	PDB: Nd-1-1 vs. MMM: Control	−5.839	−7.127 to −4.551	0.3690	22.38	36.00	<0.0001
	PDB: N2-1-1 vs. PDB: Pb-2-2	0.33	−0.9579 to 1.618	0.3690	1.265	36.00	0.9988
	PDB: N2-1-1 vs. PDB: Control	−7.889	−9.177 to −6.601	0.3690	30.23	36.00	<0.0001
	PDB: N2-1-1 vs. MMM: Nd-1-1	3.033	1.745 to 4.320	0.3690	11.62	36.00	<0.0001
	PDB: N2-1-1 vs. MMM: N2-1-1	1.284	−0.004173 to 2.572	0.3690	4.92	36.00	0.0514
	PDB: N2-1-1 vs. MMM: Pb-2-2	−1.606	−2.894 to −0.3183	0.3690	6.156	36.00	0.0052
	PDB: N2-1-1 vs. MMM: Control	−7.889	−9.177 to −6.601	0.3690	30.23	36.00	<0.0001
	PDB: Pb-2-2 vs. PDB: Control	−8.219	−9.507 to −6.931	0.3690	31.5	36.00	<0.0001
	PDB: Pb-2-2 vs. MMM: Nd-1-1	2.703	1.415 to 3.990	0.3690	10.36	36.00	<0.0001
	PDB: Pb-2-2 vs. MMM: N2-1-1	0.9537	−0.3342 to 2.242	0.3690	3.655	36.00	0.3247
	PDB: Pb-2-2 vs. MMM: Pb-2-2	−1.936	−3.224 to −0.6483	0.3690	7.421	36.00	0.0004
	PDB: Pb-2-2 vs. MMM: Control	−8.219	−9.507 to −6.931	0.3690	31.5	36.00	<0.0001
	PDB: Control vs. MMM: Nd-1-1	10.92	9.633 to 12.21	0.3690	41.86	36.00	<0.0001
	PDB: Control vs. MMM: N2-1-1	9.173	7.885 to 10.46	0.3690	35.15	36.00	<0.0001
	PDB: Control vs. MMM: Pb-2-2	6.283	4.995 to 7.570	0.3690	24.08	36.00	<0.0001
	PDB: Control vs. MMM: Control	0	−1.288 to 1.288	0.3690	0	36.00	>0.9999
	MMM: Nd-1-1 vs. MMM: N2-1-1	−1.749	−3.037 to −0.4608	0.3690	6.702	36.00	0.0017
	MMM: Nd-1-1 vs. MMM: Pb-2-2	−4.639	−5.927 to −3.351	0.3690	17.78	36.00	<0.0001
	MMM: Nd-1-1 vs. MMM: Control	−10.92	−12.21 to −9.633	0.3690	41.86	36.00	<0.0001
	MMM: N2-1-1 vs. MMM: Pb-2-2	−2.89	−4.178 to −1.602	0.3690	11.08	36.00	<0.0001
	MMM: N2-1-1 vs. MMM: Control	−9.173	−10.46 to −7.885	0.3690	35.15	36.00	<0.0001
	MMM: Pb-2-2 vs. MMM: Control	−6.283	−7.570 to −4.995	0.3690	24.08	36.00	<0.0001
	SDB: Nd-1-1 vs. SDB: N2-1-1	5.051	3.609 to 6.493	0.4131	17.29	36.00	<0.0001
*Botryosphaeriaceae*	SDB: Nd-1-1 vs. SDB: Pb-2-2	2.149	0.7069 to 3.591	0.4131	7.356	36.00	0.0004
	SDB: Nd-1-1 vs. SDB: Control	−5.055	−6.497 to −3.613	0.4131	17.31	36.00	<0.0001
	SDB: Nd-1-1 vs. PDB: Nd-1-1	21.01	19.57 to 22.45	0.4131	71.92	36.00	<0.0001
	SDB: Nd-1-1 vs. PDB: N2-1-1	7.953	6.511 to 9.394	0.4131	27.22	36.00	<0.0001
	SDB: Nd-1-1 vs. PDB: Pb-2-2	21.01	19.57 to 22.45	0.4131	71.92	36.00	<0.0001
	SDB: Nd-1-1 vs. PDB: Control	−5.055	−6.497 to −3.613	0.4131	17.31	36.00	<0.0001
	SDB: Nd-1-1 vs. MMM: Nd-1-1	10.08	8.639 to 11.52	0.4131	34.51	36.00	<0.0001
	SDB: Nd-1-1 vs. MMM: N2-1-1	21.01	19.57 to 22.45	0.4131	71.92	36.00	<0.0001
	SDB: Nd-1-1 vs. MMM: Pb-2-2	5.914	4.472 to 7.356	0.4131	20.25	36.00	<0.0001
	SDB: Nd-1-1 vs. MMM: Control	−5.055	−6.497 to −3.613	0.4131	17.31	36.00	<0.0001
	SDB: N2-1-1 vs. SDB: Pb-2-2	−2.903	−4.344 to −1.461	0.4131	9.937	36.00	<0.0001
	SDB: N2-1-1 vs. SDB: Control	−10.11	−11.55 to −8.664	0.4131	34.6	36.00	<0.0001
	SDB: N2-1-1 vs. PDB: Nd-1-1	15.96	14.51 to 17.40	0.4131	54.63	36.00	<0.0001
	SDB: N2-1-1 vs. PDB: N2-1-1	2.901	1.459 to 4.343	0.4131	9.932	36.00	<0.0001
	SDB: N2-1-1 vs. PDB: Pb-2-2	15.96	14.51 to 17.40	0.4131	54.63	36.00	<0.0001
	SDB: N2-1-1 vs. PDB: Control	−10.11	−11.55 to −8.664	0.4131	34.6	36.00	<0.0001
	SDB: N2-1-1 vs. MMM: Nd-1-1	5.03	3.588 to 6.472	0.4131	17.22	36.00	<0.0001
	SDB: N2-1-1 vs. MMM: N2-1-1	15.96	14.51 to 17.40	0.4131	54.63	36.00	<0.0001
	SDB: N2-1-1 vs. MMM: Pb-2-2	0.8625	−0.5793 to 2.304	0.4131	2.953	36.00	0.6338
	SDB: N2-1-1 vs. MMM: Control	−10.11	−11.55 to −8.664	0.4131	34.6	36.00	<0.0001
	SDB: Pb-2-2 vs. SDB: Control	−7.204	−8.646 to −5.762	0.4131	24.66	36.00	<0.0001
	SDB: Pb-2-2 vs. PDB: Nd-1-1	18.86	17.42 to 20.30	0.4131	64.56	36.00	<0.0001
	SDB: Pb-2-2 vs. PDB: N2-1-1	5.804	4.362 to 7.246	0.4131	19.87	36.00	<0.0001
	SDB: Pb-2-2 vs. PDB: Pb-2-2	18.86	17.42 to 20.30	0.4131	64.56	36.00	<0.0001
	SDB: Pb-2-2 vs. PDB: Control	−7.204	−8.646 to −5.762	0.4131	24.66	36.00	<0.0001
	SDB: Pb-2-2 vs. MMM: Nd-1-1	7.933	6.491 to 9.374	0.4131	27.16	36.00	<0.0001
	SDB: Pb-2-2 vs. MMM: N2-1-1	18.86	17.42 to 20.30	0.4131	64.56	36.00	<0.0001
	SDB: Pb-2-2 vs. MMM: Pb-2-2	3.765	2.323 to 5.207	0.4131	12.89	36.00	<0.0001
	SDB: Pb-2-2 vs. MMM: Control	−7.204	−8.646 to −5.762	0.4131	24.66	36.00	<0.0001
	SDB: Control vs. PDB: Nd-1-1	26.06	24.62 to 27.50	0.4131	89.22	36.00	<0.0001
	SDB: Control vs. PDB: N2-1-1	13.01	11.57 to 14.45	0.4131	44.53	36.00	<0.0001
	SDB: Control vs. PDB: Pb-2-2	26.06	24.62 to 27.50	0.4131	89.22	36.00	<0.0001
	SDB: Control vs. PDB: Control	0	−1.442 to 1.442	0.4131	0	36.00	>0.9999
	SDB: Control vs. MMM: Nd-1-1	15.14	13.69 to 16.58	0.4131	51.82	36.00	<0.0001
	SDB: Control vs. MMM: N2-1-1	26.06	24.62 to 27.50	0.4131	89.22	36.00	<0.0001
	SDB: Control vs. MMM: Pb-2-2	10.97	9.527 to 12.41	0.4131	37.55	36.00	<0.0001
	SDB: Control vs. MMM: Control	0	−1.442 to 1.442	0.4131	0	36.00	>0.9999
	PDB: Nd-1-1 vs. PDB: N2-1-1	−13.06	−14.50 to −11.61	0.4131	44.69	36.00	<0.0001
	PDB: Nd-1-1 vs. PDB: Pb-2-2	0	−1.442 to 1.442	0.4131	0	36.00	>0.9999
	PDB: Nd-1-1 vs. PDB: Control	−26.06	−27.50 to −24.62	0.4131	89.22	36.00	<0.0001
	PDB: Nd-1-1 vs. MMM: Nd-1-1	−10.93	−12.37 to −9.484	0.4131	37.41	36.00	<0.0001
	PDB: Nd-1-1 vs. MMM: N2-1-1	0	−1.442 to 1.442	0.4131	0	36.00	>0.9999
	PDB: Nd-1-1 vs. MMM: Pb-2-2	−15.09	−16.54 to −13.65	0.4131	51.67	36.00	<0.0001
	PDB: Nd-1-1 vs. MMM: Control	−26.06	−27.50 to −24.62	0.4131	89.22	36.00	<0.0001
	PDB: N2-1-1 vs. PDB: Pb-2-2	13.06	11.61 to 14.50	0.4131	44.69	36.00	<0.0001
	PDB: N2-1-1 vs. PDB: Control	−13.01	−14.45 to −11.57	0.4131	44.53	36.00	<0.0001
	PDB: N2-1-1 vs. MMM: Nd-1-1	2.129	0.6869 to 3.571	0.4131	7.288	36.00	0.0005
	PDB: N2-1-1 vs. MMM: N2-1-1	13.06	11.61 to 14.50	0.4131	44.69	36.00	<0.0001
	PDB: N2-1-1 vs. MMM: Pb-2-2	−2.039	−3.481 to −0.5969	0.4131	6.98	36.00	0.001
	PDB: N2-1-1 vs. MMM: Control	−13.01	−14.45 to −11.57	0.4131	44.53	36.00	<0.0001
	PDB: Pb-2-2 vs. PDB: Control	−26.06	−27.50 to −24.62	0.4131	89.22	36.00	<0.0001
	PDB: Pb-2-2 vs. MMM: Nd-1-1	−10.93	−12.37 to −9.484	0.4131	37.41	36.00	<0.0001
*Phomopsis*	PDB: Pb-2-2 vs. MMM: N2-1-1	0	−1.442 to 1.442	0.4131	0	36.00	>0.9999
	PDB: Pb-2-2 vs. MMM: Pb-2-2	−15.09	−16.54 to −13.65	0.4131	51.67	36.00	<0.0001
	PDB: Pb-2-2 vs. MMM: Control	−26.06	−27.50 to −24.62	0.4131	89.22	36.00	<0.0001
	PDB: Control vs. MMM: Nd-1-1	15.14	13.69 to 16.58	0.4131	51.82	36.00	<0.0001
	PDB: Control vs. MMM: N2-1-1	26.06	24.62 to 27.50	0.4131	89.22	36.00	<0.0001
	PDB: Control vs. MMM: Pb-2-2	10.97	9.527 to 12.41	0.4131	37.55	36.00	<0.0001
	PDB: Control vs. MMM: Control	0	−1.442 to 1.442	0.4131	0	36.00	>0.9999
	MMM: Nd-1-1 vs. MMM: N2-1-1	10.93	9.484 to 12.37	0.4131	37.41	36.00	<0.0001
	MMM: Nd-1-1 vs. MMM: Pb-2-2	−4.168	−5.609 to −2.726	0.4131	14.27	36.00	<0.0001
	MMM: Nd-1-1 vs. MMM: Control	−15.14	−16.58 to −13.69	0.4131	51.82	36.00	<0.0001
	MMM: N2-1-1 vs. MMM: Pb-2-2	−15.09	−16.54 to −13.65	0.4131	51.67	36.00	<0.0001
	MMM: N2-1-1 vs. MMM: Control	−26.06	−27.50 to −24.62	0.4131	89.22	36.00	<0.0001
	MMM: Pb-2-2 vs. MMM: Control	−10.97	−12.41 to −9.527	0.4131	37.55	36.00	<0.0001
	SDB: Nd-1-1 vs. SDB: N2-1-1	−2.034	−2.817 to −1.251	0.2243	12.82	36.00	<0.0001
	SDB: Nd-1-1 vs. SDB: Pb-2-2	13.17	12.39 to 13.96	0.2243	83.07	36.00	<0.0001
	SDB: Nd-1-1 vs. SDB: Control	−6.985	−7.768 to −6.202	0.2243	44.05	36.00	<0.0001
	SDB: Nd-1-1 vs. PDB: Nd-1-1	−2.701	−3.484 to −1.918	0.2243	17.03	36.00	<0.0001
	SDB: Nd-1-1 vs. PDB: N2-1-1	−3.913	−4.695 to −3.130	0.2243	24.67	36.00	<0.0001
	SDB: Nd-1-1 vs. PDB: Pb-2-2	−5.083	−5.865 to −4.300	0.2243	32.05	36.00	<0.0001
	SDB: Nd-1-1 vs. PDB: Control	−6.985	−7.768 to −6.202	0.2243	44.05	36.00	<0.0001
	SDB: Nd-1-1 vs. MMM: Nd-1-1	−2.273	−3.055 to −1.490	0.2243	14.33	36.00	<0.0001
	SDB: Nd-1-1 vs. MMM: N2-1-1	13.17	12.39 to 13.96	0.2243	83.07	36.00	<0.0001
	SDB: Nd-1-1 vs. MMM: Pb-2-2	−2.195	−2.978 to −1.412	0.2243	13.84	36.00	<0.0001
	SDB: Nd-1-1 vs. MMM: Control	−6.985	−7.768 to −6.202	0.2243	44.05	36.00	<0.0001
	SDB: N2-1-1 vs. SDB: Pb-2-2	15.21	14.42 to 15.99	0.2243	95.89	36.00	<0.0001
	SDB: N2-1-1 vs. SDB: Control	−4.951	−5.734 to −4.168	0.2243	31.22	36.00	<0.0001
	SDB: N2-1-1 vs. PDB: Nd-1-1	−0.6675	−1.450 to 0.1153	0.2243	4.209	36.00	0.1577
	SDB: N2-1-1 vs. PDB: N2-1-1	−1.879	−2.662 to −1.096	0.2243	11.85	36.00	<0.0001
	SDB: N2-1-1 vs. PDB: Pb-2-2	−3.049	−3.832 to −2.266	0.2243	19.22	36.00	<0.0001
	SDB: N2-1-1 vs. PDB: Control	−4.951	−5.734 to −4.168	0.2243	31.22	36.00	<0.0001
	SDB: N2-1-1 vs. MMM: Nd-1-1	−0.2388	−1.022 to 0.5440	0.2243	1.505	36.00	0.9945
	SDB: N2-1-1 vs. MMM: N2-1-1	15.21	14.42 to 15.99	0.2243	95.89	36.00	<0.0001
	SDB: N2-1-1 vs. MMM: Pb-2-2	−0.1613	−0.9440 to 0.6215	0.2243	1.017	36.00	0.9998
	SDB: N2-1-1 vs. MMM: Control	−4.951	−5.734 to −4.168	0.2243	31.22	36.00	<0.0001
	SDB: Pb-2-2 vs. SDB: Control	−20.16	−20.94 to −19.38	0.2243	127.1	36.00	<0.0001
	SDB: Pb-2-2 vs. PDB: Nd-1-1	−15.88	−16.66 to −15.09	0.2243	100.1	36.00	<0.0001
	SDB: Pb-2-2 vs. PDB: N2-1-1	−17.09	−17.87 to −16.30	0.2243	107.7	36.00	<0.0001
	SDB: Pb-2-2 vs. PDB: Pb-2-2	−18.26	−19.04 to −17.47	0.2243	115.1	36.00	<0.0001
	SDB: Pb-2-2 vs. PDB: Control	−20.16	−20.94 to −19.38	0.2243	127.1	36.00	<0.0001
	SDB: Pb-2-2 vs. MMM: Nd-1-1	−15.45	−16.23 to −14.66	0.2243	97.4	36.00	<0.0001
	SDB: Pb-2-2 vs. MMM: N2-1-1	0	−0.7828 to 0.7828	0.2243	0	36.00	>0.9999
	SDB: Pb-2-2 vs. MMM: Pb-2-2	−15.37	−16.15 to −14.59	0.2243	96.91	36.00	<0.0001
	SDB: Pb-2-2 vs. MMM: Control	−20.16	−20.94 to −19.38	0.2243	127.1	36.00	<0.0001
	SDB: Control vs. PDB: Nd-1-1	4.284	3.501 to 5.067	0.2243	27.01	36.00	<0.0001
	SDB: Control vs. PDB: N2-1-1	3.073	2.290 to 3.855	0.2243	19.37	36.00	<0.0001
	SDB: Control vs. PDB: Pb-2-2	1.903	1.120 to 2.685	0.2243	12	36.00	<0.0001
	SDB: Control vs. PDB: Control	0	−0.7828 to 0.7828	0.2243	0	36.00	>0.9999
	SDB: Control vs. MMM: Nd-1-1	4.713	3.930 to 5.495	0.2243	29.72	36.00	<0.0001
	SDB: Control vs. MMM: N2-1-1	20.16	19.38 to 20.94	0.2243	127.1	36.00	<0.0001
	SDB: Control vs. MMM: Pb-2-2	4.79	4.007 to 5.573	0.2243	30.2	36.00	<0.0001
	SDB: Control vs. MMM: Control	0	−0.7828 to 0.7828	0.2243	0	36.00	>0.9999
	PDB: Nd-1-1 vs. PDB: N2-1-1	−1.211	−1.994 to −0.4285	0.2243	7.638	36.00	0.0002
	PDB: Nd-1-1 vs. PDB: Pb-2-2	−2.381	−3.164 to −1.598	0.2243	15.02	36.00	<0.0001
	PDB: Nd-1-1 vs. PDB: Control	−4.284	−5.067 to −3.501	0.2243	27.01	36.00	<0.0001
	PDB: Nd-1-1 vs. MMM: Nd-1-1	0.4288	−0.3540 to 1.212	0.2243	2.704	36.00	0.7446
	PDB: Nd-1-1 vs. MMM: N2-1-1	15.88	15.09 to 16.66	0.2243	100.1	36.00	<0.0001
	PDB: Nd-1-1 vs. MMM: Pb-2-2	0.5063	−0.2765 to 1.289	0.2243	3.192	36.00	0.5224
	PDB: Nd-1-1 vs. MMM: Control	−4.284	−5.067 to −3.501	0.2243	27.01	36.00	<0.0001
	PDB: N2-1-1 vs. PDB: Pb-2-2	−1.17	−1.953 to −0.3872	0.2243	7.378	36.00	0.0004
	PDB: N2-1-1 vs. PDB: Control	−3.073	−3.855 to −2.290	0.2243	19.37	36.00	<0.0001
	PDB: N2-1-1 vs. MMM: Nd-1-1	1.64	0.8572 to 2.423	0.2243	10.34	36.00	<0.0001
	PDB: N2-1-1 vs. MMM: N2-1-1	17.09	16.30 to 17.87	0.2243	107.7	36.00	<0.0001
	PDB: N2-1-1 vs. MMM: Pb-2-2	1.718	0.9347 to 2.500	0.2243	10.83	36.00	<0.0001
	PDB: N2-1-1 vs. MMM: Control	−3.073	−3.855 to −2.290	0.2243	19.37	36.00	<0.0001
	PDB: Pb-2-2 vs. PDB: Control	−1.903	−2.685 to −1.120	0.2243	12	36.00	<0.0001
	PDB: Pb-2-2 vs. MMM: Nd-1-1	2.81	2.027 to 3.593	0.2243	17.72	36.00	<0.0001
	PDB: Pb-2-2 vs. MMM: N2-1-1	18.26	17.47 to 19.04	0.2243	115.1	36.00	<0.0001
	PDB: Pb-2-2 vs. MMM: Pb-2-2	2.888	2.105 to 3.670	0.2243	18.21	36.00	<0.0001
	PDB: Pb-2-2 vs. MMM: Control	−1.903	−2.685 to −1.120	0.2243	12	36.00	<0.0001
	PDB: Control vs. MMM: Nd-1-1	4.713	3.930 to 5.495	0.2243	29.72	36.00	<0.0001
	PDB: Control vs. MMM: N2-1-1	20.16	19.38 to 20.94	0.2243	127.1	36.00	<0.0001
	PDB: Control vs. MMM: Pb-2-2	4.79	4.007 to 5.573	0.2243	30.2	36.00	<0.0001
	PDB: Control vs. MMM: Control	0	−0.7828 to 0.7828	0.2243	0	36.00	>0.9999
	MMM: Nd-1-1 vs. MMM: N2-1-1	15.45	14.66 to 16.23	0.2243	97.4	36.00	<0.0001
	MMM: Nd-1-1 vs. MMM: Pb-2-2	0.0775	−0.7053 to 0.8603	0.2243	0.4887	36.00	>0.9999
	MMM: Nd-1-1 vs. MMM: Control	−4.713	−5.495 to −3.930	0.2243	29.72	36.00	<0.0001
	MMM: N2-1-1 vs. MMM: Pb-2-2	−15.37	−16.15 to −14.59	0.2243	96.91	36.00	<0.0001
	MMM: N2-1-1 vs. MMM: Control	−20.16	−20.94 to −19.38	0.2243	127.1	36.00	<0.0001
	MMM: Pb-2-2 vs. MMM: Control	−4.79	−5.573 to −4.007	0.2243	30.2	36.00	<0.0001

Note: Tukey’s multiple comparisons test was performed to compare the differences in antifungal activity of culture fermentation broth among treatment groups (Nd-1-1, N2-1-1, Pb-2-2, and Control) against *Alternaria*, *Fusarium*, *Botryosphaeriaceae*, and *Phomopsis* pathogens, across three culture media (SDB = Sabouraud Dextrose Broth; PDB = Potato Dextrose Broth; MMM = Martin-Modified Medium). Mean difference represents the difference in mean values between the two groups in the comparison. A 95.00% CI indicates the 95% confidence interval for the mean difference (lower bound to upper bound). SE of difference refers to the standard error of the mean difference. The q statistic is the test statistic for Tukey’s test, and df denotes the error degrees of freedom (df = 36 for all comparisons). Adjusted *p* represents the two-sided *p* value adjusted for multiple comparisons to control the family-wise error rate (α = 0.05). All treatment groups showed significantly higher antifungal activity than the Control group across all culture media (Adjusted *p* < 0.0001).

**Table A6:** Tukey’s multiple comparisons test for SOD enzyme activity.

Pathogen	Comparison	Mean Difference	95.00% CI	SE of Difference	q	*df*	Adjusted *p*
*Alternaria*	3: Blank vs. 3: Pathogen	17.14	10.21 to 24.07	1.881	12.89	30	<0.0001
	3: Blank vs. 3: N2-1-1	−4.487	−11.42 to 2.444	1.881	3.374	30	0.5421
	3: Blank vs. 3: Nd-1-1	−7.65	−14.58 to −0.7188	1.881	5.752	30	0.02
	3: Blank vs. 3: Pb-2-2	−6.969	−13.90 to −0.03797	1.881	5.24	30	0.0477
	3: Blank vs. 5: Blank	−12.44	−19.37 to −5.509	1.881	9.353	30	<0.0001
	3: Blank vs. 5: Pathogen	26.02	19.09 to 32.95	1.881	19.57	30	<0.0001
	3: Blank vs. 5: N2-1-1	2.276	−4.655 to 9.207	1.881	1.711	30	0.9949
	3: Blank vs. 5: Nd-1-1	−0.9239	−7.855 to 6.007	1.881	0.6947	30	>0.9999
	3: Blank vs. 5: Pb-2-2	−1.815	−8.746 to 5.116	1.881	1.365	30	0.9995
	3: Blank vs. 7: Blank	0.115	−6.816 to 7.046	1.881	0.08644	30	>0.9999
	3: Blank vs. 7: Pathogen	45.32	38.39 to 52.25	1.881	34.07	30	<0.0001
	3: Blank vs. 7: N2-1-1	21.24	14.31 to 28.17	1.881	15.97	30	<0.0001
	3: Blank vs. 7: Nd-1-1	15.07	8.142 to 22.00	1.881	11.33	30	<0.0001
	3: Blank vs. 7: Pb-2-2	16.34	9.410 to 23.27	1.881	12.29	30	<0.0001
	3: Pathogen vs. 3: N2-1-1	−21.63	−28.56 to −14.69	1.881	16.26	30	<0.0001
	3: Pathogen vs. 3: Nd-1-1	−24.79	−31.72 to −17.86	1.881	18.64	30	<0.0001
	3: Pathogen vs. 3: Pb-2-2	−24.11	−31.04 to −17.18	1.881	18.13	30	<0.0001
	3: Pathogen vs. 5: Blank	−29.58	−36.51 to −22.65	1.881	22.24	30	<0.0001
	3: Pathogen vs. 5: Pathogen	8.884	1.953 to 15.81	1.881	6.68	30	0.0037
	3: Pathogen vs. 5: N2-1-1	−14.86	−21.79 to −7.931	1.881	11.17	30	<0.0001
	3: Pathogen vs. 5: Nd-1-1	−18.06	−24.99 to −11.13	1.881	13.58	30	<0.0001
	3: Pathogen vs. 5: Pb-2-2	−18.95	−25.88 to −12.02	1.881	14.25	30	<0.0001
	3: Pathogen vs. 7: Blank	−17.02	−23.95 to −10.09	1.881	12.8	30	<0.0001
	3: Pathogen vs. 7: Pathogen	28.18	21.25 to 35.11	1.881	21.19	30	<0.0001
	3: Pathogen vs. 7: N2-1-1	4.101	−2.830 to 11.03	1.881	3.084	30	0.675
	3: Pathogen vs. 7: Nd-1-1	−2.065	−8.996 to 4.866	1.881	1.553	30	0.998
	3: Pathogen vs. 7: Pb-2-2	−0.797	−7.728 to 6.134	1.881	0.5993	30	>0.9999
	3: N2-1-1 vs. 3: Nd-1-1	−3.163	−10.09 to 3.768	1.881	2.378	30	0.9229
	3: N2-1-1 vs. 3: Pb-2-2	−2.482	−9.413 to 4.449	1.881	1.866	30	0.9886
	3: N2-1-1 vs. 5: Blank	−7.953	−14.88 to −1.022	1.881	5.98	30	0.0134
	3: N2-1-1 vs. 5: Pathogen	30.51	23.58 to 37.44	1.881	22.94	30	<0.0001
	3: N2-1-1 vs. 5: N2-1-1	6.763	−0.1677 to 13.69	1.881	5.085	30	0.0613
	3: N2-1-1 vs. 5: Nd-1-1	3.563	−3.368 to 10.49	1.881	2.679	30	0.838
	3: N2-1-1 vs. 5: Pb-2-2	2.672	−4.259 to 9.603	1.881	2.009	30	0.9785
	3: N2-1-1 vs. 7: Blank	4.602	−2.329 to 11.53	1.881	3.46	30	0.5027
	3: N2-1-1 vs. 7: Pathogen	49.8	42.87 to 56.73	1.881	37.45	30	<0.0001
	3: N2-1-1 vs. 7: N2-1-1	25.73	18.80 to 32.66	1.881	19.34	30	<0.0001
	3: N2-1-1 vs. 7: Nd-1-1	19.56	12.63 to 26.49	1.881	14.71	30	<0.0001
	3: N2-1-1 vs. 7: Pb-2-2	20.83	13.90 to 27.76	1.881	15.66	30	<0.0001
	3: Nd-1-1 vs. 3: Pb-2-2	0.6808	−6.250 to 7.612	1.881	0.5119	30	>0.9999
	3: Nd-1-1 vs. 5: Blank	−4.79	−11.72 to 2.141	1.881	3.602	30	0.4401
	3: Nd-1-1 vs. 5: Pathogen	33.67	26.74 to 40.60	1.881	25.32	30	<0.0001
	3: Nd-1-1 vs. 5: N2-1-1	9.926	2.995 to 16.86	1.881	7.463	30	0.0008
	3: Nd-1-1 vs. 5: Nd-1-1	6.726	−0.2051 to 13.66	1.881	5.057	30	0.0641
	3: Nd-1-1 vs. 5: Pb-2-2	5.835	−1.096 to 12.77	1.881	4.387	30	0.1735
	3: Nd-1-1 vs. 7: Blank	7.765	0.8337 to 14.70	1.881	5.838	30	0.0172
	3: Nd-1-1 vs. 7: Pathogen	52.97	46.04 to 59.90	1.881	39.83	30	<0.0001
	3: Nd-1-1 vs. 7: N2-1-1	28.89	21.96 to 35.82	1.881	21.72	30	<0.0001
	3: Nd-1-1 vs. 7: Nd-1-1	22.72	15.79 to 29.65	1.881	17.09	30	<0.0001
	3: Nd-1-1 vs. 7: Pb-2-2	23.99	17.06 to 30.92	1.881	18.04	30	<0.0001
	3: Pb-2-2 vs. 5: Blank	−5.471	−12.40 to 1.460	1.881	4.113	30	0.2481
	3: Pb-2-2 vs. 5: Pathogen	32.99	26.06 to 39.92	1.881	24.81	30	<0.0001
	3: Pb-2-2 vs. 5: N2-1-1	9.245	2.314 to 16.18	1.881	6.951	30	0.0022
	3: Pb-2-2 vs. 5: Nd-1-1	6.045	−0.8859 to 12.98	1.881	4.545	30	0.1391
	3: Pb-2-2 vs. 5: Pb-2-2	5.154	−1.777 to 12.08	1.881	3.875	30	0.3295
	3: Pb-2-2 vs. 7: Blank	7.084	0.1529 to 14.01	1.881	5.326	30	0.0414
	3: Pb-2-2 vs. 7: Pathogen	52.29	45.35 to 59.22	1.881	39.31	30	<0.0001
	3: Pb-2-2 vs. 7: N2-1-1	28.21	21.28 to 35.14	1.881	21.21	30	<0.0001
	3: Pb-2-2 vs. 7: Nd-1-1	22.04	15.11 to 28.97	1.881	16.57	30	<0.0001
	3: Pb-2-2 vs. 7: Pb-2-2	23.31	16.38 to 30.24	1.881	17.53	30	<0.0001
	5: Blank vs. 5: Pathogen	38.46	31.53 to 45.39	1.881	28.92	30	<0.0001
	5: Blank vs. 5: N2-1-1	14.72	7.785 to 21.65	1.881	11.06	30	<0.0001
	5: Blank vs. 5: Nd-1-1	11.52	4.585 to 18.45	1.881	8.659	30	<0.0001
	5: Blank vs. 5: Pb-2-2	10.62	3.694 to 17.56	1.881	7.989	30	0.0003
	5: Blank vs. 7: Blank	12.55	5.624 to 19.49	1.881	9.44	30	<0.0001
	5: Blank vs. 7: Pathogen	57.76	50.83 to 64.69	1.881	43.43	30	<0.0001
	5: Blank vs. 7: N2-1-1	33.68	26.75 to 40.61	1.881	25.32	30	<0.0001
	5: Blank vs. 7: Nd-1-1	27.51	20.58 to 34.44	1.881	20.69	30	<0.0001
	5: Blank vs. 7: Pb-2-2	28.78	21.85 to 35.71	1.881	21.64	30	<0.0001
	5: Pathogen vs. 5: N2-1-1	−23.75	−30.68 to −16.81	1.881	17.85	30	<0.0001
	5: Pathogen vs. 5: Nd-1-1	−26.95	−33.88 to −20.01	1.881	20.26	30	<0.0001
	5: Pathogen vs. 5: Pb-2-2	−27.84	−34.77 to −20.91	1.881	20.93	30	<0.0001
	5: Pathogen vs. 7: Blank	−25.91	−32.84 to −18.98	1.881	19.48	30	<0.0001
	5: Pathogen vs. 7: Pathogen	19.29	12.36 to 26.23	1.881	14.51	30	<0.0001
	5: Pathogen vs. 7: N2-1-1	−4.782	−11.71 to 2.149	1.881	3.596	30	0.4425
	5: Pathogen vs. 7: Nd-1-1	−10.95	−17.88 to −4.018	1.881	8.232	30	0.0002
	5: Pathogen vs. 7: Pb-2-2	−9.681	−16.61 to −2.750	1.881	7.279	30	0.0012
	5: N2-1-1 vs. 5: Nd-1-1	−3.2	−10.13 to 3.731	1.881	2.406	30	0.9166
	5: N2-1-1 vs. 5: Pb-2-2	−4.091	−11.02 to 2.840	1.881	3.076	30	0.6784
	5: N2-1-1 vs. 7: Blank	−2.161	−9.092 to 4.770	1.881	1.625	30	0.9969
	5: N2-1-1 vs. 7: Pathogen	43.04	36.11 to 49.97	1.881	32.36	30	<0.0001
	5: N2-1-1 vs. 7: N2-1-1	18.96	12.03 to 25.89	1.881	14.26	30	<0.0001
	5: N2-1-1 vs. 7: Nd-1-1	12.8	5.866 to 19.73	1.881	9.622	30	<0.0001
	5: N2-1-1 vs. 7: Pb-2-2	14.06	7.134 to 21.00	1.881	10.58	30	<0.0001
	5: Nd-1-1 vs. 5: Pb-2-2	−0.8913	−7.822 to 6.040	1.881	0.6702	30	>0.9999
	5: Nd-1-1 vs. 7: Blank	1.039	−5.892 to 7.970	1.881	0.7811	30	>0.9999
	5: Nd-1-1 vs. 7: Pathogen	46.24	39.31 to 53.17	1.881	34.77	30	<0.0001
	5: Nd-1-1 vs. 7: N2-1-1	22.16	15.23 to 29.09	1.881	16.66	30	<0.0001
	5: Nd-1-1 vs. 7: Nd-1-1	16	9.066 to 22.93	1.881	12.03	30	<0.0001
	5: Nd-1-1 vs. 7: Pb-2-2	17.27	10.33 to 24.20	1.881	12.98	30	<0.0001
	5: Pb-2-2 vs. 7: Blank	1.93	−5.001 to 8.861	1.881	1.451	30	0.999
	5: Pb-2-2 vs. 7: Pathogen	47.13	40.20 to 54.06	1.881	35.44	30	<0.0001
	5: Pb-2-2 vs. 7: N2-1-1	23.05	16.12 to 29.99	1.881	17.33	30	<0.0001
	5: Pb-2-2 vs. 7: Nd-1-1	16.89	9.957 to 23.82	1.881	12.7	30	<0.0001
	5: Pb-2-2 vs. 7: Pb-2-2	18.16	11.23 to 25.09	1.881	13.65	30	<0.0001
	7: Blank vs. 7: Pathogen	45.2	38.27 to 52.13	1.881	33.99	30	<0.0001
	7: Blank vs. 7: N2-1-1	21.12	14.19 to 28.06	1.881	15.88	30	<0.0001
	7: Blank vs. 7: Nd-1-1	14.96	8.027 to 21.89	1.881	11.25	30	<0.0001
*Fusarium*	7: Blank vs. 7: Pb-2-2	16.23	9.295 to 23.16	1.881	12.2	30	<0.0001
	7: Pathogen vs. 7: N2-1-1	−24.08	−31.01 to −17.15	1.881	18.1	30	<0.0001
	7: Pathogen vs. 7: Nd-1-1	−30.24	−37.17 to −23.31	1.881	22.74	30	<0.0001
	7: Pathogen vs. 7: Pb-2-2	−28.98	−35.91 to −22.04	1.881	21.79	30	<0.0001
	7: N2-1-1 vs. 7: Nd-1-1	−6.166	−13.10 to 0.7647	1.881	4.636	30	0.122
	7: N2-1-1 vs. 7: Pb-2-2	−4.898	−11.83 to 2.033	1.881	3.683	30	0.4055
	7: Nd-1-1 vs. 7: Pb-2-2	1.268	−5.663 to 8.199	1.881	0.9534	30	>0.9999
	3: Blank vs. 3: Pathogen	13.06	3.170 to 22.95	2.684	6.882	30	0.0025
	3: Blank vs. 3: N2-1-1	−9.941	−19.83 to −0.05146	2.684	5.239	30	0.0478
	3: Blank vs. 3: Nd-1-1	−10.32	−20.21 to −0.4348	2.684	5.44	30	0.0342
	3: Blank vs. 3: Pb-2-2	−10.25	−20.14 to −0.3629	2.684	5.403	30	0.0364
	3: Blank vs. 5: Blank	−12.44	−22.33 to −2.550	2.684	6.555	30	0.0047
	3: Blank vs. 5: Pathogen	22.63	12.74 to 32.52	2.684	11.92	30	<0.0001
	3: Blank vs. 5: N2-1-1	−1.432	−11.32 to 8.457	2.684	0.7547	30	>0.9999
	3: Blank vs. 5: Nd-1-1	−2.44	−12.33 to 7.449	2.684	1.286	30	0.9997
	3: Blank vs. 5: Pb-2-2	−0.293	−10.18 to 9.596	2.684	0.1544	30	>0.9999
	3: Blank vs. 7: Blank	0.115	−9.774 to 10.00	2.684	0.06058	30	>0.9999
	3: Blank vs. 7: Pathogen	44.98	35.09 to 54.87	2.684	23.7	30	<0.0001
	3: Blank vs. 7: N2-1-1	17.66	7.768 to 27.55	2.684	9.305	30	<0.0001
	3: Blank vs. 7: Nd-1-1	15.75	5.859 to 25.64	2.684	8.299	30	0.0002
	3: Blank vs. 7: Pb-2-2	17.66	7.769 to 27.55	2.684	9.305	30	<0.0001
	3: Pathogen vs. 3: N2-1-1	−23	−32.89 to −13.11	2.684	12.12	30	<0.0001
	3: Pathogen vs. 3: Nd-1-1	−23.38	−33.27 to −13.49	2.684	12.32	30	<0.0001
	3: Pathogen vs. 3: Pb-2-2	−23.31	−33.20 to −13.42	2.684	12.28	30	<0.0001
	3: Pathogen vs. 5: Blank	−25.5	−35.39 to −15.61	2.684	13.44	30	<0.0001
	3: Pathogen vs. 5: Pathogen	9.57	−0.3197 to 19.46	2.684	5.043	30	0.0656
	3: Pathogen vs. 5: N2-1-1	−14.49	−24.38 to −4.602	2.684	7.637	30	0.0006
	3: Pathogen vs. 5: Nd-1-1	−15.5	−25.39 to −5.610	2.684	8.168	30	0.0002
	3: Pathogen vs. 5: Pb-2-2	−13.35	−23.24 to −3.463	2.684	7.036	30	0.0019
	3: Pathogen vs. 7: Blank	−12.94	−22.83 to −3.055	2.684	6.821	30	0.0029
	3: Pathogen vs. 7: Pathogen	31.92	22.03 to 41.81	2.684	16.82	30	<0.0001
	3: Pathogen vs. 7: N2-1-1	4.598	−5.292 to 14.49	2.684	2.423	30	0.9126
	3: Pathogen vs. 7: Nd-1-1	2.689	−7.200 to 12.58	2.684	1.417	30	0.9992
	3: Pathogen vs. 7: Pb-2-2	4.599	−5.290 to 14.49	2.684	2.423	30	0.9125
	3: N2-1-1 vs. 3: Nd-1-1	−0.3833	−10.27 to 9.506	2.684	0.202	30	>0.9999
	3: N2-1-1 vs. 3: Pb-2-2	−0.3115	−10.20 to 9.578	2.684	0.1641	30	>0.9999
	3: N2-1-1 vs. 5: Blank	−2.499	−12.39 to 7.390	2.684	1.317	30	0.9997
	3: N2-1-1 vs. 5: Pathogen	32.57	22.68 to 42.46	2.684	17.16	30	<0.0001
	3: N2-1-1 vs. 5: N2-1-1	8.509	−1.381 to 18.40	2.684	4.484	30	0.1517
	3: N2-1-1 vs. 5: Nd-1-1	7.5	−2.389 to 17.39	2.684	3.953	30	0.3014
	3: N2-1-1 vs. 5: Pb-2-2	9.648	−0.2416 to 19.54	2.684	5.084	30	0.0614
	3: N2-1-1 vs. 7: Blank	10.06	0.1664 to 19.94	2.684	5.299	30	0.0433
	3: N2-1-1 vs. 7: Pathogen	54.92	45.03 to 64.81	2.684	28.94	30	<0.0001
	3: N2-1-1 vs. 7: N2-1-1	27.6	17.71 to 37.49	2.684	14.54	30	<0.0001
	3: N2-1-1 vs. 7: Nd-1-1	25.69	15.80 to 35.58	2.684	13.54	30	<0.0001
	3: N2-1-1 vs. 7: Pb-2-2	27.6	17.71 to 37.49	2.684	14.54	30	<0.0001
	3: Nd-1-1 vs. 3: Pb-2-2	0.07183	−9.817 to 9.961	2.684	0.03785	30	>0.9999
	3: Nd-1-1 vs. 5: Blank	−2.116	−12.00 to 7.774	2.684	1.115	30	>0.9999
	3: Nd-1-1 vs. 5: Pathogen	32.95	23.06 to 42.84	2.684	17.37	30	<0.0001
	3: Nd-1-1 vs. 5: N2-1-1	8.892	−0.9974 to 18.78	2.684	4.686	30	0.1134
	3: Nd-1-1 vs. 5: Nd-1-1	7.884	−2.006 to 17.77	2.684	4.155	30	0.2356
	3: Nd-1-1 vs. 5: Pb-2-2	10.03	0.1417 to 19.92	2.684	5.286	30	0.0442
	3: Nd-1-1 vs. 7: Blank	10.44	0.5497 to 20.33	2.684	5.501	30	0.0309
	3: Nd-1-1 vs. 7: Pathogen	55.3	45.41 to 65.19	2.684	29.14	30	<0.0001
	3: Nd-1-1 vs. 7: N2-1-1	27.98	18.09 to 37.87	2.684	14.75	30	<0.0001
	3: Nd-1-1 vs. 7: Nd-1-1	26.07	16.18 to 35.96	2.684	13.74	30	<0.0001
	3: Nd-1-1 vs. 7: Pb-2-2	27.98	18.09 to 37.87	2.684	14.75	30	<0.0001
	3: Pb-2-2 vs. 5: Blank	−2.188	−12.08 to 7.702	2.684	1.153	30	>0.9999
	3: Pb-2-2 vs. 5: Pathogen	32.88	22.99 to 42.77	2.684	17.33	30	<0.0001
	3: Pb-2-2 vs. 5: N2-1-1	8.82	−1.069 to 18.71	2.684	4.648	30	0.1199
	3: Pb-2-2 vs. 5: Nd-1-1	7.812	−2.077 to 17.70	2.684	4.117	30	0.2471
	3: Pb-2-2 vs. 5: Pb-2-2	9.959	0.06990 to 19.85	2.684	5.248	30	0.0471
	3: Pb-2-2 vs. 7: Blank	10.37	0.4779 to 20.26	2.684	5.463	30	0.0329
	3: Pb-2-2 vs. 7: Pathogen	55.23	45.34 to 65.12	2.684	29.1	30	<0.0001
	3: Pb-2-2 vs. 7: N2-1-1	27.91	18.02 to 37.80	2.684	14.71	30	<0.0001
	3: Pb-2-2 vs. 7: Nd-1-1	26	16.11 to 35.89	2.684	13.7	30	<0.0001
	3: Pb-2-2 vs. 7: Pb-2-2	27.91	18.02 to 37.80	2.684	14.71	30	<0.0001
	5: Blank vs. 5: Pathogen	35.07	25.18 to 44.96	2.684	18.48	30	<0.0001
	5: Blank vs. 5: N2-1-1	11.01	1.118 to 20.90	2.684	5.801	30	0.0184
	5: Blank vs. 5: Nd-1-1	9.999	0.1102 to 19.89	2.684	5.269	30	0.0455
	5: Blank vs. 5: Pb-2-2	12.15	2.257 to 22.04	2.684	6.401	30	0.0062
	5: Blank vs. 7: Blank	12.55	2.665 to 22.44	2.684	6.616	30	0.0042
	5: Blank vs. 7: Pathogen	57.42	47.53 to 67.31	2.684	30.26	30	<0.0001
	5: Blank vs. 7: N2-1-1	30.1	20.21 to 39.99	2.684	15.86	30	<0.0001
	5: Blank vs. 7: Nd-1-1	28.19	18.30 to 38.08	2.684	14.85	30	<0.0001
	5: Blank vs. 7: Pb-2-2	30.1	20.21 to 39.99	2.684	15.86	30	<0.0001
	5: Pathogen vs. 5: N2-1-1	−24.06	−33.95 to −14.17	2.684	12.68	30	<0.0001
	5: Pathogen vs. 5: Nd-1-1	−25.07	−34.96 to −15.18	2.684	13.21	30	<0.0001
	5: Pathogen vs. 5: Pb-2-2	−22.92	−32.81 to −13.03	2.684	12.08	30	<0.0001
	5: Pathogen vs. 7: Blank	−22.51	−32.40 to −12.62	2.684	11.86	30	<0.0001
	5: Pathogen vs. 7: Pathogen	22.35	12.46 to 32.24	2.684	11.78	30	<0.0001
	5: Pathogen vs. 7: N2-1-1	−4.972	−14.86 to 4.917	2.684	2.62	30	0.8575
	5: Pathogen vs. 7: Nd-1-1	−6.88	−16.77 to 3.009	2.684	3.626	30	0.4297
	5: Pathogen vs. 7: Pb-2-2	−4.971	−14.86 to 4.919	2.684	2.619	30	0.8578
	5: N2-1-1 vs. 5: Nd-1-1	−1.008	−10.90 to 8.881	2.684	0.5312	30	>0.9999
	5: N2-1-1 vs. 5: Pb-2-2	1.139	−8.750 to 11.03	2.684	0.6003	30	>0.9999
	5: N2-1-1 vs. 7: Blank	1.547	−8.342 to 11.44	2.684	0.8153	30	>0.9999
	5: N2-1-1 vs. 7: Pathogen	46.41	36.52 to 56.30	2.684	24.46	30	<0.0001
	5: N2-1-1 vs. 7: N2-1-1	19.09	9.200 to 28.98	2.684	10.06	30	<0.0001
	5: N2-1-1 vs. 7: Nd-1-1	17.18	7.291 to 27.07	2.684	9.054	30	<0.0001
	5: N2-1-1 vs. 7: Pb-2-2	19.09	9.201 to 28.98	2.684	10.06	30	<0.0001
	5: Nd-1-1 vs. 5: Pb-2-2	2.147	−7.742 to 12.04	2.684	1.132	30	>0.9999
	5: Nd-1-1 vs. 7: Blank	2.555	−7.334 to 12.44	2.684	1.347	30	0.9996
	5: Nd-1-1 vs. 7: Pathogen	47.42	37.53 to 57.31	2.684	24.99	30	<0.0001
	5: Nd-1-1 vs. 7: N2-1-1	20.1	10.21 to 29.99	2.684	10.59	30	<0.0001
	5: Nd-1-1 vs. 7: Nd-1-1	18.19	8.300 to 28.08	2.684	9.585	30	<0.0001
	5: Nd-1-1 vs. 7: Pb-2-2	20.1	10.21 to 29.99	2.684	10.59	30	<0.0001
	5: Pb-2-2 vs. 7: Blank	0.408	−9.481 to 10.30	2.684	0.215	30	>0.9999
	5: Pb-2-2 vs. 7: Pathogen	45.27	35.38 to 55.16	2.684	23.86	30	<0.0001
	5: Pb-2-2 vs. 7: N2-1-1	17.95	8.061 to 27.84	2.684	9.459	30	<0.0001
	5: Pb-2-2 vs. 7: Nd-1-1	16.04	6.152 to 25.93	2.684	8.453	30	0.0001
	5: Pb-2-2 vs. 7: Pb-2-2	17.95	8.062 to 27.84	2.684	9.46	30	<0.0001
	7: Blank vs. 7: Pathogen	44.86	34.97 to 54.75	2.684	23.64	30	<0.0001
	7: Blank vs. 7: N2-1-1	17.54	7.653 to 27.43	2.684	9.244	30	<0.0001
	7: Blank vs. 7: Nd-1-1	15.63	5.744 to 25.52	2.684	8.238	30	0.0002
	7: Blank vs. 7: Pb-2-2	17.54	7.654 to 27.43	2.684	9.245	30	<0.0001
	7: Pathogen vs. 7: N2-1-1	−27.32	−37.21 to −17.43	2.684	14.4	30	<0.0001
	7: Pathogen vs. 7: Nd-1-1	−29.23	−39.12 to −19.34	2.684	15.4	30	<0.0001
	7: Pathogen vs. 7: Pb-2-2	−27.32	−37.21 to −17.43	2.684	14.4	30	<0.0001
	7: N2-1-1 vs. 7: Nd-1-1	−1.908	−11.80 to 7.981	2.684	1.006	30	>0.9999
	7: N2-1-1 vs. 7: Pb-2-2	0.0013	−9.888 to 9.891	2.684	0.000685	30	>0.9999
	7: Nd-1-1 vs. 7: Pb-2-2	1.91	−7.979 to 11.80	2.684	1.006	30	>0.9999
	3: Blank vs. 3: Pathogen	18.95	11.21 to 26.70	2.101	12.76	30	<0.0001
	3: Blank vs. 3: N2-1-1	−3.049	−10.79 to 4.695	2.101	2.052	30	0.9744
	3: Blank vs. 3: Nd-1-1	−3.757	−11.50 to 3.987	2.101	2.528	30	0.8852
	3: Blank vs. 3: Pb-2-2	−4.229	−11.97 to 3.515	2.101	2.846	30	0.7762
	3: Blank vs. 5: Blank	−12.44	−20.18 to −4.696	2.101	8.372	30	0.0001
	3: Blank vs. 5: Pathogen	41.66	33.92 to 49.41	2.101	28.04	30	<0.0001
	3: Blank vs. 5: N2-1-1	17.65	9.905 to 25.39	2.101	11.88	30	<0.0001
	3: Blank vs. 5: Nd-1-1	16.41	8.663 to 24.15	2.101	11.04	30	<0.0001
*Botryosphaeriaceae*	3: Blank vs. 5: Pb-2-2	16.3	8.557 to 24.04	2.101	10.97	30	<0.0001
	3: Blank vs. 7: Blank	0.115	−7.628 to 7.858	2.101	0.07737	30	>0.9999
	3: Blank vs. 7: Pathogen	48.62	40.87 to 56.36	2.101	32.72	30	<0.0001
	3: Blank vs. 7: N2-1-1	29.43	21.69 to 37.18	2.101	19.81	30	<0.0001
	3: Blank vs. 7: Nd-1-1	24.63	16.89 to 32.38	2.101	16.58	30	<0.0001
	3: Blank vs. 7: Pb-2-2	22.3	14.55 to 30.04	2.101	15.01	30	<0.0001
	3: Pathogen vs. 3: N2-1-1	−22	−29.75 to −14.26	2.101	14.81	30	<0.0001
	3: Pathogen vs. 3: Nd-1-1	−22.71	−30.45 to −14.97	2.101	15.28	30	<0.0001
	3: Pathogen vs. 3: Pb-2-2	−23.18	−30.93 to −15.44	2.101	15.6	30	<0.0001
	3: Pathogen vs. 5: Blank	−31.39	−39.14 to −23.65	2.101	21.13	30	<0.0001
	3: Pathogen vs. 5: Pathogen	22.71	14.97 to 30.45	2.101	15.28	30	<0.0001
	3: Pathogen vs. 5: N2-1-1	−1.304	−9.048 to 6.439	2.101	0.8778	30	>0.9999
	3: Pathogen vs. 5: Nd-1-1	−2.547	−10.29 to 5.197	2.101	1.714	30	0.9948
	3: Pathogen vs. 5: Pb-2-2	−2.652	−10.40 to 5.091	2.101	1.785	30	0.9924
	3: Pathogen vs. 7: Blank	−18.84	−26.58 to −11.09	2.101	12.68	30	<0.0001
	3: Pathogen vs. 7: Pathogen	29.66	21.92 to 37.41	2.101	19.96	30	<0.0001
	3: Pathogen vs. 7: N2-1-1	10.48	2.736 to 18.22	2.101	7.053	30	0.0018
	3: Pathogen vs. 7: Nd-1-1	5.679	−2.065 to 13.42	2.101	3.822	30	0.3497
	3: Pathogen vs. 7: Pb-2-2	3.345	−4.398 to 11.09	2.101	2.251	30	0.9476
	3: N2-1-1 vs. 3: Nd-1-1	−0.7078	−8.451 to 7.036	2.101	0.4763	30	>0.9999
	3: N2-1-1 vs. 3: Pb-2-2	−1.18	−8.923 to 6.564	2.101	0.7941	30	>0.9999
	3: N2-1-1 vs. 5: Blank	−9.391	−17.13 to −1.647	2.101	6.32	30	0.0072
	3: N2-1-1 vs. 5: Pathogen	44.71	36.97 to 52.46	2.101	30.09	30	<0.0001
	3: N2-1-1 vs. 5: N2-1-1	20.7	12.95 to 28.44	2.101	13.93	30	<0.0001
	3: N2-1-1 vs. 5: Nd-1-1	19.46	11.71 to 27.20	2.101	13.09	30	<0.0001
	3: N2-1-1 vs. 5: Pb-2-2	19.35	11.61 to 27.09	2.101	13.02	30	<0.0001
	3: N2-1-1 vs. 7: Blank	3.164	−4.580 to 10.91	2.101	2.129	30	0.9656
	3: N2-1-1 vs. 7: Pathogen	51.67	43.92 to 59.41	2.101	34.77	30	<0.0001
	3: N2-1-1 vs. 7: N2-1-1	32.48	24.74 to 40.22	2.101	21.86	30	<0.0001
	3: N2-1-1 vs. 7: Nd-1-1	27.68	19.94 to 35.42	2.101	18.63	30	<0.0001
	3: N2-1-1 vs. 7: Pb-2-2	25.35	17.60 to 33.09	2.101	17.06	30	<0.0001
	3: Nd-1-1 vs. 3: Pb-2-2	−0.4721	−8.216 to 7.271	2.101	0.3178	30	>0.9999
	3: Nd-1-1 vs. 5: Blank	−8.683	−16.43 to −0.9397	2.101	5.844	30	0.017
	3: Nd-1-1 vs. 5: Pathogen	45.42	37.68 to 53.16	2.101	30.57	30	<0.0001
	3: Nd-1-1 vs. 5: N2-1-1	21.41	13.66 to 29.15	2.101	14.41	30	<0.0001
	3: Nd-1-1 vs. 5: Nd-1-1	20.16	12.42 to 27.91	2.101	13.57	30	<0.0001
	3: Nd-1-1 vs. 5: Pb-2-2	20.06	12.31 to 27.80	2.101	13.5	30	<0.0001
	3: Nd-1-1 vs. 7: Blank	3.872	−3.872 to 11.62	2.101	2.606	30	0.8621
	3: Nd-1-1 vs. 7: Pathogen	52.37	44.63 to 60.12	2.101	35.25	30	<0.0001
	3: Nd-1-1 vs. 7: N2-1-1	33.19	25.45 to 40.93	2.101	22.34	30	<0.0001
	3: Nd-1-1 vs. 7: Nd-1-1	28.39	20.64 to 36.13	2.101	19.11	30	<0.0001
	3: Nd-1-1 vs. 7: Pb-2-2	26.05	18.31 to 33.80	2.101	17.54	30	<0.0001
	3: Pb-2-2 vs. 5: Blank	−8.211	−15.95 to −0.4676	2.101	5.526	30	0.0296
	3: Pb-2-2 vs. 5: Pathogen	45.89	38.15 to 53.64	2.101	30.89	30	<0.0001
	3: Pb-2-2 vs. 5: N2-1-1	21.88	14.13 to 29.62	2.101	14.72	30	<0.0001
	3: Pb-2-2 vs. 5: Nd-1-1	20.63	12.89 to 28.38	2.101	13.89	30	<0.0001
	3: Pb-2-2 vs. 5: Pb-2-2	20.53	12.79 to 28.27	2.101	13.82	30	<0.0001
	3: Pb-2-2 vs. 7: Blank	4.344	−3.400 to 12.09	2.101	2.923	30	0.7447
	3: Pb-2-2 vs. 7: Pathogen	52.85	45.10 to 60.59	2.101	35.57	30	<0.0001
	3: Pb-2-2 vs. 7: N2-1-1	33.66	25.92 to 41.40	2.101	22.65	30	<0.0001
	3: Pb-2-2 vs. 7: Nd-1-1	28.86	21.12 to 36.60	2.101	19.42	30	<0.0001
	3: Pb-2-2 vs. 7: Pb-2-2	26.53	18.78 to 34.27	2.101	17.85	30	<0.0001
	5: Blank vs. 5: Pathogen	54.1	46.36 to 61.85	2.101	36.41	30	<0.0001
	5: Blank vs. 5: N2-1-1	30.09	22.34 to 37.83	2.101	20.25	30	<0.0001
	5: Blank vs. 5: Nd-1-1	28.85	21.10 to 36.59	2.101	19.41	30	<0.0001
	5: Blank vs. 5: Pb-2-2	28.74	21.00 to 36.48	2.101	19.34	30	<0.0001
	5: Blank vs. 7: Blank	12.55	4.811 to 20.30	2.101	8.449	30	0.0001
	5: Blank vs. 7: Pathogen	61.06	53.31 to 68.80	2.101	41.09	30	<0.0001
	5: Blank vs. 7: N2-1-1	41.87	34.13 to 49.62	2.101	28.18	30	<0.0001
	5: Blank vs. 7: Nd-1-1	37.07	29.33 to 44.81	2.101	24.95	30	<0.0001
	5: Blank vs. 7: Pb-2-2	34.74	26.99 to 42.48	2.101	23.38	30	<0.0001
	5: Pathogen vs. 5: N2-1-1	−24.02	−31.76 to −16.27	2.101	16.16	30	<0.0001
	5: Pathogen vs. 5: Nd-1-1	−25.26	−33.00 to −17.51	2.101	17	30	<0.0001
	5: Pathogen vs. 5: Pb-2-2	−25.36	−33.11 to −17.62	2.101	17.07	30	<0.0001
	5: Pathogen vs. 7: Blank	−41.55	−49.29 to −33.81	2.101	27.96	30	<0.0001
	5: Pathogen vs. 7: Pathogen	6.953	−0.7909 to 14.70	2.101	4.679	30	0.1145
	5: Pathogen vs. 7: N2-1-1	−12.23	−19.97 to −4.488	2.101	8.232	30	0.0002
	5: Pathogen vs. 7: Nd-1-1	−17.03	−24.78 to −9.289	2.101	11.46	30	<0.0001
	5: Pathogen vs. 7: Pb-2-2	−19.37	−27.11 to −11.62	2.101	13.03	30	<0.0001
	5: N2-1-1 vs. 5: Nd-1-1	−1.242	−8.986 to 6.501	2.101	0.8362	30	>0.9999
	5: N2-1-1 vs. 5: Pb-2-2	−1.348	−9.091 to 6.395	2.101	0.9072	30	>0.9999
	5: N2-1-1 vs. 7: Blank	−17.53	−25.28 to −9.790	2.101	11.8	30	<0.0001
	5: N2-1-1 vs. 7: Pathogen	30.97	23.22 to 38.71	2.101	20.84	30	<0.0001
	5: N2-1-1 vs. 7: N2-1-1	11.78	4.040 to 19.53	2.101	7.931	30	0.0003
	5: N2-1-1 vs. 7: Nd-1-1	6.983	−0.7604 to 14.73	2.101	4.7	30	0.1111
	5: N2-1-1 vs. 7: Pb-2-2	4.649	−3.094 to 12.39	2.101	3.129	30	0.6546
	5: Nd-1-1 vs. 5: Pb-2-2	−0.1055	−7.849 to 7.638	2.101	0.07102	30	>0.9999
	5: Nd-1-1 vs. 7: Blank	−16.29	−24.03 to −8.548	2.101	10.96	30	<0.0001
	5: Nd-1-1 vs. 7: Pathogen	32.21	24.47 to 39.95	2.101	21.68	30	<0.0001
	5: Nd-1-1 vs. 7: N2-1-1	13.03	5.283 to 20.77	2.101	8.767	30	<0.0001
	5: Nd-1-1 vs. 7: Nd-1-1	8.225	0.4820 to 15.97	2.101	5.536	30	0.0291
	5: Nd-1-1 vs. 7: Pb-2-2	5.892	−1.851 to 13.64	2.101	3.965	30	0.2969
	5: Pb-2-2 vs. 7: Blank	−16.19	−23.93 to −8.442	2.101	10.89	30	<0.0001
	5: Pb-2-2 vs. 7: Pathogen	32.32	24.57 to 40.06	2.101	21.75	30	<0.0001
	5: Pb-2-2 vs. 7: N2-1-1	13.13	5.388 to 20.88	2.101	8.838	30	<0.0001
	5: Pb-2-2 vs. 7: Nd-1-1	8.331	0.5876 to 16.07	2.101	5.607	30	0.0258
	5: Pb-2-2 vs. 7: Pb-2-2	5.997	−1.746 to 13.74	2.101	4.036	30	0.2728
	7: Blank vs. 7: Pathogen	48.5	40.76 to 56.24	2.101	32.64	30	<0.0001
	7: Blank vs. 7: N2-1-1	29.32	21.57 to 37.06	2.101	19.73	30	<0.0001
	7: Blank vs. 7: Nd-1-1	24.52	16.77 to 32.26	2.101	16.5	30	<0.0001
	7: Blank vs. 7: Pb-2-2	22.18	14.44 to 29.93	2.101	14.93	30	<0.0001
	7: Pathogen vs. 7: N2-1-1	−19.18	−26.93 to −11.44	2.101	12.91	30	<0.0001
	7: Pathogen vs. 7: Nd-1-1	−23.98	−31.73 to −16.24	2.101	16.14	30	<0.0001
	7: Pathogen vs. 7: Pb-2-2	−26.32	−34.06 to −18.57	2.101	17.71	30	<0.0001
	7: N2-1-1 vs. 7: Nd-1-1	−4.801	−12.54 to 2.943	2.101	3.231	30	0.6079
*Phomopsis*	7: N2-1-1 vs. 7: Pb-2-2	−7.134	−14.88 to 0.6090	2.101	4.802	30	0.0954
	7: Nd-1-1 vs. 7: Pb-2-2	−2.334	−10.08 to 5.410	2.101	1.57	30	0.9978
	3: Blank vs. 3: Pathogen	16.94	9.941 to 23.93	1.899	12.62	30	<0.0001
	3: Blank vs. 3: N2-1-1	−2.454	−9.450 to 4.543	1.899	1.827	30	0.9905
	3: Blank vs. 3: Nd-1-1	−7.658	−14.65 to −0.6611	1.899	5.704	30	0.0218
	3: Blank vs. 3: Pb-2-2	−4.426	−11.42 to 2.571	1.899	3.297	30	0.5776
	3: Blank vs. 5: Blank	−12.44	−19.44 to −5.443	1.899	9.266	30	<0.0001
	3: Blank vs. 5: Pathogen	29.71	22.71 to 36.71	1.899	22.13	30	<0.0001
	3: Blank vs. 5: N2-1-1	6.809	−0.1872 to 13.81	1.899	5.072	30	0.0626
	3: Blank vs. 5: Nd-1-1	9.123	2.127 to 16.12	1.899	6.796	30	0.003
	3: Blank vs. 5: Pb-2-2	13.26	6.259 to 20.25	1.899	9.874	30	<0.0001
	3: Blank vs. 7: Blank	0.115	−6.882 to 7.112	1.899	0.08563	30	>0.9999
	3: Blank vs. 7: Pathogen	46.33	39.33 to 53.32	1.899	34.5	30	<0.0001
	3: Blank vs. 7: N2-1-1	27.64	20.64 to 34.64	1.899	20.59	30	<0.0001
	3: Blank vs. 7: Nd-1-1	24.64	17.65 to 31.64	1.899	18.35	30	<0.0001
	3: Blank vs. 7: Pb-2-2	27.39	20.40 to 34.39	1.899	20.4	30	<0.0001
	3: Pathogen vs. 3: N2-1-1	−19.39	−26.39 to −12.39	1.899	14.44	30	<0.0001
	3: Pathogen vs. 3: Nd-1-1	−24.6	−31.59 to −17.60	1.899	18.32	30	<0.0001
	3: Pathogen vs. 3: Pb-2-2	−21.36	−28.36 to −14.37	1.899	15.91	30	<0.0001
	3: Pathogen vs. 5: Blank	−29.38	−36.37 to −22.38	1.899	21.88	30	<0.0001
	3: Pathogen vs. 5: Pathogen	12.77	5.775 to 19.77	1.899	9.513	30	<0.0001
	3: Pathogen vs. 5: N2-1-1	−10.13	−17.12 to −3.131	1.899	7.544	30	0.0007
	3: Pathogen vs. 5: Nd-1-1	−7.814	−14.81 to −0.8174	1.899	5.82	30	0.0178
	3: Pathogen vs. 5: Pb-2-2	−3.682	−10.68 to 3.315	1.899	2.742	30	0.8158
	3: Pathogen vs. 7: Blank	−16.82	−23.82 to −9.826	1.899	12.53	30	<0.0001
	3: Pathogen vs. 7: Pathogen	29.39	22.39 to 36.38	1.899	21.89	30	<0.0001
	3: Pathogen vs. 7: N2-1-1	10.7	3.705 to 17.70	1.899	7.971	30	0.0003
	3: Pathogen vs. 7: Nd-1-1	7.705	0.7084 to 14.70	1.899	5.739	30	0.0205
	3: Pathogen vs. 7: Pb-2-2	10.46	3.460 to 17.45	1.899	7.789	30	0.0005
	3: N2-1-1 vs. 3: Nd-1-1	−5.204	−12.20 to 1.792	1.899	3.876	30	0.3291
	3: N2-1-1 vs. 3: Pb-2-2	−1.972	−8.969 to 5.024	1.899	1.469	30	0.9989
	3: N2-1-1 vs. 5: Blank	−9.986	−16.98 to −2.990	1.899	7.438	30	0.0009
	3: N2-1-1 vs. 5: Pathogen	32.16	25.17 to 39.16	1.899	23.96	30	<0.0001
	3: N2-1-1 vs. 5: N2-1-1	9.263	2.266 to 16.26	1.899	6.899	30	0.0025
	3: N2-1-1 vs. 5: Nd-1-1	11.58	4.580 to 18.57	1.899	8.623	30	<0.0001
	3: N2-1-1 vs. 5: Pb-2-2	15.71	8.713 to 22.71	1.899	11.7	30	<0.0001
	3: N2-1-1 vs. 7: Blank	2.569	−4.428 to 9.565	1.899	1.913	30	0.9858
	3: N2-1-1 vs. 7: Pathogen	48.78	41.78 to 55.78	1.899	36.33	30	<0.0001
	3: N2-1-1 vs. 7: N2-1-1	30.09	23.10 to 37.09	1.899	22.41	30	<0.0001
	3: N2-1-1 vs. 7: Nd-1-1	27.1	20.10 to 34.09	1.899	20.18	30	<0.0001
	3: N2-1-1 vs. 7: Pb-2-2	29.85	22.85 to 36.84	1.899	22.23	30	<0.0001
	3: Nd-1-1 vs. 3: Pb-2-2	3.232	−3.765 to 10.23	1.899	2.407	30	0.9163
	3: Nd-1-1 vs. 5: Blank	−4.782	−11.78 to 2.215	1.899	3.562	30	0.4574
	3: Nd-1-1 vs. 5: Pathogen	37.37	30.37 to 44.36	1.899	27.83	30	<0.0001
	3: Nd-1-1 vs. 5: N2-1-1	14.47	7.471 to 21.46	1.899	10.78	30	<0.0001
	3: Nd-1-1 vs. 5: Nd-1-1	16.78	9.785 to 23.78	1.899	12.5	30	<0.0001
	3: Nd-1-1 vs. 5: Pb-2-2	20.91	13.92 to 27.91	1.899	15.58	30	<0.0001
	3: Nd-1-1 vs. 7: Blank	7.773	0.7761 to 14.77	1.899	5.789	30	0.0188
	3: Nd-1-1 vs. 7: Pathogen	53.98	46.99 to 60.98	1.899	40.21	30	<0.0001
	3: Nd-1-1 vs. 7: N2-1-1	35.3	28.30 to 42.29	1.899	26.29	30	<0.0001
	3: Nd-1-1 vs. 7: Nd-1-1	32.3	25.30 to 39.30	1.899	24.06	30	<0.0001
	3: Nd-1-1 vs. 7: Pb-2-2	35.05	28.06 to 42.05	1.899	26.11	30	<0.0001
	3: Pb-2-2 vs. 5: Blank	−8.014	−15.01 to −1.017	1.899	5.969	30	0.0137
	3: Pb-2-2 vs. 5: Pathogen	34.14	27.14 to 41.13	1.899	25.43	30	<0.0001
	3: Pb-2-2 vs. 5: N2-1-1	11.24	4.239 to 18.23	1.899	8.369	30	0.0001
	3: Pb-2-2 vs. 5: Nd-1-1	13.55	6.553 to 20.55	1.899	10.09	30	<0.0001
	3: Pb-2-2 vs. 5: Pb-2-2	17.68	10.69 to 24.68	1.899	13.17	30	<0.0001
	3: Pb-2-2 vs. 7: Blank	4.541	−2.456 to 11.54	1.899	3.382	30	0.5382
	3: Pb-2-2 vs. 7: Pathogen	50.75	43.75 to 57.75	1.899	37.8	30	<0.0001
	3: Pb-2-2 vs. 7: N2-1-1	32.06	25.07 to 39.06	1.899	23.88	30	<0.0001
	3: Pb-2-2 vs. 7: Nd-1-1	29.07	22.07 to 36.07	1.899	21.65	30	<0.0001
	3: Pb-2-2 vs. 7: Pb-2-2	31.82	24.82 to 38.82	1.899	23.7	30	<0.0001
	5: Blank vs. 5: Pathogen	42.15	35.15 to 49.15	1.899	31.39	30	<0.0001
	5: Blank vs. 5: N2-1-1	19.25	12.25 to 26.25	1.899	14.34	30	<0.0001
	5: Blank vs. 5: Nd-1-1	21.56	14.57 to 28.56	1.899	16.06	30	<0.0001
	5: Blank vs. 5: Pb-2-2	25.7	18.70 to 32.69	1.899	19.14	30	<0.0001
	5: Blank vs. 7: Blank	12.55	5.558 to 19.55	1.899	9.351	30	<0.0001
	5: Blank vs. 7: Pathogen	58.77	51.77 to 65.76	1.899	43.77	30	<0.0001
	5: Blank vs. 7: N2-1-1	40.08	33.08 to 47.08	1.899	29.85	30	<0.0001
	5: Blank vs. 7: Nd-1-1	37.08	30.09 to 44.08	1.899	27.62	30	<0.0001
	5: Blank vs. 7: Pb-2-2	39.83	32.84 to 46.83	1.899	29.67	30	<0.0001
	5: Pathogen vs. 5: N2-1-1	−22.9	−29.90 to −15.90	1.899	17.06	30	<0.0001
	5: Pathogen vs. 5: Nd-1-1	−20.59	−27.58 to −13.59	1.899	15.33	30	<0.0001
	5: Pathogen vs. 5: Pb-2-2	−16.45	−23.45 to −9.457	1.899	12.26	30	<0.0001
	5: Pathogen vs. 7: Blank	−29.59	−36.59 to −22.60	1.899	22.04	30	<0.0001
	5: Pathogen vs. 7: Pathogen	16.62	9.619 to 23.61	1.899	12.38	30	<0.0001
	5: Pathogen vs. 7: N2-1-1	−2.071	−9.067 to 4.926	1.899	1.542	30	0.9982
	5: Pathogen vs. 7: Nd-1-1	−5.067	−12.06 to 1.930	1.899	3.774	30	0.3685
	5: Pathogen vs. 7: Pb-2-2	−2.315	−9.312 to 4.682	1.899	1.724	30	0.9945
	5: N2-1-1 vs. 5: Nd-1-1	2.314	−4.683 to 9.311	1.899	1.724	30	0.9945
	5: N2-1-1 vs. 5: Pb-2-2	6.447	−0.5501 to 13.44	1.899	4.802	30	0.0954
	5: N2-1-1 vs. 7: Blank	−6.695	−13.69 to 0.3022	1.899	4.986	30	0.0717
	5: N2-1-1 vs. 7: Pathogen	39.52	32.52 to 46.51	1.899	29.43	30	<0.0001
	5: N2-1-1 vs. 7: N2-1-1	20.83	13.83 to 27.83	1.899	15.51	30	<0.0001
	5: N2-1-1 vs. 7: Nd-1-1	17.83	10.84 to 24.83	1.899	13.28	30	<0.0001
	5: N2-1-1 vs. 7: Pb-2-2	20.58	13.59 to 27.58	1.899	15.33	30	<0.0001
	5: Nd-1-1 vs. 5: Pb-2-2	4.133	−2.864 to 11.13	1.899	3.078	30	0.6776
	5: Nd-1-1 vs. 7: Blank	−9.009	−16.01 to −2.012	1.899	6.71	30	0.0035
	5: Nd-1-1 vs. 7: Pathogen	37.2	30.21 to 44.20	1.899	27.71	30	<0.0001
	5: Nd-1-1 vs. 7: N2-1-1	18.52	11.52 to 25.51	1.899	13.79	30	<0.0001
	5: Nd-1-1 vs. 7: Nd-1-1	15.52	8.522 to 22.52	1.899	11.56	30	<0.0001
	5: Nd-1-1 vs. 7: Pb-2-2	18.27	11.27 to 25.27	1.899	13.61	30	<0.0001
	5: Pb-2-2 vs. 7: Blank	−13.14	−20.14 to −6.144	1.899	9.788	30	<0.0001
	5: Pb-2-2 vs. 7: Pathogen	33.07	26.07 to 40.07	1.899	24.63	30	<0.0001
	5: Pb-2-2 vs. 7: N2-1-1	14.38	7.386 to 21.38	1.899	10.71	30	<0.0001
	5: Pb-2-2 vs. 7: Nd-1-1	11.39	4.390 to 18.38	1.899	8.481	30	0.0001
	5: Pb-2-2 vs. 7: Pb-2-2	14.14	7.142 to 21.13	1.899	10.53	30	<0.0001
	7: Blank vs. 7: Pathogen	46.21	39.21 to 53.21	1.899	34.42	30	<0.0001
	7: Blank vs. 7: N2-1-1	27.52	20.53 to 34.52	1.899	20.5	30	<0.0001
	7: Blank vs. 7: Nd-1-1	24.53	17.53 to 31.52	1.899	18.27	30	<0.0001
	7: Blank vs. 7: Pb-2-2	27.28	20.28 to 34.28	1.899	20.32	30	<0.0001
	7: Pathogen vs. 7: N2-1-1	−18.69	−25.68 to −11.69	1.899	13.92	30	<0.0001
	7: Pathogen vs. 7: Nd-1-1	−21.68	−28.68 to −14.69	1.899	16.15	30	<0.0001
	7: Pathogen vs. 7: Pb-2-2	−18.93	−25.93 to −11.93	1.899	14.1	30	<0.0001
	7: N2-1-1 vs. 7: Nd-1-1	−2.996	−9.993 to 4.000	1.899	2.232	30	0.9509
	7: N2-1-1 vs. 7: Pb-2-2	−0.2446	−7.241 to 6.752	1.899	0.1822	30	>0.9999
	7: Nd-1-1 vs. 7: Pb-2-2	2.752	−4.245 to 9.748	1.899	2.05	30	0.9746

Note: SOD, superoxide dismutase. The comparisons are denoted as “*time point*:*treatment*”, where time points “3”, “5”, and “7” indicate days post-inoculation; “Pathogen” refers to the original pathogen isolate; “N2-1-1”, “Nd-1-1”, and “Pb-2-2” are distinct microbial strains/isolates; “Blank” represents the non-inoculated control. For each pairwise comparison, the mean difference, 95% confidence interval (CI) of the difference, standard error of the difference (SE), studentized range statistic (q), degrees of freedom (df), and adjusted *p*-value (Tukey’s method) are presented. Due to a balanced design and homogeneity of variance, the SE of the difference (1.899) and the degrees of freedom (30) are constant across all comparisons. The critical significance level was set at α = 0.05, and adjusted *p*-values were computed to control the family-wise error rate.

**Table A7:** Tukey’s multiple comparisons test for CAT enzyme activity.

Pathogen	Comparison	Mean Difference	95.00% CI	SE of Difference	q	*df*	Adjusted *p*
*Alternaria*	3: Blank vs. 3: Pathogen	−1.634	−5.572 to 2.303	1.068	2.163	30	0.9611
	3: Blank vs. 3: N2-1-1	−7.761	−11.70 to −3.824	1.068	10.27	30	<0.0001
	3: Blank vs. 3: Nd-1-1	−6.704	−10.64 to −2.766	1.068	8.873	30	<0.0001
	3: Blank vs. 3: Pb-2-2	−6.723	−10.66 to −2.786	1.068	8.899	30	<0.0001
	3: Blank vs. 5: Blank	−8.344	−12.28 to −4.407	1.068	11.04	30	<0.0001
	3: Blank vs. 5: Pathogen	−1.475	−5.413 to 2.462	1.068	1.953	30	0.983
	3: Blank vs. 5: N2-1-1	−5.741	−9.678 to −1.803	1.068	7.599	30	0.0007
	3: Blank vs. 5: Nd-1-1	−3.103	−7.041 to 0.8338	1.068	4.108	30	0.2499
	3: Blank vs. 5: Pb-2-2	−5.383	−9.320 to −1.446	1.068	7.125	30	0.0016
	3: Blank vs. 7: Blank	−17.59	−21.52 to −13.65	1.068	23.28	30	<0.0001
	3: Blank vs. 7: Pathogen	1.99	−1.948 to 5.927	1.068	2.634	30	0.8532
	3: Blank vs. 7: N2-1-1	0.1425	−3.795 to 4.080	1.068	0.1886	30	>0.9999
	3: Blank vs. 7: Nd-1-1	0.4951	−3.442 to 4.432	1.068	0.6554	30	>0.9999
	3: Blank vs. 7: Pb-2-2	−0.7974	−4.735 to 3.140	1.068	1.055	30	>0.9999
	3: Pathogen vs. 3: N2-1-1	−6.127	−10.06 to −2.190	1.068	8.109	30	0.0002
	3: Pathogen vs. 3: Nd-1-1	−5.069	−9.006 to −1.132	1.068	6.71	30	0.0035
	3: Pathogen vs. 3: Pb-2-2	−5.089	−9.026 to −1.151	1.068	6.735	30	0.0034
	3: Pathogen vs. 5: Blank	−6.71	−10.65 to −2.773	1.068	8.881	30	<0.0001
	3: Pathogen vs. 5: Pathogen	0.1589	−3.778 to 4.096	1.068	0.2104	30	>0.9999
	3: Pathogen vs. 5: N2-1-1	−4.106	−8.044 to −0.1691	1.068	5.435	30	0.0345
	3: Pathogen vs. 5: Nd-1-1	−1.469	−5.406 to 2.468	1.068	1.944	30	0.9837
	3: Pathogen vs. 5: Pb-2-2	−3.749	−7.686 to 0.1887	1.068	4.962	30	0.0745
	3: Pathogen vs. 7: Blank	−15.95	−19.89 to −12.02	1.068	21.12	30	<0.0001
	3: Pathogen vs. 7: Pathogen	3.624	−0.3132 to 7.561	1.068	4.797	30	0.0961
	3: Pathogen vs. 7: N2-1-1	1.777	−2.160 to 5.714	1.068	2.352	30	0.9285
	3: Pathogen vs. 7: Nd-1-1	2.13	−1.808 to 6.067	1.068	2.819	30	0.787
	3: Pathogen vs. 7: Pb-2-2	0.8369	−3.100 to 4.774	1.068	1.108	30	>0.9999
	3: N2-1-1 vs. 3: Nd-1-1	1.058	−2.880 to 4.995	1.068	1.4	30	0.9993
	3: N2-1-1 vs. 3: Pb-2-2	1.038	−2.899 to 4.975	1.068	1.374	30	0.9995
	3: N2-1-1 vs. 5: Blank	−0.5832	−4.520 to 3.354	1.068	0.772	30	>0.9999
	3: N2-1-1 vs. 5: Pathogen	6.286	2.348 to 10.22	1.068	8.32	30	0.0002
	3: N2-1-1 vs. 5: N2-1-1	2.02	−1.917 to 5.958	1.068	2.674	30	0.8397
	3: N2-1-1 vs. 5: Nd-1-1	4.658	0.7205 to 8.595	1.068	6.165	30	0.0096
	3: N2-1-1 vs. 5: Pb-2-2	2.378	−1.559 to 6.315	1.068	3.148	30	0.6461
	3: N2-1-1 vs. 7: Blank	−9.826	−13.76 to −5.889	1.068	13.01	30	<0.0001
	3: N2-1-1 vs. 7: Pathogen	9.751	5.814 to 13.69	1.068	12.91	30	<0.0001
	3: N2-1-1 vs. 7: N2-1-1	7.904	3.966 to 11.84	1.068	10.46	30	<0.0001
	3: N2-1-1 vs. 7: Nd-1-1	8.256	4.319 to 12.19	1.068	10.93	30	<0.0001
	3: N2-1-1 vs. 7: Pb-2-2	6.964	3.026 to 10.90	1.068	9.217	30	<0.0001
	3: Nd-1-1 vs. 3: Pb-2-2	−0.01957	−3.957 to 3.918	1.068	0.0259	30	>0.9999
	3: Nd-1-1 vs. 5: Blank	−1.641	−5.578 to 2.296	1.068	2.172	30	0.9599
	3: Nd-1-1 vs. 5: Pathogen	5.228	1.291 to 9.165	1.068	6.92	30	0.0024
	3: Nd-1-1 vs. 5: N2-1-1	0.9628	−2.974 to 4.900	1.068	1.274	30	0.9998
	3: Nd-1-1 vs. 5: Nd-1-1	3.6	−0.3371 to 7.537	1.068	4.765	30	0.1008
	3: Nd-1-1 vs. 5: Pb-2-2	1.321	−2.617 to 5.258	1.068	1.748	30	0.9937
	3: Nd-1-1 vs. 7: Blank	−10.88	−14.82 to −6.946	1.068	14.41	30	<0.0001
	3: Nd-1-1 vs. 7: Pathogen	8.693	4.756 to 12.63	1.068	11.51	30	<0.0001
	3: Nd-1-1 vs. 7: N2-1-1	6.846	2.909 to 10.78	1.068	9.061	30	<0.0001
	3: Nd-1-1 vs. 7: Nd-1-1	7.199	3.261 to 11.14	1.068	9.528	30	<0.0001
	3: Nd-1-1 vs. 7: Pb-2-2	5.906	1.969 to 9.843	1.068	7.817	30	0.0004
	3: Pb-2-2 vs. 5: Blank	−1.621	−5.559 to 2.316	1.068	2.146	30	0.9635
	3: Pb-2-2 vs. 5: Pathogen	5.248	1.310 to 9.185	1.068	6.946	30	0.0023
	3: Pb-2-2 vs. 5: N2-1-1	0.9823	−2.955 to 4.920	1.068	1.3	30	0.9997
	3: Pb-2-2 vs. 5: Nd-1-1	3.62	−0.3176 to 7.557	1.068	4.791	30	0.0969
	3: Pb-2-2 vs. 5: Pb-2-2	1.34	−2.597 to 5.277	1.068	1.774	30	0.9928
	3: Pb-2-2 vs. 7: Blank	−10.86	−14.80 to −6.927	1.068	14.38	30	<0.0001
	3: Pb-2-2 vs. 7: Pathogen	8.713	4.775 to 12.65	1.068	11.53	30	<0.0001
	3: Pb-2-2 vs. 7: N2-1-1	6.866	2.928 to 10.80	1.068	9.087	30	<0.0001
	3: Pb-2-2 vs. 7: Nd-1-1	7.218	3.281 to 11.16	1.068	9.554	30	<0.0001
	3: Pb-2-2 vs. 7: Pb-2-2	5.926	1.988 to 9.863	1.068	7.843	30	0.0004
	5: Blank vs. 5: Pathogen	6.869	2.932 to 10.81	1.068	9.092	30	<0.0001
	5: Blank vs. 5: N2-1-1	2.604	−1.334 to 6.541	1.068	3.446	30	0.509
	5: Blank vs. 5: Nd-1-1	5.241	1.304 to 9.178	1.068	6.937	30	0.0023
	5: Blank vs. 5: Pb-2-2	2.961	−0.9758 to 6.899	1.068	3.92	30	0.3131
	5: Blank vs. 7: Blank	−9.243	−13.18 to −5.306	1.068	12.23	30	<0.0001
	5: Blank vs. 7: Pathogen	10.33	6.397 to 14.27	1.068	13.68	30	<0.0001
	5: Blank vs. 7: N2-1-1	8.487	4.550 to 12.42	1.068	11.23	30	<0.0001
	5: Blank vs. 7: Nd-1-1	8.84	4.902 to 12.78	1.068	11.7	30	<0.0001
	5: Blank vs. 7: Pb-2-2	7.547	3.610 to 11.48	1.068	9.989	30	<0.0001
	5: Pathogen vs. 5: N2-1-1	−4.265	−8.203 to −0.3281	1.068	5.646	30	0.0241
	5: Pathogen vs. 5: Nd-1-1	−1.628	−5.565 to 2.309	1.068	2.155	30	0.9623
	5: Pathogen vs. 5: Pb-2-2	−3.908	−7.845 to 0.02974	1.068	5.172	30	0.0533
	5: Pathogen vs. 7: Blank	−16.11	−20.05 to −12.17	1.068	21.33	30	<0.0001
	5: Pathogen vs. 7: Pathogen	3.465	−0.4722 to 7.402	1.068	4.586	30	0.1311
	5: Pathogen vs. 7: N2-1-1	1.618	−2.319 to 5.555	1.068	2.142	30	0.964
	5: Pathogen vs. 7: Nd-1-1	1.971	−1.967 to 5.908	1.068	2.608	30	0.8613
	5: Pathogen vs. 7: Pb-2-2	0.678	−3.259 to 4.615	1.068	0.8974	30	>0.9999
	5: N2-1-1 vs. 5: Nd-1-1	2.637	−1.300 to 6.575	1.068	3.491	30	0.4889
	5: N2-1-1 vs. 5: Pb-2-2	0.3578	−3.579 to 4.295	1.068	0.4736	30	>0.9999
	5: N2-1-1 vs. 7: Blank	−11.85	−15.78 to −7.909	1.068	15.68	30	<0.0001
	5: N2-1-1 vs. 7: Pathogen	7.73	3.793 to 11.67	1.068	10.23	30	<0.0001
	5: N2-1-1 vs. 7: N2-1-1	5.883	1.946 to 9.820	1.068	7.787	30	0.0005
	5: N2-1-1 vs. 7: Nd-1-1	6.236	2.299 to 10.17	1.068	8.254	30	0.0002
	5: N2-1-1 vs. 7: Pb-2-2	4.943	1.006 to 8.881	1.068	6.543	30	0.0048
	5: Nd-1-1 vs. 5: Pb-2-2	−2.28	−6.217 to 1.658	1.068	3.017	30	0.7045
	5: Nd-1-1 vs. 7: Blank	−14.48	−18.42 to −10.55	1.068	19.17	30	<0.0001
	5: Nd-1-1 vs. 7: Pathogen	5.093	1.156 to 9.030	1.068	6.741	30	0.0033
	5: Nd-1-1 vs. 7: N2-1-1	3.246	−0.6913 to 7.183	1.068	4.296	30	0.196
	5: Nd-1-1 vs. 7: Nd-1-1	3.599	−0.3387 to 7.536	1.068	4.763	30	0.1011
	5: Nd-1-1 vs. 7: Pb-2-2	2.306	−1.631 to 6.243	1.068	3.052	30	0.6891
	5: Pb-2-2 vs. 7: Blank	−12.2	−16.14 to −8.267	1.068	16.15	30	<0.0001
	5: Pb-2-2 vs. 7: Pathogen	7.373	3.435 to 11.31	1.068	9.758	30	<0.0001
	5: Pb-2-2 vs. 7: N2-1-1	5.525	1.588 to 9.463	1.068	7.314	30	0.0011
	5: Pb-2-2 vs. 7: Nd-1-1	5.878	1.941 to 9.815	1.068	7.78	30	0.0005
	5: Pb-2-2 vs. 7: Pb-2-2	4.586	0.6483 to 8.523	1.068	6.069	30	0.0114
	7: Blank vs. 7: Pathogen	19.58	15.64 to 23.51	1.068	25.91	30	<0.0001
	7: Blank vs. 7: N2-1-1	17.73	13.79 to 21.67	1.068	23.47	30	<0.0001
	7: Blank vs. 7: Nd-1-1	18.08	14.15 to 22.02	1.068	23.93	30	<0.0001
	7: Blank vs. 7: Pb-2-2	16.79	12.85 to 20.73	1.068	22.22	30	<0.0001
	7: Pathogen vs. 7: N2-1-1	−1.847	−5.784 to 2.090	1.068	2.445	30	0.9073
	7: Pathogen vs. 7: Nd-1-1	−1.495	−5.432 to 2.443	1.068	1.978	30	0.9811
	7: Pathogen vs. 7: Pb-2-2	−2.787	−6.724 to 1.150	1.068	3.689	30	0.403
	7: N2-1-1 vs. 7: Nd-1-1	0.3526	−3.585 to 4.290	1.068	0.4668	30	>0.9999
*Fusarium*	7: N2-1-1 vs. 7: Pb-2-2	−0.9399	−4.877 to 2.997	1.068	1.244	30	0.9998
	7: Nd-1-1 vs. 7: Pb-2-2	−1.293	−5.230 to 2.645	1.068	1.711	30	0.9949
	3: Blank vs. 3: Pathogen	−6.711	−9.914 to −3.508	0.8692	10.92	30	<0.0001
	3: Blank vs. 3: N2-1-1	−11.7	−14.91 to −8.500	0.8692	19.04	30	<0.0001
	3: Blank vs. 3: Nd-1-1	−9.181	−12.38 to −5.978	0.8692	14.94	30	<0.0001
	3: Blank vs. 3: Pb-2-2	−11.35	−14.55 to −8.145	0.8692	18.46	30	<0.0001
	3: Blank vs. 5: Blank	−8.344	−11.55 to −5.141	0.8692	13.58	30	<0.0001
	3: Blank vs. 5: Pathogen	−3.561	−6.764 to −0.3582	0.8692	5.794	30	0.0186
	3: Blank vs. 5: N2-1-1	−8.506	−11.71 to −5.303	0.8692	13.84	30	<0.0001
	3: Blank vs. 5: Nd-1-1	−7.799	−11.00 to −4.596	0.8692	12.69	30	<0.0001
	3: Blank vs. 5: Pb-2-2	−7.782	−10.98 to −4.578	0.8692	12.66	30	<0.0001
	3: Blank vs. 7: Blank	−17.59	−20.79 to −14.38	0.8692	28.61	30	<0.0001
	3: Blank vs. 7: Pathogen	1.077	−2.126 to 4.280	0.8692	1.753	30	0.9936
	3: Blank vs. 7: N2-1-1	−5.091	−8.294 to −1.888	0.8692	8.283	30	0.0002
	3: Blank vs. 7: Nd-1-1	−6.256	−9.459 to −3.053	0.8692	10.18	30	<0.0001
	3: Blank vs. 7: Pb-2-2	−3.663	−6.866 to −0.4596	0.8692	5.959	30	0.0139
	3: Pathogen vs. 3: N2-1-1	−4.991	−8.195 to −1.788	0.8692	8.121	30	0.0002
	3: Pathogen vs. 3: Nd-1-1	−2.47	−5.673 to 0.7330	0.8692	4.019	30	0.2786
	3: Pathogen vs. 3: Pb-2-2	−4.637	−7.840 to −1.434	0.8692	7.544	30	0.0007
	3: Pathogen vs. 5: Blank	−1.633	−4.836 to 1.570	0.8692	2.657	30	0.8455
	3: Pathogen vs. 5: Pathogen	3.15	−0.05317 to 6.353	0.8692	5.125	30	0.0575
	3: Pathogen vs. 5: N2-1-1	−1.795	−4.998 to 1.408	0.8692	2.92	30	0.7462
	3: Pathogen vs. 5: Nd-1-1	−1.088	−4.291 to 2.115	0.8692	1.77	30	0.9929
	3: Pathogen vs. 5: Pb-2-2	−1.07	−4.274 to 2.133	0.8692	1.742	30	0.9939
	3: Pathogen vs. 7: Blank	−10.88	−14.08 to −7.673	0.8692	17.7	30	<0.0001
	3: Pathogen vs. 7: Pathogen	7.789	4.585 to 10.99	0.8692	12.67	30	<0.0001
	3: Pathogen vs. 7: N2-1-1	1.62	−1.583 to 4.823	0.8692	2.636	30	0.8523
	3: Pathogen vs. 7: Nd-1-1	0.4554	−2.748 to 3.658	0.8692	0.7409	30	>0.9999
	3: Pathogen vs. 7: Pb-2-2	3.048	−0.1546 to 6.252	0.8692	4.96	30	0.0748
	3: N2-1-1 vs. 3: Nd-1-1	2.521	−0.6818 to 5.724	0.8692	4.102	30	0.2516
	3: N2-1-1 vs. 3: Pb-2-2	0.3544	−2.849 to 3.558	0.8692	0.5766	30	>0.9999
	3: N2-1-1 vs. 5: Blank	3.358	0.1552 to 6.561	0.8692	5.464	30	0.0329
	3: N2-1-1 vs. 5: Pathogen	8.141	4.938 to 11.34	0.8692	13.25	30	<0.0001
	3: N2-1-1 vs. 5: N2-1-1	3.197	−0.006269 to 6.400	0.8692	5.201	30	0.0508
	3: N2-1-1 vs. 5: Nd-1-1	3.903	0.7002 to 7.106	0.8692	6.351	30	0.0068
	3: N2-1-1 vs. 5: Pb-2-2	3.921	0.7180 to 7.124	0.8692	6.38	30	0.0065
	3: N2-1-1 vs. 7: Blank	−5.885	−9.088 to −2.681	0.8692	9.574	30	<0.0001
	3: N2-1-1 vs. 7: Pathogen	12.78	9.577 to 15.98	0.8692	20.79	30	<0.0001
	3: N2-1-1 vs. 7: N2-1-1	6.612	3.409 to 9.815	0.8692	10.76	30	<0.0001
	3: N2-1-1 vs. 7: Nd-1-1	5.447	2.244 to 8.650	0.8692	8.862	30	<0.0001
	3: N2-1-1 vs. 7: Pb-2-2	8.04	4.837 to 11.24	0.8692	13.08	30	<0.0001
	3: Nd-1-1 vs. 3: Pb-2-2	−2.167	−5.370 to 1.036	0.8692	3.526	30	0.4734
	3: Nd-1-1 vs. 5: Blank	0.837	−2.366 to 4.040	0.8692	1.362	30	0.9995
	3: Nd-1-1 vs. 5: Pathogen	5.62	2.417 to 8.823	0.8692	9.144	30	<0.0001
	3: Nd-1-1 vs. 5: N2-1-1	0.6755	−2.528 to 3.879	0.8692	1.099	30	>0.9999
	3: Nd-1-1 vs. 5: Nd-1-1	1.382	−1.821 to 4.585	0.8692	2.248	30	0.9481
	3: Nd-1-1 vs. 5: Pb-2-2	1.4	−1.803 to 4.603	0.8692	2.277	30	0.9431
	3: Nd-1-1 vs. 7: Blank	−8.406	−11.61 to −5.203	0.8692	13.68	30	<0.0001
	3: Nd-1-1 vs. 7: Pathogen	10.26	7.056 to 13.46	0.8692	16.69	30	<0.0001
	3: Nd-1-1 vs. 7: N2-1-1	4.09	0.8874 to 7.294	0.8692	6.655	30	0.0039
	3: Nd-1-1 vs. 7: Nd-1-1	2.926	−0.2776 to 6.129	0.8692	4.76	30	0.1016
	3: Nd-1-1 vs. 7: Pb-2-2	5.519	2.315 to 8.722	0.8692	8.979	30	<0.0001
	3: Pb-2-2 vs. 5: Blank	3.004	−0.1992 to 6.207	0.8692	4.887	30	0.0837
	3: Pb-2-2 vs. 5: Pathogen	7.787	4.584 to 10.99	0.8692	12.67	30	<0.0001
	3: Pb-2-2 vs. 5: N2-1-1	2.842	−0.3607 to 6.046	0.8692	4.625	30	0.1241
	3: Pb-2-2 vs. 5: Nd-1-1	3.549	0.3458 to 6.752	0.8692	5.774	30	0.0193
	3: Pb-2-2 vs. 5: Pb-2-2	3.567	0.3636 to 6.770	0.8692	5.803	30	0.0183
	3: Pb-2-2 vs. 7: Blank	−6.239	−9.442 to −3.036	0.8692	10.15	30	<0.0001
	3: Pb-2-2 vs. 7: Pathogen	12.43	9.222 to 15.63	0.8692	20.22	30	<0.0001
	3: Pb-2-2 vs. 7: N2-1-1	6.257	3.054 to 9.461	0.8692	10.18	30	<0.0001
	3: Pb-2-2 vs. 7: Nd-1-1	5.092	1.889 to 8.296	0.8692	8.285	30	0.0002
	3: Pb-2-2 vs. 7: Pb-2-2	7.686	4.482 to 10.89	0.8692	12.5	30	<0.0001
	5: Blank vs. 5: Pathogen	4.783	1.580 to 7.986	0.8692	7.782	30	0.0005
	5: Blank vs. 5: N2-1-1	−0.1615	−3.365 to 3.042	0.8692	0.2627	30	>0.9999
	5: Blank vs. 5: Nd-1-1	0.545	−2.658 to 3.748	0.8692	0.8867	30	>0.9999
	5: Blank vs. 5: Pb-2-2	0.5628	−2.640 to 3.766	0.8692	0.9156	30	>0.9999
	5: Blank vs. 7: Blank	−9.243	−12.45 to −6.040	0.8692	15.04	30	<0.0001
	5: Blank vs. 7: Pathogen	9.422	6.219 to 12.62	0.8692	15.33	30	<0.0001
	5: Blank vs. 7: N2-1-1	3.254	0.05040 to 6.457	0.8692	5.293	30	0.0437
	5: Blank vs. 7: Nd-1-1	2.089	−1.115 to 5.292	0.8692	3.398	30	0.531
	5: Blank vs. 7: Pb-2-2	4.682	1.479 to 7.885	0.8692	7.617	30	0.0006
	5: Pathogen vs. 5: N2-1-1	−4.945	−8.148 to −1.741	0.8692	8.045	30	0.0003
	5: Pathogen vs. 5: Nd-1-1	−4.238	−7.441 to −1.035	0.8692	6.895	30	0.0025
	5: Pathogen vs. 5: Pb-2-2	−4.22	−7.423 to −1.017	0.8692	6.866	30	0.0026
	5: Pathogen vs. 7: Blank	−14.03	−17.23 to −10.82	0.8692	22.82	30	<0.0001
	5: Pathogen vs. 7: Pathogen	4.639	1.435 to 7.842	0.8692	7.547	30	0.0007
	5: Pathogen vs. 7: N2-1-1	−1.53	−4.733 to 1.674	0.8692	2.489	30	0.896
	5: Pathogen vs. 7: Nd-1-1	−2.695	−5.898 to 0.5085	0.8692	4.384	30	0.1742
	5: Pathogen vs. 7: Pb-2-2	−0.1015	−3.305 to 3.102	0.8692	0.1651	30	>0.9999
	5: N2-1-1 vs. 5: Nd-1-1	0.7064	−2.497 to 3.910	0.8692	1.149	30	>0.9999
	5: N2-1-1 vs. 5: Pb-2-2	0.7242	−2.479 to 3.927	0.8692	1.178	30	>0.9999
	5: N2-1-1 vs. 7: Blank	−9.081	−12.28 to −5.878	0.8692	14.78	30	<0.0001
	5: N2-1-1 vs. 7: Pathogen	9.583	6.380 to 12.79	0.8692	15.59	30	<0.0001
	5: N2-1-1 vs. 7: N2-1-1	3.415	0.2119 to 6.618	0.8692	5.556	30	0.0281
	5: N2-1-1 vs. 7: Nd-1-1	2.25	−0.9531 to 5.453	0.8692	3.661	30	0.4148
	5: N2-1-1 vs. 7: Pb-2-2	4.843	1.640 to 8.046	0.8692	7.88	30	0.0004
	5: Nd-1-1 vs. 5: Pb-2-2	0.0178	−3.185 to 3.221	0.8692	0.02896	30	>0.9999
	5: Nd-1-1 vs. 7: Blank	−9.788	−12.99 to −6.585	0.8692	15.92	30	<0.0001
	5: Nd-1-1 vs. 7: Pathogen	8.877	5.674 to 12.08	0.8692	14.44	30	<0.0001
	5: Nd-1-1 vs. 7: N2-1-1	2.709	−0.4946 to 5.912	0.8692	4.407	30	0.1689
	5: Nd-1-1 vs. 7: Nd-1-1	1.544	−1.660 to 4.747	0.8692	2.511	30	0.8899
	5: Nd-1-1 vs. 7: Pb-2-2	4.137	0.9336 to 7.340	0.8692	6.73	30	0.0034
	5: Pb-2-2 vs. 7: Blank	−9.806	−13.01 to −6.602	0.8692	15.95	30	<0.0001
	5: Pb-2-2 vs. 7: Pathogen	8.859	5.656 to 12.06	0.8692	14.41	30	<0.0001
	5: Pb-2-2 vs. 7: N2-1-1	2.691	−0.5124 to 5.894	0.8692	4.378	30	0.1757
	5: Pb-2-2 vs. 7: Nd-1-1	1.526	−1.677 to 4.729	0.8692	2.482	30	0.8977
	5: Pb-2-2 vs. 7: Pb-2-2	4.119	0.9158 to 7.322	0.8692	6.701	30	0.0036
	7: Blank vs. 7: Pathogen	18.66	15.46 to 21.87	0.8692	30.37	30	<0.0001
	7: Blank vs. 7: N2-1-1	12.5	9.293 to 15.70	0.8692	20.33	30	<0.0001
	7: Blank vs. 7: Nd-1-1	11.33	8.128 to 14.53	0.8692	18.44	30	<0.0001
	7: Blank vs. 7: Pb-2-2	13.92	10.72 to 17.13	0.8692	22.65	30	<0.0001
	7: Pathogen vs. 7: N2-1-1	−6.168	−9.371 to −2.965	0.8692	10.04	30	<0.0001
	7: Pathogen vs. 7: Nd-1-1	−7.333	−10.54 to −4.130	0.8692	11.93	30	<0.0001
	7: Pathogen vs. 7: Pb-2-2	−4.74	−7.943 to −1.537	0.8692	7.712	30	0.0005
*Botryosphaeriaceae*	7: N2-1-1 vs. 7: Nd-1-1	−1.165	−4.368 to 2.038	0.8692	1.895	30	0.9869
	7: N2-1-1 vs. 7: Pb-2-2	1.428	−1.775 to 4.631	0.8692	2.324	30	0.9343
	7: Nd-1-1 vs. 7: Pb-2-2	2.593	−0.6100 to 5.796	0.8692	4.219	30	0.217
	3: Blank vs. 3: Pathogen	−4.946	−8.756 to −1.137	1.034	6.766	30	0.0032
	3: Blank vs. 3: N2-1-1	−9.526	−13.34 to −5.716	1.034	13.03	30	<0.0001
	3: Blank vs. 3: Nd-1-1	−9.874	−13.68 to −6.065	1.034	13.51	30	<0.0001
	3: Blank vs. 3: Pb-2-2	−7.153	−10.96 to −3.343	1.034	9.785	30	<0.0001
	3: Blank vs. 5: Blank	−7.344	−11.15 to −3.534	1.034	10.05	30	<0.0001
	3: Blank vs. 5: Pathogen	−1.666	−5.476 to 2.144	1.034	2.279	30	0.9428
	3: Blank vs. 5: N2-1-1	−4.566	−8.376 to −0.7564	1.034	6.246	30	0.0083
	3: Blank vs. 5: Nd-1-1	−5.047	−8.857 to −1.237	1.034	6.904	30	0.0024
	3: Blank vs. 5: Pb-2-2	−2.768	−6.578 to 1.042	1.034	3.787	30	0.3634
	3: Blank vs. 7: Blank	−16.25	−20.06 to −12.44	1.034	22.23	30	<0.0001
	3: Blank vs. 7: Pathogen	5.295	1.485 to 9.105	1.034	7.242	30	0.0013
	3: Blank vs. 7: N2-1-1	−0.9145	−4.724 to 2.895	1.034	1.251	30	0.9998
	3: Blank vs. 7: Nd-1-1	0.9235	−2.886 to 4.733	1.034	1.263	30	0.9998
	3: Blank vs. 7: Pb-2-2	1.25	−2.560 to 5.060	1.034	1.71	30	0.9949
	3: Pathogen vs. 3: N2-1-1	−4.58	−8.390 to −0.7700	1.034	6.265	30	0.008
	3: Pathogen vs. 3: Nd-1-1	−4.928	−8.738 to −1.118	1.034	6.741	30	0.0033
	3: Pathogen vs. 3: Pb-2-2	−2.207	−6.017 to 1.603	1.034	3.019	30	0.7039
	3: Pathogen vs. 5: Blank	−2.398	−6.208 to 1.412	1.034	3.28	30	0.5853
	3: Pathogen vs. 5: Pathogen	3.281	−0.5294 to 7.090	1.034	4.487	30	0.151
	3: Pathogen vs. 5: N2-1-1	0.3802	−3.430 to 4.190	1.034	0.52	30	>0.9999
	3: Pathogen vs. 5: Nd-1-1	−0.1008	−3.911 to 3.709	1.034	0.1379	30	>0.9999
	3: Pathogen vs. 5: Pb-2-2	2.178	−1.632 to 5.988	1.034	2.979	30	0.721
	3: Pathogen vs. 7: Blank	−11.31	−15.12 to −7.498	1.034	15.47	30	<0.0001
	3: Pathogen vs. 7: Pathogen	10.24	6.431 to 14.05	1.034	14.01	30	<0.0001
	3: Pathogen vs. 7: N2-1-1	4.032	0.2220 to 7.842	1.034	5.515	30	0.0301
	3: Pathogen vs. 7: Nd-1-1	5.87	2.060 to 9.680	1.034	8.029	30	0.0003
	3: Pathogen vs. 7: Pb-2-2	6.196	2.387 to 10.01	1.034	8.476	30	0.0001
	3: N2-1-1 vs. 3: Nd-1-1	−0.3481	−4.158 to 3.462	1.034	0.4762	30	>0.9999
	3: N2-1-1 vs. 3: Pb-2-2	2.373	−1.437 to 6.183	1.034	3.246	30	0.601
	3: N2-1-1 vs. 5: Blank	2.182	−1.628 to 5.992	1.034	2.985	30	0.7187
	3: N2-1-1 vs. 5: Pathogen	7.86	4.050 to 11.67	1.034	10.75	30	<0.0001
	3: N2-1-1 vs. 5: N2-1-1	4.96	1.150 to 8.770	1.034	6.785	30	0.0031
	3: N2-1-1 vs. 5: Nd-1-1	4.479	0.6691 to 8.289	1.034	6.127	30	0.0103
	3: N2-1-1 vs. 5: Pb-2-2	6.758	2.948 to 10.57	1.034	9.244	30	<0.0001
	3: N2-1-1 vs. 7: Blank	−6.728	−10.54 to −2.918	1.034	9.202	30	<0.0001
	3: N2-1-1 vs. 7: Pathogen	14.82	11.01 to 18.63	1.034	20.27	30	<0.0001
	3: N2-1-1 vs. 7: N2-1-1	8.612	4.802 to 12.42	1.034	11.78	30	<0.0001
	3: N2-1-1 vs. 7: Nd-1-1	10.45	6.640 to 14.26	1.034	14.29	30	<0.0001
	3: N2-1-1 vs. 7: Pb-2-2	10.78	6.966 to 14.59	1.034	14.74	30	<0.0001
	3: Nd-1-1 vs. 3: Pb-2-2	2.721	−1.089 to 6.531	1.034	3.722	30	0.3893
	3: Nd-1-1 vs. 5: Blank	2.53	−1.280 to 6.340	1.034	3.461	30	0.5024
	3: Nd-1-1 vs. 5: Pathogen	8.209	4.399 to 12.02	1.034	11.23	30	<0.0001
	3: Nd-1-1 vs. 5: N2-1-1	5.308	1.498 to 9.118	1.034	7.261	30	0.0012
	3: Nd-1-1 vs. 5: Nd-1-1	4.827	1.017 to 8.637	1.034	6.603	30	0.0043
	3: Nd-1-1 vs. 5: Pb-2-2	7.106	3.296 to 10.92	1.034	9.72	30	<0.0001
	3: Nd-1-1 vs. 7: Blank	−6.379	−10.19 to −2.570	1.034	8.726	30	<0.0001
	3: Nd-1-1 vs. 7: Pathogen	15.17	11.36 to 18.98	1.034	20.75	30	<0.0001
	3: Nd-1-1 vs. 7: N2-1-1	8.96	5.150 to 12.77	1.034	12.26	30	<0.0001
	3: Nd-1-1 vs. 7: Nd-1-1	10.8	6.988 to 14.61	1.034	14.77	30	<0.0001
	3: Nd-1-1 vs. 7: Pb-2-2	11.12	7.315 to 14.93	1.034	15.22	30	<0.0001
	3: Pb-2-2 vs. 5: Blank	−0.1911	−4.001 to 3.619	1.034	0.2614	30	>0.9999
	3: Pb-2-2 vs. 5: Pathogen	5.487	1.677 to 9.297	1.034	7.506	30	0.0008
	3: Pb-2-2 vs. 5: N2-1-1	2.587	−1.223 to 6.397	1.034	3.539	30	0.4676
	3: Pb-2-2 vs. 5: Nd-1-1	2.106	−1.704 to 5.916	1.034	2.881	30	0.7623
	3: Pb-2-2 vs. 5: Pb-2-2	4.385	0.5750 to 8.195	1.034	5.998	30	0.013
	3: Pb-2-2 vs. 7: Blank	−9.101	−12.91 to −5.291	1.034	12.45	30	<0.0001
	3: Pb-2-2 vs. 7: Pathogen	12.45	8.638 to 16.26	1.034	17.03	30	<0.0001
	3: Pb-2-2 vs. 7: N2-1-1	6.239	2.429 to 10.05	1.034	8.534	30	0.0001
	3: Pb-2-2 vs. 7: Nd-1-1	8.077	4.267 to 11.89	1.034	11.05	30	<0.0001
	3: Pb-2-2 vs. 7: Pb-2-2	8.403	4.593 to 12.21	1.034	11.49	30	<0.0001
	5: Blank vs. 5: Pathogen	5.678	1.869 to 9.488	1.034	7.767	30	0.0005
	5: Blank vs. 5: N2-1-1	2.778	−1.032 to 6.588	1.034	3.8	30	0.3582
	5: Blank vs. 5: Nd-1-1	2.297	−1.513 to 6.107	1.034	3.142	30	0.6487
	5: Blank vs. 5: Pb-2-2	4.576	0.7661 to 8.386	1.034	6.259	30	0.0081
	5: Blank vs. 7: Blank	−8.91	−12.72 to −5.100	1.034	12.19	30	<0.0001
	5: Blank vs. 7: Pathogen	12.64	8.829 to 16.45	1.034	17.29	30	<0.0001
	5: Blank vs. 7: N2-1-1	6.43	2.620 to 10.24	1.034	8.795	30	<0.0001
	5: Blank vs. 7: Nd-1-1	8.268	4.458 to 12.08	1.034	11.31	30	<0.0001
	5: Blank vs. 7: Pb-2-2	8.594	4.784 to 12.40	1.034	11.76	30	<0.0001
	5: Pathogen vs. 5: N2-1-1	−2.9	−6.710 to 0.9095	1.034	3.967	30	0.2963
	5: Pathogen vs. 5: Nd-1-1	−3.381	−7.191 to 0.4285	1.034	4.625	30	0.124
	5: Pathogen vs. 5: Pb-2-2	−1.102	−4.912 to 2.707	1.034	1.508	30	0.9985
	5: Pathogen vs. 7: Blank	−14.59	−18.40 to −10.78	1.034	19.95	30	<0.0001
	5: Pathogen vs. 7: Pathogen	6.961	3.151 to 10.77	1.034	9.521	30	<0.0001
	5: Pathogen vs. 7: N2-1-1	0.7514	−3.059 to 4.561	1.034	1.028	30	>0.9999
	5: Pathogen vs. 7: Nd-1-1	2.589	−1.221 to 6.399	1.034	3.542	30	0.4662
	5: Pathogen vs. 7: Pb-2-2	2.916	−0.8940 to 6.726	1.034	3.989	30	0.2889
	5: N2-1-1 vs. 5: Nd-1-1	−0.481	−4.291 to 3.329	1.034	0.6579	30	>0.9999
	5: N2-1-1 vs. 5: Pb-2-2	1.798	−2.012 to 5.608	1.034	2.459	30	0.9037
	5: N2-1-1 vs. 7: Blank	−11.69	−15.50 to −7.878	1.034	15.99	30	<0.0001
	5: N2-1-1 vs. 7: Pathogen	9.861	6.051 to 13.67	1.034	13.49	30	<0.0001
	5: N2-1-1 vs. 7: N2-1-1	3.652	−0.1582 to 7.462	1.034	4.995	30	0.0707
	5: N2-1-1 vs. 7: Nd-1-1	5.49	1.680 to 9.300	1.034	7.509	30	0.0008
	5: N2-1-1 vs. 7: Pb-2-2	5.816	2.006 to 9.626	1.034	7.956	30	0.0003
	5: Nd-1-1 vs. 5: Pb-2-2	2.279	−1.531 to 6.089	1.034	3.117	30	0.66
	5: Nd-1-1 vs. 7: Blank	−11.21	−15.02 to −7.397	1.034	15.33	30	<0.0001
	5: Nd-1-1 vs. 7: Pathogen	10.34	6.532 to 14.15	1.034	14.15	30	<0.0001
	5: Nd-1-1 vs. 7: N2-1-1	4.133	0.3228 to 7.943	1.034	5.653	30	0.0238
	5: Nd-1-1 vs. 7: Nd-1-1	5.971	2.161 to 9.781	1.034	8.167	30	0.0002
	5: Nd-1-1 vs. 7: Pb-2-2	6.297	2.487 to 10.11	1.034	8.614	30	<0.0001
	5: Pb-2-2 vs. 7: Blank	−13.49	−17.30 to −9.676	1.034	18.45	30	<0.0001
	5: Pb-2-2 vs. 7: Pathogen	8.063	4.253 to 11.87	1.034	11.03	30	<0.0001
	5: Pb-2-2 vs. 7: N2-1-1	1.854	−1.956 to 5.664	1.034	2.536	30	0.883
	5: Pb-2-2 vs. 7: Nd-1-1	3.692	−0.1180 to 7.502	1.034	5.05	30	0.0649
	5: Pb-2-2 vs. 7: Pb-2-2	4.018	0.2085 to 7.828	1.034	5.497	30	0.0311
	7: Blank vs. 7: Pathogen	21.55	17.74 to 25.36	1.034	29.48	30	<0.0001
	7: Blank vs. 7: N2-1-1	15.34	11.53 to 19.15	1.034	20.98	30	<0.0001
	7: Blank vs. 7: Nd-1-1	17.18	13.37 to 20.99	1.034	23.5	30	<0.0001
	7: Blank vs. 7: Pb-2-2	17.5	13.69 to 21.31	1.034	23.94	30	<0.0001
	7: Pathogen vs. 7: N2-1-1	−6.209	−10.02 to −2.399	1.034	8.493	30	0.0001
*Phomopsis*	7: Pathogen vs. 7: Nd-1-1	−4.371	−8.181 to −0.5613	1.034	5.979	30	0.0134
	7: Pathogen vs. 7: Pb-2-2	−4.045	−7.855 to −0.2347	1.034	5.532	30	0.0292
	7: N2-1-1 vs. 7: Nd-1-1	1.838	−1.972 to 5.648	1.034	2.514	30	0.8891
	7: N2-1-1 vs. 7: Pb-2-2	2.165	−1.645 to 5.974	1.034	2.961	30	0.7289
	7: Nd-1-1 vs. 7: Pb-2-2	0.3265	−3.483 to 4.136	1.034	0.4466	30	>0.9999
	3: Blank vs. 3: Pathogen	−3.583	−9.119 to 1.953	1.502	3.373	30	0.5423
	3: Blank vs. 3: N2-1-1	−8.407	−13.94 to −2.871	1.502	7.914	30	0.0004
	3: Blank vs. 3: Nd-1-1	−9.762	−15.30 to −4.226	1.502	9.19	30	<0.0001
	3: Blank vs. 3: Pb-2-2	−5.846	−11.38 to −0.3102	1.502	5.503	30	0.0307
	3: Blank vs. 5: Blank	−9.678	−15.21 to −4.142	1.502	9.11	30	<0.0001
	3: Blank vs. 5: Pathogen	−1.858	−7.394 to 3.678	1.502	1.749	30	0.9937
	3: Blank vs. 5: N2-1-1	−6.673	−12.21 to −1.137	1.502	6.282	30	0.0078
	3: Blank vs. 5: Nd-1-1	−8.829	−14.37 to −3.293	1.502	8.312	30	0.0002
	3: Blank vs. 5: Pb-2-2	−3.118	−8.654 to 2.418	1.502	2.935	30	0.7397
	3: Blank vs. 7: Blank	−17.59	−23.12 to −12.05	1.502	16.56	30	<0.0001
	3: Blank vs. 7: Pathogen	0.9765	−4.559 to 6.513	1.502	0.9193	30	>0.9999
	3: Blank vs. 7: N2-1-1	−2.723	−8.259 to 2.813	1.502	2.563	30	0.875
	3: Blank vs. 7: Nd-1-1	−3.646	−9.182 to 1.890	1.502	3.433	30	0.5152
	3: Blank vs. 7: Pb-2-2	−1.365	−6.901 to 4.171	1.502	1.285	30	0.9997
	3: Pathogen vs. 3: N2-1-1	−4.824	−10.36 to 0.7125	1.502	4.541	30	0.14
	3: Pathogen vs. 3: Nd-1-1	−6.179	−11.72 to −0.6431	1.502	5.817	30	0.0179
	3: Pathogen vs. 3: Pb-2-2	−2.263	−7.799 to 3.273	1.502	2.13	30	0.9655
	3: Pathogen vs. 5: Blank	−6.094	−11.63 to −0.5583	1.502	5.737	30	0.0206
	3: Pathogen vs. 5: Pathogen	1.725	−3.811 to 7.261	1.502	1.624	30	0.9969
	3: Pathogen vs. 5: N2-1-1	−3.09	−8.626 to 2.446	1.502	2.909	30	0.7508
	3: Pathogen vs. 5: Nd-1-1	−5.246	−10.78 to 0.2901	1.502	4.938	30	0.0773
	3: Pathogen vs. 5: Pb-2-2	0.4653	−5.071 to 6.001	1.502	0.438	30	>0.9999
	3: Pathogen vs. 7: Blank	−14	−19.54 to −8.468	1.502	13.18	30	<0.0001
	3: Pathogen vs. 7: Pathogen	4.56	−0.9761 to 10.10	1.502	4.293	30	0.197
	3: Pathogen vs. 7: N2-1-1	0.8605	−4.676 to 6.396	1.502	0.81	30	>0.9999
	3: Pathogen vs. 7: Nd-1-1	−0.063	−5.599 to 5.473	1.502	0.05931	30	>0.9999
	3: Pathogen vs. 7: Pb-2-2	2.219	−3.317 to 7.755	1.502	2.089	30	0.9704
	3: N2-1-1 vs. 3: Nd-1-1	−1.356	−6.892 to 4.180	1.502	1.276	30	0.9998
	3: N2-1-1 vs. 3: Pb-2-2	2.561	−2.975 to 8.097	1.502	2.411	30	0.9155
	3: N2-1-1 vs. 5: Blank	−1.271	−6.807 to 4.265	1.502	1.196	30	0.9999
	3: N2-1-1 vs. 5: Pathogen	6.549	1.013 to 12.08	1.502	6.165	30	0.0096
	3: N2-1-1 vs. 5: N2-1-1	1.734	−3.802 to 7.270	1.502	1.632	30	0.9968
	3: N2-1-1 vs. 5: Nd-1-1	−0.4224	−5.958 to 5.114	1.502	0.3976	30	>0.9999
	3: N2-1-1 vs. 5: Pb-2-2	5.289	−0.2472 to 10.82	1.502	4.979	30	0.0726
	3: N2-1-1 vs. 7: Blank	−9.18	−14.72 to −3.644	1.502	8.642	30	<0.0001
	3: N2-1-1 vs. 7: Pathogen	9.383	3.847 to 14.92	1.502	8.833	30	<0.0001
	3: N2-1-1 vs. 7: N2-1-1	5.684	0.1480 to 11.22	1.502	5.351	30	0.0397
	3: N2-1-1 vs. 7: Nd-1-1	4.761	−0.7755 to 10.30	1.502	4.481	30	0.1523
	3: N2-1-1 vs. 7: Pb-2-2	7.042	1.506 to 12.58	1.502	6.629	30	0.0041
	3: Nd-1-1 vs. 3: Pb-2-2	3.916	−1.620 to 9.452	1.502	3.687	30	0.404
	3: Nd-1-1 vs. 5: Blank	0.08477	−5.451 to 5.621	1.502	0.0798	30	>0.9999
	3: Nd-1-1 vs. 5: Pathogen	7.904	2.368 to 13.44	1.502	7.441	30	0.0009
	3: Nd-1-1 vs. 5: N2-1-1	3.089	−2.447 to 8.625	1.502	2.908	30	0.7511
	3: Nd-1-1 vs. 5: Nd-1-1	0.9332	−4.603 to 6.469	1.502	0.8785	30	>0.9999
	3: Nd-1-1 vs. 5: Pb-2-2	6.644	1.108 to 12.18	1.502	6.255	30	0.0082
	3: Nd-1-1 vs. 7: Blank	−7.825	−13.36 to −2.289	1.502	7.366	30	0.001
	3: Nd-1-1 vs. 7: Pathogen	10.74	5.203 to 16.27	1.502	10.11	30	<0.0001
	3: Nd-1-1 vs. 7: N2-1-1	7.04	1.504 to 12.58	1.502	6.627	30	0.0041
	3: Nd-1-1 vs. 7: Nd-1-1	6.116	0.5801 to 11.65	1.502	5.757	30	0.0198
	3: Nd-1-1 vs. 7: Pb-2-2	8.398	2.862 to 13.93	1.502	7.906	30	0.0004
	3: Pb-2-2 vs. 5: Blank	−3.832	−9.367 to 1.704	1.502	3.607	30	0.4378
	3: Pb-2-2 vs. 5: Pathogen	3.988	−1.548 to 9.524	1.502	3.754	30	0.3764
	3: Pb-2-2 vs. 5: N2-1-1	−0.8271	−6.363 to 4.709	1.502	0.7786	30	>0.9999
	3: Pb-2-2 vs. 5: Nd-1-1	−2.983	−8.519 to 2.553	1.502	2.808	30	0.791
	3: Pb-2-2 vs. 5: Pb-2-2	2.728	−2.808 to 8.264	1.502	2.568	30	0.8736
	3: Pb-2-2 vs. 7: Blank	−11.74	−17.28 to −6.205	1.502	11.05	30	<0.0001
	3: Pb-2-2 vs. 7: Pathogen	6.823	1.287 to 12.36	1.502	6.423	30	0.006
	3: Pb-2-2 vs. 7: N2-1-1	3.123	−2.413 to 8.659	1.502	2.94	30	0.7377
	3: Pb-2-2 vs. 7: Nd-1-1	2.2	−3.336 to 7.736	1.502	2.071	30	0.9724
	3: Pb-2-2 vs. 7: Pb-2-2	4.482	−1.054 to 10.02	1.502	4.219	30	0.217
	5: Blank vs. 5: Pathogen	7.819	2.283 to 13.36	1.502	7.361	30	0.001
	5: Blank vs. 5: N2-1-1	3.004	−2.532 to 8.540	1.502	2.828	30	0.7832
	5: Blank vs. 5: Nd-1-1	0.8484	−4.688 to 6.384	1.502	0.7987	30	>0.9999
	5: Blank vs. 5: Pb-2-2	6.56	1.024 to 12.10	1.502	6.175	30	0.0094
	5: Blank vs. 7: Blank	−7.91	−13.45 to −2.374	1.502	7.446	30	0.0009
	5: Blank vs. 7: Pathogen	10.65	5.118 to 16.19	1.502	10.03	30	<0.0001
	5: Blank vs. 7: N2-1-1	6.955	1.419 to 12.49	1.502	6.547	30	0.0048
	5: Blank vs. 7: Nd-1-1	6.031	0.4953 to 11.57	1.502	5.678	30	0.0228
	5: Blank vs. 7: Pb-2-2	8.313	2.777 to 13.85	1.502	7.826	30	0.0004
	5: Pathogen vs. 5: N2-1-1	−4.815	−10.35 to 0.7211	1.502	4.533	30	0.1416
	5: Pathogen vs. 5: Nd-1-1	−6.971	−12.51 to −1.435	1.502	6.562	30	0.0046
	5: Pathogen vs. 5: Pb-2-2	−1.26	−6.796 to 4.276	1.502	1.186	30	0.9999
	5: Pathogen vs. 7: Blank	−15.73	−21.26 to −10.19	1.502	14.81	30	<0.0001
	5: Pathogen vs. 7: Pathogen	2.835	−2.701 to 8.371	1.502	2.669	30	0.8416
	5: Pathogen vs. 7: N2-1-1	−0.8646	−6.401 to 4.671	1.502	0.8139	30	>0.9999
	5: Pathogen vs. 7: Nd-1-1	−1.788	−7.324 to 3.748	1.502	1.683	30	0.9956
	5: Pathogen vs. 7: Pb-2-2	0.4938	−5.042 to 6.030	1.502	0.4649	30	>0.9999
	5: N2-1-1 vs. 5: Nd-1-1	−2.156	−7.692 to 3.380	1.502	2.03	30	0.9766
	5: N2-1-1 vs. 5: Pb-2-2	3.555	−1.981 to 9.091	1.502	3.347	30	0.5545
	5: N2-1-1 vs. 7: Blank	−10.91	−16.45 to −5.378	1.502	10.27	30	<0.0001
	5: N2-1-1 vs. 7: Pathogen	7.65	2.114 to 13.19	1.502	7.201	30	0.0014
	5: N2-1-1 vs. 7: N2-1-1	3.95	−1.586 to 9.486	1.502	3.719	30	0.3907
	5: N2-1-1 vs. 7: Nd-1-1	3.027	−2.509 to 8.563	1.502	2.849	30	0.7749
	5: N2-1-1 vs. 7: Pb-2-2	5.309	−0.2272 to 10.84	1.502	4.997	30	0.0705
	5: Nd-1-1 vs. 5: Pb-2-2	5.711	0.1752 to 11.25	1.502	5.376	30	0.0381
	5: Nd-1-1 vs. 7: Blank	−8.758	−14.29 to −3.222	1.502	8.244	30	0.0002
	5: Nd-1-1 vs. 7: Pathogen	9.806	4.270 to 15.34	1.502	9.231	30	<0.0001
	5: Nd-1-1 vs. 7: N2-1-1	6.106	0.5704 to 11.64	1.502	5.748	30	0.0202
	5: Nd-1-1 vs. 7: Nd-1-1	5.183	−0.3531 to 10.72	1.502	4.879	30	0.0847
	5: Nd-1-1 vs. 7: Pb-2-2	7.465	1.929 to 13.00	1.502	7.027	30	0.0019
	5: Pb-2-2 vs. 7: Blank	−14.47	−20.01 to −8.933	1.502	13.62	30	<0.0001
	5: Pb-2-2 vs. 7: Pathogen	4.095	−1.441 to 9.631	1.502	3.855	30	0.3372
	5: Pb-2-2 vs. 7: N2-1-1	0.3952	−5.141 to 5.931	1.502	0.372	30	>0.9999
	5: Pb-2-2 vs. 7: Nd-1-1	−0.5283	−6.064 to 5.008	1.502	0.4973	30	>0.9999
	5: Pb-2-2 vs. 7: Pb-2-2	1.754	−3.782 to 7.290	1.502	1.651	30	0.9964
	7: Blank vs. 7: Pathogen	18.56	13.03 to 24.10	1.502	17.48	30	<0.0001
	7: Blank vs. 7: N2-1-1	14.86	9.328 to 20.40	1.502	13.99	30	<0.0001
	7: Blank vs. 7: Nd-1-1	13.94	8.405 to 19.48	1.502	13.12	30	<0.0001
	7: Blank vs. 7: Pb-2-2	16.22	10.69 to 21.76	1.502	15.27	30	<0.0001
	7: Pathogen vs. 7: N2-1-1	−3.699	−9.235 to 1.837	1.502	3.483	30	0.4926
	7: Pathogen vs. 7: Nd-1-1	−4.623	−10.16 to 0.9131	1.502	4.352	30	0.182
	7: Pathogen vs. 7: Pb-2-2	−2.341	−7.877 to 3.195	1.502	2.204	30	0.9553
	7: N2-1-1 vs. 7: Nd-1-1	−0.9235	−6.459 to 4.613	1.502	0.8693	30	>0.9999
	7: N2-1-1 vs. 7: Pb-2-2	1.358	−4.178 to 6.894	1.502	1.279	30	0.9998
	7: Nd-1-1 vs. 7: Pb-2-2	2.282	−3.254 to 7.818	1.502	2.148	30	0.9632

Note: CAT, catalase. The comparisons are denoted as “time point:treatment”, where time points “3”, “5”, and “7” indicate days post-inoculation; “Pathogen” refers to the original pathogen isolate; “N2-1-1”, “Nd-1-1”, and “Pb-2-2” are distinct microbial strains/isolates; “Blank” represents the non-inoculated control. For each pairwise comparison, the mean difference, 95% confidence interval (CI) of the difference, standard error of the difference (SE), studentized range statistic (q), degrees of freedom (df), and adjusted *p*-value (Tukey’s method) are presented. Due to a balanced design and homogeneity of variance, the SE of the difference (1.502) and the degrees of freedom (30) are constant across all comparisons. The critical significance level was set at α = 0.05, and adjusted *p*-values were computed to control the family-wise error rate.

**Table A8:** Tukey’s multiple comparisons test for APX enzyme activity.

Pathogen	Comparison	Mean Difference	95.00% CI	SE of Difference	q	*df*	Adjusted *p*
*Alternaria*	3: Blank vs. 3: Pathogen	20.14	17.99 to 22.28	0.5814	48.98	30	<0.0001
	3: Blank vs. 3: N2-1-1	12.36	10.21 to 14.50	0.5814	30.05	30	<0.0001
	3: Blank vs. 3: Nd-1-1	10.63	8.490 to 12.78	0.5814	25.86	30	<0.0001
	3: Blank vs. 3: Pb-2-2	12.59	10.45 to 14.73	0.5814	30.62	30	<0.0001
	3: Blank vs. 5: Blank	7.373	5.230 to 9.515	0.5814	17.93	30	<0.0001
	3: Blank vs. 5: Pathogen	22.24	20.10 to 24.38	0.5814	54.09	30	<0.0001
	3: Blank vs. 5: N2-1-1	14.8	12.66 to 16.94	0.5814	35.99	30	<0.0001
	3: Blank vs. 5: Nd-1-1	16.81	14.67 to 18.96	0.5814	40.9	30	<0.0001
	3: Blank vs. 5: Pb-2-2	15.69	13.55 to 17.84	0.5814	38.17	30	<0.0001
	3: Blank vs. 7: Blank	9.785	7.642 to 11.93	0.5814	23.8	30	<0.0001
	3: Blank vs. 7: Pathogen	23.65	21.50 to 25.79	0.5814	57.51	30	<0.0001
	3: Blank vs. 7: N2-1-1	20.46	18.32 to 22.60	0.5814	49.76	30	<0.0001
	3: Blank vs. 7: Nd-1-1	19.43	17.29 to 21.58	0.5814	47.27	30	<0.0001
	3: Blank vs. 7: Pb-2-2	19.44	17.29 to 21.58	0.5814	47.27	30	<0.0001
	3: Pathogen vs. 3: N2-1-1	−7.779	−9.922 to −5.636	0.5814	18.92	30	<0.0001
	3: Pathogen vs. 3: Nd-1-1	−9.503	−11.65 to −7.361	0.5814	23.11	30	<0.0001
	3: Pathogen vs. 3: Pb-2-2	−7.547	−9.689 to −5.404	0.5814	18.36	30	<0.0001
	3: Pathogen vs. 5: Blank	−12.76	−14.91 to −10.62	0.5814	31.04	30	<0.0001
	3: Pathogen vs. 5: Pathogen	2.105	−0.03808 to 4.247	0.5814	5.119	30	0.0581
	3: Pathogen vs. 5: N2-1-1	−5.338	−7.481 to −3.196	0.5814	12.98	30	<0.0001
	3: Pathogen vs. 5: Nd-1-1	−3.322	−5.465 to −1.179	0.5814	8.079	30	0.0003
	3: Pathogen vs. 5: Pb-2-2	−4.442	−6.585 to −2.300	0.5814	10.8	30	<0.0001
	3: Pathogen vs. 7: Blank	−10.35	−12.49 to −8.209	0.5814	25.18	30	<0.0001
	3: Pathogen vs. 7: Pathogen	3.511	1.368 to 5.654	0.5814	8.54	30	0.0001
	3: Pathogen vs. 7: N2-1-1	0.3246	−1.818 to 2.467	0.5814	0.7896	30	>0.9999
	3: Pathogen vs. 7: Nd-1-1	−0.7031	−2.846 to 1.440	0.5814	1.71	30	0.9949
	3: Pathogen vs. 7: Pb-2-2	−0.6993	−2.842 to 1.443	0.5814	1.701	30	0.9951
	3: N2-1-1 vs. 3: Nd-1-1	−1.724	−3.867 to 0.4186	0.5814	4.193	30	0.2243
	3: N2-1-1 vs. 3: Pb-2-2	0.2323	−1.910 to 2.375	0.5814	0.5649	30	>0.9999
	3: N2-1-1 vs. 5: Blank	−4.984	−7.127 to −2.842	0.5814	12.12	30	<0.0001
	3: N2-1-1 vs. 5: Pathogen	9.884	7.741 to 12.03	0.5814	24.04	30	<0.0001
	3: N2-1-1 vs. 5: N2-1-1	2.441	0.2983 to 4.584	0.5814	5.937	30	0.0144
	3: N2-1-1 vs. 5: Nd-1-1	4.457	2.315 to 6.600	0.5814	10.84	30	<0.0001
	3: N2-1-1 vs. 5: Pb-2-2	3.337	1.194 to 5.479	0.5814	8.116	30	0.0002
	3: N2-1-1 vs. 7: Blank	−2.572	−4.715 to −0.4295	0.5814	6.256	30	0.0081
	3: N2-1-1 vs. 7: Pathogen	11.29	9.147 to 13.43	0.5814	27.46	30	<0.0001
	3: N2-1-1 vs. 7: N2-1-1	8.104	5.961 to 10.25	0.5814	19.71	30	<0.0001
	3: N2-1-1 vs. 7: Nd-1-1	7.076	4.933 to 9.219	0.5814	17.21	30	<0.0001
	3: N2-1-1 vs. 7: Pb-2-2	7.08	4.937 to 9.222	0.5814	17.22	30	<0.0001
	3: Nd-1-1 vs. 3: Pb-2-2	1.956	−0.1863 to 4.099	0.5814	4.758	30	0.1018
	3: Nd-1-1 vs. 5: Blank	−3.26	−5.403 to −1.118	0.5814	7.929	30	0.0003
	3: Nd-1-1 vs. 5: Pathogen	11.61	9.465 to 13.75	0.5814	28.23	30	<0.0001
	3: Nd-1-1 vs. 5: N2-1-1	4.165	2.022 to 6.308	0.5814	10.13	30	<0.0001
	3: Nd-1-1 vs. 5: Nd-1-1	6.181	4.039 to 8.324	0.5814	15.03	30	<0.0001
	3: Nd-1-1 vs. 5: Pb-2-2	5.061	2.918 to 7.204	0.5814	12.31	30	<0.0001
	3: Nd-1-1 vs. 7: Blank	−0.8481	−2.991 to 1.295	0.5814	2.063	30	0.9733
	3: Nd-1-1 vs. 7: Pathogen	13.01	10.87 to 15.16	0.5814	31.65	30	<0.0001
	3: Nd-1-1 vs. 7: N2-1-1	9.828	7.685 to 11.97	0.5814	23.9	30	<0.0001
	3: Nd-1-1 vs. 7: Nd-1-1	8.8	6.657 to 10.94	0.5814	21.4	30	<0.0001
	3: Nd-1-1 vs. 7: Pb-2-2	8.804	6.661 to 10.95	0.5814	21.41	30	<0.0001
	3: Pb-2-2 vs. 5: Blank	−5.217	−7.359 to −3.074	0.5814	12.69	30	<0.0001
	3: Pb-2-2 vs. 5: Pathogen	9.651	7.509 to 11.79	0.5814	23.47	30	<0.0001
	3: Pb-2-2 vs. 5: N2-1-1	2.209	0.06606 to 4.351	0.5814	5.372	30	0.0383
	3: Pb-2-2 vs. 5: Nd-1-1	4.225	2.082 to 6.368	0.5814	10.28	30	<0.0001
	3: Pb-2-2 vs. 5: Pb-2-2	3.105	0.9619 to 5.247	0.5814	7.551	30	0.0007
	3: Pb-2-2 vs. 7: Blank	−2.804	−4.947 to −0.6618	0.5814	6.821	30	0.0029
	3: Pb-2-2 vs. 7: Pathogen	11.06	8.915 to 13.20	0.5814	26.9	30	<0.0001
	3: Pb-2-2 vs. 7: N2-1-1	7.871	5.729 to 10.01	0.5814	19.15	30	<0.0001
	3: Pb-2-2 vs. 7: Nd-1-1	6.844	4.701 to 8.986	0.5814	16.65	30	<0.0001
	3: Pb-2-2 vs. 7: Pb-2-2	6.848	4.705 to 8.990	0.5814	16.65	30	<0.0001
	5: Blank vs. 5: Pathogen	14.87	12.73 to 17.01	0.5814	36.16	30	<0.0001
	5: Blank vs. 5: N2-1-1	7.425	5.283 to 9.568	0.5814	18.06	30	<0.0001
	5: Blank vs. 5: Nd-1-1	9.442	7.299 to 11.58	0.5814	22.96	30	<0.0001
	5: Blank vs. 5: Pb-2-2	8.321	6.178 to 10.46	0.5814	20.24	30	<0.0001
	5: Blank vs. 7: Blank	2.412	0.2694 to 4.555	0.5814	5.867	30	0.0164
	5: Blank vs. 7: Pathogen	16.27	14.13 to 18.42	0.5814	39.58	30	<0.0001
	5: Blank vs. 7: N2-1-1	13.09	10.95 to 15.23	0.5814	31.83	30	<0.0001
	5: Blank vs. 7: Nd-1-1	12.06	9.918 to 14.20	0.5814	29.33	30	<0.0001
	5: Blank vs. 7: Pb-2-2	12.06	9.921 to 14.21	0.5814	29.34	30	<0.0001
	5: Pathogen vs. 5: N2-1-1	−7.443	−9.585 to −5.300	0.5814	18.1	30	<0.0001
	5: Pathogen vs. 5: Nd-1-1	−5.426	−7.569 to −3.284	0.5814	13.2	30	<0.0001
	5: Pathogen vs. 5: Pb-2-2	−6.547	−8.690 to −4.404	0.5814	15.92	30	<0.0001
	5: Pathogen vs. 7: Blank	−12.46	−14.60 to −10.31	0.5814	30.3	30	<0.0001
	5: Pathogen vs. 7: Pathogen	1.406	−0.7362 to 3.549	0.5814	3.421	30	0.5206
	5: Pathogen vs. 7: N2-1-1	−1.78	−3.923 to 0.3627	0.5814	4.329	30	0.1876
	5: Pathogen vs. 7: Nd-1-1	−2.808	−4.950 to −0.6650	0.5814	6.829	30	0.0028
	5: Pathogen vs. 7: Pb-2-2	−2.804	−4.947 to −0.6613	0.5814	6.82	30	0.0029
	5: N2-1-1 vs. 5: Nd-1-1	2.016	−0.1264 to 4.159	0.5814	4.904	30	0.0815
	5: N2-1-1 vs. 5: Pb-2-2	0.8958	−1.247 to 3.038	0.5814	2.179	30	0.9589
	5: N2-1-1 vs. 7: Blank	−5.013	−7.156 to −2.870	0.5814	12.19	30	<0.0001
	5: N2-1-1 vs. 7: Pathogen	8.849	6.706 to 10.99	0.5814	21.52	30	<0.0001
	5: N2-1-1 vs. 7: N2-1-1	5.663	3.520 to 7.805	0.5814	13.77	30	<0.0001
	5: N2-1-1 vs. 7: Nd-1-1	4.635	2.492 to 6.778	0.5814	11.27	30	<0.0001
	5: N2-1-1 vs. 7: Pb-2-2	4.639	2.496 to 6.781	0.5814	11.28	30	<0.0001
	5: Nd-1-1 vs. 5: Pb-2-2	−1.12	−3.263 to 1.022	0.5814	2.725	30	0.8219
	5: Nd-1-1 vs. 7: Blank	−7.029	−9.172 to −4.887	0.5814	17.1	30	<0.0001
	5: Nd-1-1 vs. 7: Pathogen	6.833	4.690 to 8.975	0.5814	16.62	30	<0.0001
	5: Nd-1-1 vs. 7: N2-1-1	3.646	1.504 to 5.789	0.5814	8.869	30	<0.0001
	5: Nd-1-1 vs. 7: Nd-1-1	2.619	0.4761 to 4.761	0.5814	6.369	30	0.0066
	5: Nd-1-1 vs. 7: Pb-2-2	2.623	0.4799 to 4.765	0.5814	6.379	30	0.0065
	5: Pb-2-2 vs. 7: Blank	−5.909	−8.052 to −3.766	0.5814	14.37	30	<0.0001
	5: Pb-2-2 vs. 7: Pathogen	7.953	5.811 to 10.10	0.5814	19.34	30	<0.0001
	5: Pb-2-2 vs. 7: N2-1-1	4.767	2.624 to 6.910	0.5814	11.59	30	<0.0001
	5: Pb-2-2 vs. 7: Nd-1-1	3.739	1.597 to 5.882	0.5814	9.095	30	<0.0001
	5: Pb-2-2 vs. 7: Pb-2-2	3.743	1.600 to 5.886	0.5814	9.104	30	<0.0001
	7: Blank vs. 7: Pathogen	13.86	11.72 to 16.00	0.5814	33.72	30	<0.0001
	7: Blank vs. 7: N2-1-1	10.68	8.533 to 12.82	0.5814	25.97	30	<0.0001
	7: Blank vs. 7: Nd-1-1	9.648	7.506 to 11.79	0.5814	23.47	30	<0.0001
	7: Blank vs. 7: Pb-2-2	9.652	7.509 to 11.79	0.5814	23.48	30	<0.0001
	7: Pathogen vs. 7: N2-1-1	−3.186	−5.329 to −1.044	0.5814	7.75	30	0.0005
	7: Pathogen vs. 7: Nd-1-1	−4.214	−6.357 to −2.071	0.5814	10.25	30	<0.0001
*Fusarium*	7: Pathogen vs. 7: Pb-2-2	−4.21	−6.353 to −2.068	0.5814	10.24	30	<0.0001
	7: N2-1-1 vs. 7: Nd-1-1	−1.028	−3.170 to 1.115	0.5814	2.5	30	0.8931
	7: N2-1-1 vs. 7: Pb-2-2	−1.024	−3.167 to 1.119	0.5814	2.491	30	0.8955
	7: Nd-1-1 vs. 7: Pb-2-2	0.003745	−2.139 to 2.146	0.5814	0.009108	30	>0.9999
	3: Blank vs. 3: Pathogen	20.34	17.36 to 23.32	0.8095	35.54	30	<0.0001
	3: Blank vs. 3: N2-1-1	12.45	9.471 to 15.44	0.8095	21.76	30	<0.0001
	3: Blank vs. 3: Nd-1-1	12.72	9.741 to 15.71	0.8095	22.23	30	<0.0001
	3: Blank vs. 3: Pb-2-2	11.4	8.421 to 14.39	0.8095	19.92	30	<0.0001
	3: Blank vs. 5: Blank	7.373	4.390 to 10.36	0.8095	12.88	30	<0.0001
	3: Blank vs. 5: Pathogen	21.79	18.81 to 24.78	0.8095	38.08	30	<0.0001
	3: Blank vs. 5: N2-1-1	15	12.02 to 17.99	0.8095	26.21	30	<0.0001
	3: Blank vs. 5: Nd-1-1	16.03	13.05 to 19.02	0.8095	28.01	30	<0.0001
	3: Blank vs. 5: Pb-2-2	16.26	13.28 to 19.24	0.8095	28.41	30	<0.0001
	3: Blank vs. 7: Blank	9.785	6.802 to 12.77	0.8095	17.09	30	<0.0001
	3: Blank vs. 7: Pathogen	23.14	20.16 to 26.12	0.8095	40.43	30	<0.0001
	3: Blank vs. 7: N2-1-1	19.53	16.55 to 22.51	0.8095	34.12	30	<0.0001
	3: Blank vs. 7: Nd-1-1	19.38	16.39 to 22.36	0.8095	33.85	30	<0.0001
	3: Blank vs. 7: Pb-2-2	18.93	15.94 to 21.91	0.8095	33.07	30	<0.0001
	3: Pathogen vs. 3: N2-1-1	−7.887	−10.87 to −4.904	0.8095	13.78	30	<0.0001
	3: Pathogen vs. 3: Nd-1-1	−7.617	−10.60 to −4.634	0.8095	13.31	30	<0.0001
	3: Pathogen vs. 3: Pb-2-2	−8.937	−11.92 to −5.954	0.8095	15.61	30	<0.0001
	3: Pathogen vs. 5: Blank	−12.97	−15.95 to −9.986	0.8095	22.66	30	<0.0001
	3: Pathogen vs. 5: Pathogen	1.453	−1.530 to 4.436	0.8095	2.538	30	0.8823
	3: Pathogen vs. 5: N2-1-1	−5.339	−8.321 to −2.356	0.8095	9.327	30	<0.0001
	3: Pathogen vs. 5: Nd-1-1	−4.308	−7.290 to −1.325	0.8095	7.526	30	0.0008
	3: Pathogen vs. 5: Pb-2-2	−4.081	−7.064 to −1.098	0.8095	7.13	30	0.0016
	3: Pathogen vs. 7: Blank	−10.56	−13.54 to −7.574	0.8095	18.44	30	<0.0001
	3: Pathogen vs. 7: Pathogen	2.798	−0.1852 to 5.781	0.8095	4.888	30	0.0836
	3: Pathogen vs. 7: N2-1-1	−0.8127	−3.796 to 2.170	0.8095	1.42	30	0.9992
	3: Pathogen vs. 7: Nd-1-1	−0.9644	−3.947 to 2.018	0.8095	1.685	30	0.9956
	3: Pathogen vs. 7: Pb-2-2	−1.414	−4.396 to 1.569	0.8095	2.47	30	0.901
	3: N2-1-1 vs. 3: Nd-1-1	0.27	−2.713 to 3.253	0.8095	0.4717	30	>0.9999
	3: N2-1-1 vs. 3: Pb-2-2	−1.05	−4.033 to 1.933	0.8095	1.835	30	0.9902
	3: N2-1-1 vs. 5: Blank	−5.082	−8.065 to −2.099	0.8095	8.878	30	<0.0001
	3: N2-1-1 vs. 5: Pathogen	9.34	6.357 to 12.32	0.8095	16.32	30	<0.0001
	3: N2-1-1 vs. 5: N2-1-1	2.548	−0.4347 to 5.531	0.8095	4.452	30	0.1586
	3: N2-1-1 vs. 5: Nd-1-1	3.579	0.5964 to 6.562	0.8095	6.253	30	0.0082
	3: N2-1-1 vs. 5: Pb-2-2	3.806	0.8227 to 6.788	0.8095	6.649	30	0.0039
	3: N2-1-1 vs. 7: Blank	−2.67	−5.653 to 0.3132	0.8095	4.664	30	0.1171
	3: N2-1-1 vs. 7: Pathogen	10.68	7.702 to 13.67	0.8095	18.67	30	<0.0001
	3: N2-1-1 vs. 7: N2-1-1	7.074	4.091 to 10.06	0.8095	12.36	30	<0.0001
	3: N2-1-1 vs. 7: Nd-1-1	6.922	3.939 to 9.905	0.8095	12.09	30	<0.0001
	3: N2-1-1 vs. 7: Pb-2-2	6.473	3.490 to 9.456	0.8095	11.31	30	<0.0001
	3: Nd-1-1 vs. 3: Pb-2-2	−1.32	−4.303 to 1.663	0.8095	2.307	30	0.9376
	3: Nd-1-1 vs. 5: Blank	−5.352	−8.335 to −2.369	0.8095	9.35	30	<0.0001
	3: Nd-1-1 vs. 5: Pathogen	9.07	6.087 to 12.05	0.8095	15.85	30	<0.0001
	3: Nd-1-1 vs. 5: N2-1-1	2.278	−0.7047 to 5.261	0.8095	3.98	30	0.2918
	3: Nd-1-1 vs. 5: Nd-1-1	3.309	0.3263 to 6.292	0.8095	5.782	30	0.019
	3: Nd-1-1 vs. 5: Pb-2-2	3.536	0.5527 to 6.518	0.8095	6.177	30	0.0094
	3: Nd-1-1 vs. 7: Blank	−2.94	−5.923 to 0.04324	0.8095	5.136	30	0.0565
	3: Nd-1-1 vs. 7: Pathogen	10.41	7.432 to 13.40	0.8095	18.19	30	<0.0001
	3: Nd-1-1 vs. 7: N2-1-1	6.804	3.821 to 9.787	0.8095	11.89	30	<0.0001
	3: Nd-1-1 vs. 7: Nd-1-1	6.652	3.669 to 9.635	0.8095	11.62	30	<0.0001
	3: Nd-1-1 vs. 7: Pb-2-2	6.203	3.220 to 9.186	0.8095	10.84	30	<0.0001
	3: Pb-2-2 vs. 5: Blank	−4.031	−7.014 to −1.048	0.8095	7.043	30	0.0019
	3: Pb-2-2 vs. 5: Pathogen	10.39	7.407 to 13.37	0.8095	18.15	30	<0.0001
	3: Pb-2-2 vs. 5: N2-1-1	3.599	0.6157 to 6.581	0.8095	6.287	30	0.0077
	3: Pb-2-2 vs. 5: Nd-1-1	4.63	1.647 to 7.612	0.8095	8.088	30	0.0003
	3: Pb-2-2 vs. 5: Pb-2-2	4.856	1.873 to 7.839	0.8095	8.484	30	0.0001
	3: Pb-2-2 vs. 7: Blank	−1.619	−4.602 to 1.364	0.8095	2.829	30	0.7829
	3: Pb-2-2 vs. 7: Pathogen	11.73	8.752 to 14.72	0.8095	20.5	30	<0.0001
	3: Pb-2-2 vs. 7: N2-1-1	8.124	5.142 to 11.11	0.8095	14.19	30	<0.0001
	3: Pb-2-2 vs. 7: Nd-1-1	7.973	4.990 to 10.96	0.8095	13.93	30	<0.0001
	3: Pb-2-2 vs. 7: Pb-2-2	7.524	4.541 to 10.51	0.8095	13.14	30	<0.0001
	5: Blank vs. 5: Pathogen	14.42	11.44 to 17.40	0.8095	25.2	30	<0.0001
	5: Blank vs. 5: N2-1-1	7.63	4.647 to 10.61	0.8095	13.33	30	<0.0001
	5: Blank vs. 5: Nd-1-1	8.661	5.678 to 11.64	0.8095	15.13	30	<0.0001
	5: Blank vs. 5: Pb-2-2	8.887	5.904 to 11.87	0.8095	15.53	30	<0.0001
	5: Blank vs. 7: Blank	2.412	−0.5708 to 5.395	0.8095	4.214	30	0.2183
	5: Blank vs. 7: Pathogen	15.77	12.78 to 18.75	0.8095	27.54	30	<0.0001
	5: Blank vs. 7: N2-1-1	12.16	9.173 to 15.14	0.8095	21.24	30	<0.0001
	5: Blank vs. 7: Nd-1-1	12	9.021 to 14.99	0.8095	20.97	30	<0.0001
	5: Blank vs. 7: Pb-2-2	11.55	8.572 to 14.54	0.8095	20.19	30	<0.0001
	5: Pathogen vs. 5: N2-1-1	−6.791	−9.774 to −3.808	0.8095	11.87	30	<0.0001
	5: Pathogen vs. 5: Nd-1-1	−5.76	−8.743 to −2.777	0.8095	10.06	30	<0.0001
	5: Pathogen vs. 5: Pb-2-2	−5.534	−8.517 to −2.551	0.8095	9.668	30	<0.0001
	5: Pathogen vs. 7: Blank	−12.01	−14.99 to −9.026	0.8095	20.98	30	<0.0001
	5: Pathogen vs. 7: Pathogen	1.345	−1.638 to 4.328	0.8095	2.349	30	0.929
	5: Pathogen vs. 7: N2-1-1	−2.266	−5.248 to 0.7174	0.8095	3.958	30	0.2995
	5: Pathogen vs. 7: Nd-1-1	−2.417	−5.400 to 0.5656	0.8095	4.223	30	0.2158
	5: Pathogen vs. 7: Pb-2-2	−2.866	−5.849 to 0.1165	0.8095	5.008	30	0.0693
	5: N2-1-1 vs. 5: Nd-1-1	1.031	−1.952 to 4.014	0.8095	1.801	30	0.9917
	5: N2-1-1 vs. 5: Pb-2-2	1.257	−1.726 to 4.240	0.8095	2.197	30	0.9563
	5: N2-1-1 vs. 7: Blank	−5.218	−8.201 to −2.235	0.8095	9.116	30	<0.0001
	5: N2-1-1 vs. 7: Pathogen	8.136	5.153 to 11.12	0.8095	14.21	30	<0.0001
	5: N2-1-1 vs. 7: N2-1-1	4.526	1.543 to 7.509	0.8095	7.907	30	0.0004
	5: N2-1-1 vs. 7: Nd-1-1	4.374	1.391 to 7.357	0.8095	7.642	30	0.0006
	5: N2-1-1 vs. 7: Pb-2-2	3.925	0.9421 to 6.908	0.8095	6.857	30	0.0027
	5: Nd-1-1 vs. 5: Pb-2-2	0.2263	−2.757 to 3.209	0.8095	0.3954	30	>0.9999
	5: Nd-1-1 vs. 7: Blank	−6.249	−9.232 to −3.266	0.8095	10.92	30	<0.0001
	5: Nd-1-1 vs. 7: Pathogen	7.105	4.122 to 10.09	0.8095	12.41	30	<0.0001
	5: Nd-1-1 vs. 7: N2-1-1	3.495	0.5119 to 6.478	0.8095	6.106	30	0.0107
	5: Nd-1-1 vs. 7: Nd-1-1	3.343	0.3602 to 6.326	0.8095	5.841	30	0.0171
	5: Nd-1-1 vs. 7: Pb-2-2	2.894	−0.08896 to 5.877	0.8095	5.056	30	0.0642
	5: Pb-2-2 vs. 7: Blank	−6.475	−9.458 to −3.492	0.8095	11.31	30	<0.0001
	5: Pb-2-2 vs. 7: Pathogen	6.879	3.896 to 9.862	0.8095	12.02	30	<0.0001
	5: Pb-2-2 vs. 7: N2-1-1	3.269	0.2856 to 6.251	0.8095	5.71	30	0.0215
	5: Pb-2-2 vs. 7: Nd-1-1	3.117	0.1338 to 6.100	0.8095	5.445	30	0.0339
	5: Pb-2-2 vs. 7: Pb-2-2	2.668	−0.3153 to 5.650	0.8095	4.661	30	0.1177
	7: Blank vs. 7: Pathogen	13.35	10.37 to 16.34	0.8095	23.33	30	<0.0001
	7: Blank vs. 7: N2-1-1	9.744	6.761 to 12.73	0.8095	17.02	30	<0.0001
	7: Blank vs. 7: Nd-1-1	9.592	6.609 to 12.57	0.8095	16.76	30	<0.0001
	7: Blank vs. 7: Pb-2-2	9.143	6.160 to 12.13	0.8095	15.97	30	<0.0001
	7: Pathogen vs. 7: N2-1-1	−3.61	−6.593 to −0.6274	0.8095	6.308	30	0.0074
	7: Pathogen vs. 7: Nd-1-1	−3.762	−6.745 to −0.7792	0.8095	6.573	30	0.0045
	7: Pathogen vs. 7: Pb-2-2	−4.211	−7.194 to −1.228	0.8095	7.357	30	0.001
	7: N2-1-1 vs. 7: Nd-1-1	−0.1518	−3.135 to 2.831	0.8095	0.2652	30	>0.9999
	7: N2-1-1 vs. 7: Pb-2-2	−0.6009	−3.584 to 2.382	0.8095	1.05	30	>0.9999
	7: Nd-1-1 vs. 7: Pb-2-2	−0.4491	−3.432 to 2.534	0.8095	0.7847	30	>0.9999
	3: Blank vs. 3: Pathogen	20.49	17.05 to 23.93	0.9339	31.03	30	<0.0001
	3: Blank vs. 3: N2-1-1	12.73	9.285 to 16.17	0.9339	19.27	30	<0.0001
	3: Blank vs. 3: Nd-1-1	12.76	9.319 to 16.20	0.9339	19.32	30	<0.0001
	3: Blank vs. 3: Pb-2-2	11.92	8.477 to 15.36	0.9339	18.05	30	<0.0001
	3: Blank vs. 5: Blank	7.373	3.931 to 10.81	0.9339	11.16	30	<0.0001
	3: Blank vs. 5: Pathogen	22.66	19.22 to 26.10	0.9339	34.31	30	<0.0001
	3: Blank vs. 5: N2-1-1	16.03	12.58 to 19.47	0.9339	24.27	30	<0.0001
	3: Blank vs. 5: Nd-1-1	16.45	13.01 to 19.89	0.9339	24.91	30	<0.0001
	3: Blank vs. 5: Pb-2-2	16.63	13.19 to 20.07	0.9339	25.19	30	<0.0001
	3: Blank vs. 7: Blank	9.785	6.343 to 13.23	0.9339	14.82	30	<0.0001
	3: Blank vs. 7: Pathogen	22.97	19.53 to 26.42	0.9339	34.79	30	<0.0001
	3: Blank vs. 7: N2-1-1	20.33	16.88 to 23.77	0.9339	30.78	30	<0.0001
	3: Blank vs. 7: Nd-1-1	18.55	15.11 to 22.00	0.9339	28.1	30	<0.0001
	3: Blank vs. 7: Pb-2-2	18.84	15.40 to 22.28	0.9339	28.53	30	<0.0001
	3: Pathogen vs. 3: N2-1-1	−7.765	−11.21 to −4.323	0.9339	11.76	30	<0.0001
	3: Pathogen vs. 3: Nd-1-1	−7.731	−11.17 to −4.289	0.9339	11.71	30	<0.0001
	3: Pathogen vs. 3: Pb-2-2	−8.573	−12.01 to −5.131	0.9339	12.98	30	<0.0001
	3: Pathogen vs. 5: Blank	−13.12	−16.56 to −9.677	0.9339	19.87	30	<0.0001
	3: Pathogen vs. 5: Pathogen	2.168	−1.273 to 5.609	0.9339	3.283	30	0.5839
	3: Pathogen vs. 5: N2-1-1	−4.465	−7.906 to −1.023	0.9339	6.761	30	0.0032
	3: Pathogen vs. 5: Nd-1-1	−4.04	−7.481 to −0.5982	0.9339	6.117	30	0.0105
*Botryosphaeriaceae*	3: Pathogen vs. 5: Pb-2-2	−3.858	−7.300 to −0.4166	0.9339	5.842	30	0.0171
	3: Pathogen vs. 7: Blank	−10.71	−14.15 to −7.265	0.9339	16.21	30	<0.0001
	3: Pathogen vs. 7: Pathogen	2.483	−0.9586 to 5.924	0.9339	3.76	30	0.3741
	3: Pathogen vs. 7: N2-1-1	−0.1653	−3.607 to 3.276	0.9339	0.2503	30	>0.9999
	3: Pathogen vs. 7: Nd-1-1	−1.937	−5.379 to 1.504	0.9339	2.933	30	0.7405
	3: Pathogen vs. 7: Pb-2-2	−1.653	−5.094 to 1.789	0.9339	2.503	30	0.8922
	3: N2-1-1 vs. 3: Nd-1-1	0.03424	−3.407 to 3.476	0.9339	0.05185	30	>0.9999
	3: N2-1-1 vs. 3: Pb-2-2	−0.8078	−4.249 to 2.634	0.9339	1.223	30	0.9999
	3: N2-1-1 vs. 5: Blank	−5.354	−8.795 to −1.912	0.9339	8.107	30	0.0002
	3: N2-1-1 vs. 5: Pathogen	9.933	6.491 to 13.37	0.9339	15.04	30	<0.0001
	3: N2-1-1 vs. 5: N2-1-1	3.3	−0.1414 to 6.742	0.9339	4.997	30	0.0705
	3: N2-1-1 vs. 5: Nd-1-1	3.725	0.2837 to 7.167	0.9339	5.641	30	0.0243
	3: N2-1-1 vs. 5: Pb-2-2	3.907	0.4653 to 7.348	0.9339	5.916	30	0.015
	3: N2-1-1 vs. 7: Blank	−2.942	−6.383 to 0.4999	0.9339	4.454	30	0.1581
	3: N2-1-1 vs. 7: Pathogen	10.25	6.806 to 13.69	0.9339	15.52	30	<0.0001
	3: N2-1-1 vs. 7: N2-1-1	7.6	4.158 to 11.04	0.9339	11.51	30	<0.0001
	3: N2-1-1 vs. 7: Nd-1-1	5.828	2.386 to 9.269	0.9339	8.825	30	<0.0001
	3: N2-1-1 vs. 7: Pb-2-2	6.112	2.671 to 9.554	0.9339	9.255	30	<0.0001
	3: Nd-1-1 vs. 3: Pb-2-2	−0.842	−4.283 to 2.599	0.9339	1.275	30	0.9998
	3: Nd-1-1 vs. 5: Blank	−5.388	−8.829 to −1.946	0.9339	8.159	30	0.0002
	3: Nd-1-1 vs. 5: Pathogen	9.899	6.457 to 13.34	0.9339	14.99	30	<0.0001
	3: Nd-1-1 vs. 5: N2-1-1	3.266	−0.1757 to 6.707	0.9339	4.945	30	0.0765
	3: Nd-1-1 vs. 5: Nd-1-1	3.691	0.2494 to 7.132	0.9339	5.589	30	0.0265
	3: Nd-1-1 vs. 5: Pb-2-2	3.873	0.4310 to 7.314	0.9339	5.864	30	0.0164
	3: Nd-1-1 vs. 7: Blank	−2.976	−6.417 to 0.4656	0.9339	4.506	30	0.147
	3: Nd-1-1 vs. 7: Pathogen	10.21	6.772 to 13.65	0.9339	15.47	30	<0.0001
	3: Nd-1-1 vs. 7: N2-1-1	7.565	4.124 to 11.01	0.9339	11.46	30	<0.0001
	3: Nd-1-1 vs. 7: Nd-1-1	5.793	2.352 to 9.235	0.9339	8.773	30	<0.0001
	3: Nd-1-1 vs. 7: Pb-2-2	6.078	2.636 to 9.519	0.9339	9.204	30	<0.0001
	3: Pb-2-2 vs. 5: Blank	−4.546	−7.987 to −1.104	0.9339	6.884	30	0.0025
	3: Pb-2-2 vs. 5: Pathogen	10.74	7.299 to 14.18	0.9339	16.26	30	<0.0001
	3: Pb-2-2 vs. 5: N2-1-1	4.108	0.6663 to 7.549	0.9339	6.22	30	0.0087
	3: Pb-2-2 vs. 5: Nd-1-1	4.533	1.091 to 7.974	0.9339	6.864	30	0.0026
	3: Pb-2-2 vs. 5: Pb-2-2	4.715	1.273 to 8.156	0.9339	7.139	30	0.0016
	3: Pb-2-2 vs. 7: Blank	−2.134	−5.575 to 1.308	0.9339	3.231	30	0.6078
	3: Pb-2-2 vs. 7: Pathogen	11.06	7.614 to 14.50	0.9339	16.74	30	<0.0001
	3: Pb-2-2 vs. 7: N2-1-1	8.407	4.966 to 11.85	0.9339	12.73	30	<0.0001
	3: Pb-2-2 vs. 7: Nd-1-1	6.635	3.194 to 10.08	0.9339	10.05	30	<0.0001
	3: Pb-2-2 vs. 7: Pb-2-2	6.92	3.478 to 10.36	0.9339	10.48	30	<0.0001
	5: Blank vs. 5: Pathogen	15.29	11.85 to 18.73	0.9339	23.15	30	<0.0001
	5: Blank vs. 5: N2-1-1	8.654	5.212 to 12.10	0.9339	13.1	30	<0.0001
	5: Blank vs. 5: Nd-1-1	9.079	5.637 to 12.52	0.9339	13.75	30	<0.0001
	5: Blank vs. 5: Pb-2-2	9.26	5.819 to 12.70	0.9339	14.02	30	<0.0001
	5: Blank vs. 7: Blank	2.412	−1.029 to 5.854	0.9339	3.653	30	0.4183
	5: Blank vs. 7: Pathogen	15.6	12.16 to 19.04	0.9339	23.62	30	<0.0001
	5: Blank vs. 7: N2-1-1	12.95	9.512 to 16.39	0.9339	19.61	30	<0.0001
	5: Blank vs. 7: Nd-1-1	11.18	7.740 to 14.62	0.9339	16.93	30	<0.0001
	5: Blank vs. 7: Pb-2-2	11.47	8.024 to 14.91	0.9339	17.36	30	<0.0001
	5: Pathogen vs. 5: N2-1-1	−6.633	−10.07 to −3.191	0.9339	10.04	30	<0.0001
	5: Pathogen vs. 5: Nd-1-1	−6.208	−9.649 to −2.766	0.9339	9.4	30	<0.0001
	5: Pathogen vs. 5: Pb-2-2	−6.026	−9.468 to −2.585	0.9339	9.125	30	<0.0001
	5: Pathogen vs. 7: Blank	−12.87	−16.32 to −9.433	0.9339	19.5	30	<0.0001
	5: Pathogen vs. 7: Pathogen	0.3149	−3.127 to 3.756	0.9339	0.4768	30	>0.9999
	5: Pathogen vs. 7: N2-1-1	−2.333	−5.775 to 1.108	0.9339	3.533	30	0.47
	5: Pathogen vs. 7: Nd-1-1	−4.105	−7.547 to −0.6637	0.9339	6.216	30	0.0087
	5: Pathogen vs. 7: Pb-2-2	−3.821	−7.262 to −0.3792	0.9339	5.786	30	0.0189
	5: N2-1-1 vs. 5: Nd-1-1	0.4251	−3.016 to 3.867	0.9339	0.6437	30	>0.9999
	5: N2-1-1 vs. 5: Pb-2-2	0.6067	−2.835 to 4.048	0.9339	0.9187	30	>0.9999
	5: N2-1-1 vs. 7: Blank	−6.242	−9.683 to −2.800	0.9339	9.452	30	<0.0001
	5: N2-1-1 vs. 7: Pathogen	6.948	3.506 to 10.39	0.9339	10.52	30	<0.0001
	5: N2-1-1 vs. 7: N2-1-1	4.3	0.8580 to 7.741	0.9339	6.511	30	0.0051
	5: N2-1-1 vs. 7: Nd-1-1	2.528	−0.9139 to 5.969	0.9339	3.827	30	0.3475
	5: N2-1-1 vs. 7: Pb-2-2	2.812	−0.6295 to 6.254	0.9339	4.258	30	0.2062
	5: Nd-1-1 vs. 5: Pb-2-2	0.1816	−3.260 to 3.623	0.9339	0.275	30	>0.9999
	5: Nd-1-1 vs. 7: Blank	−6.667	−10.11 to −3.225	0.9339	10.1	30	<0.0001
	5: Nd-1-1 vs. 7: Pathogen	6.523	3.081 to 9.964	0.9339	9.877	30	<0.0001
	5: Nd-1-1 vs. 7: N2-1-1	3.874	0.4329 to 7.316	0.9339	5.867	30	0.0164
	5: Nd-1-1 vs. 7: Nd-1-1	2.102	−1.339 to 5.544	0.9339	3.184	30	0.6296
	5: Nd-1-1 vs. 7: Pb-2-2	2.387	−1.055 to 5.828	0.9339	3.615	30	0.4345
	5: Pb-2-2 vs. 7: Blank	−6.848	−10.29 to −3.407	0.9339	10.37	30	<0.0001
	5: Pb-2-2 vs. 7: Pathogen	6.341	2.899 to 9.782	0.9339	9.602	30	<0.0001
	5: Pb-2-2 vs. 7: N2-1-1	3.693	0.2513 to 7.134	0.9339	5.592	30	0.0264
	5: Pb-2-2 vs. 7: Nd-1-1	1.921	−1.521 to 5.362	0.9339	2.909	30	0.7508
	5: Pb-2-2 vs. 7: Pb-2-2	2.205	−1.236 to 5.647	0.9339	3.34	30	0.5579
	7: Blank vs. 7: Pathogen	13.19	9.748 to 16.63	0.9339	19.97	30	<0.0001
	7: Blank vs. 7: N2-1-1	10.54	7.100 to 13.98	0.9339	15.96	30	<0.0001
	7: Blank vs. 7: Nd-1-1	8.769	5.328 to 12.21	0.9339	13.28	30	<0.0001
	7: Blank vs. 7: Pb-2-2	9.054	5.612 to 12.50	0.9339	13.71	30	<0.0001
*Phomopsis*	7: Pathogen vs. 7: N2-1-1	−2.648	−6.090 to 0.7933	0.9339	4.01	30	0.2816
	7: Pathogen vs. 7: Nd-1-1	−4.42	−7.862 to −0.9786	0.9339	6.693	30	0.0036
	7: Pathogen vs. 7: Pb-2-2	−4.136	−7.577 to −0.6941	0.9339	6.263	30	0.008
	7: N2-1-1 vs. 7: Nd-1-1	−1.772	−5.213 to 1.670	0.9339	2.683	30	0.8366
	7: N2-1-1 vs. 7: Pb-2-2	−1.487	−4.929 to 1.954	0.9339	2.252	30	0.9474
	7: Nd-1-1 vs. 7: Pb-2-2	0.2845	−3.157 to 3.726	0.9339	0.4307	30	>0.9999
	3: Blank vs. 3: Pathogen	21.03	18.04 to 24.03	0.8123	36.62	30	<0.0001
	3: Blank vs. 3: N2-1-1	12.06	9.062 to 15.05	0.8123	20.99	30	<0.0001
	3: Blank vs. 3: Nd-1-1	13.61	10.62 to 16.61	0.8123	23.7	30	<0.0001
	3: Blank vs. 3: Pb-2-2	12.81	9.814 to 15.80	0.8123	22.3	30	<0.0001
	3: Blank vs. 5: Blank	7.373	4.379 to 10.37	0.8123	12.84	30	<0.0001
	3: Blank vs. 5: Pathogen	22.23	19.23 to 25.22	0.8123	38.69	30	<0.0001
	3: Blank vs. 5: N2-1-1	19.02	16.03 to 22.01	0.8123	33.12	30	<0.0001
	3: Blank vs. 5: Nd-1-1	17.84	14.85 to 20.84	0.8123	31.07	30	<0.0001
	3: Blank vs. 5: Pb-2-2	19.02	16.03 to 22.02	0.8123	33.12	30	<0.0001
	3: Blank vs. 7: Blank	9.785	6.791 to 12.78	0.8123	17.03	30	<0.0001
	3: Blank vs. 7: Pathogen	24.44	21.45 to 27.44	0.8123	42.55	30	<0.0001
	3: Blank vs. 7: N2-1-1	21.5	18.50 to 24.49	0.8123	37.42	30	<0.0001
	3: Blank vs. 7: Nd-1-1	19.64	16.65 to 22.63	0.8123	34.19	30	<0.0001
	3: Blank vs. 7: Pb-2-2	20.26	17.27 to 23.26	0.8123	35.28	30	<0.0001
	3: Pathogen vs. 3: N2-1-1	−8.977	−11.97 to −5.984	0.8123	15.63	30	<0.0001
	3: Pathogen vs. 3: Nd-1-1	−7.421	−10.41 to −4.428	0.8123	12.92	30	<0.0001
	3: Pathogen vs. 3: Pb-2-2	−8.225	−11.22 to −5.232	0.8123	14.32	30	<0.0001
	3: Pathogen vs. 5: Blank	−13.66	−16.65 to −10.67	0.8123	23.78	30	<0.0001
	3: Pathogen vs. 5: Pathogen	1.192	−1.801 to 4.186	0.8123	2.076	30	0.9719
	3: Pathogen vs. 5: N2-1-1	−2.012	−5.005 to 0.9817	0.8123	3.502	30	0.4838
	3: Pathogen vs. 5: Nd-1-1	−3.189	−6.182 to −0.1956	0.8123	5.552	30	0.0283
	3: Pathogen vs. 5: Pb-2-2	−2.009	−5.002 to 0.9844	0.8123	3.498	30	0.4859
	3: Pathogen vs. 7: Blank	−11.25	−14.24 to −8.255	0.8123	19.58	30	<0.0001
	3: Pathogen vs. 7: Pathogen	3.41	0.4163 to 6.403	0.8123	5.936	30	0.0145
	3: Pathogen vs. 7: N2-1-1	0.4629	−2.530 to 3.456	0.8123	0.806	30	>0.9999
	3: Pathogen vs. 7: Nd-1-1	−1.392	−4.385 to 1.601	0.8123	2.423	30	0.9125
	3: Pathogen vs. 7: Pb-2-2	−0.7687	−3.762 to 2.225	0.8123	1.338	30	0.9996
	3: N2-1-1 vs. 3: Nd-1-1	1.556	−1.437 to 4.549	0.8123	2.709	30	0.8277
	3: N2-1-1 vs. 3: Pb-2-2	0.7518	−2.242 to 3.745	0.8123	1.309	30	0.9997
	3: N2-1-1 vs. 5: Blank	−4.683	−7.677 to −1.690	0.8123	8.153	30	0.0002
	3: N2-1-1 vs. 5: Pathogen	10.17	7.176 to 13.16	0.8123	17.7	30	<0.0001
	3: N2-1-1 vs. 5: N2-1-1	6.965	3.972 to 9.959	0.8123	12.13	30	<0.0001
	3: N2-1-1 vs. 5: Nd-1-1	5.788	2.795 to 8.782	0.8123	10.08	30	<0.0001
	3: N2-1-1 vs. 5: Pb-2-2	6.968	3.975 to 9.961	0.8123	12.13	30	<0.0001
	3: N2-1-1 vs. 7: Blank	−2.271	−5.264 to 0.7222	0.8123	3.954	30	0.3009
	3: N2-1-1 vs. 7: Pathogen	12.39	9.393 to 15.38	0.8123	21.57	30	<0.0001
	3: N2-1-1 vs. 7: N2-1-1	9.44	6.447 to 12.43	0.8123	16.43	30	<0.0001
	3: N2-1-1 vs. 7: Nd-1-1	7.585	4.592 to 10.58	0.8123	13.21	30	<0.0001
	3: N2-1-1 vs. 7: Pb-2-2	8.208	5.215 to 11.20	0.8123	14.29	30	<0.0001
	3: Nd-1-1 vs. 5: N2-1-1	5.409	2.416 to 8.403	0.8123	9.418	30	<0.0001
	3: Nd-1-1 vs. 5: Nd-1-1	4.232	1.239 to 7.226	0.8123	7.368	30	0.001
	3: Nd-1-1 vs. 5: Pb-2-2	5.412	2.419 to 8.406	0.8123	9.422	30	<0.0001
	3: Nd-1-1 vs. 7: Blank	−3.827	−6.820 to −0.8337	0.8123	6.663	30	0.0038
	3: Nd-1-1 vs. 7: Pathogen	10.83	7.837 to 13.82	0.8123	18.86	30	<0.0001
	3: Nd-1-1 vs. 7: N2-1-1	7.884	4.891 to 10.88	0.8123	13.73	30	<0.0001
	3: Nd-1-1 vs. 7: Nd-1-1	6.029	3.036 to 9.023	0.8123	10.5	30	<0.0001
	3: Nd-1-1 vs. 7: Pb-2-2	6.652	3.659 to 9.646	0.8123	11.58	30	<0.0001
	3: Pb-2-2 vs. 5: Blank	−5.435	−8.428 to −2.442	0.8123	9.462	30	<0.0001
	3: Pb-2-2 vs. 5: Pathogen	9.418	6.424 to 12.41	0.8123	16.4	30	<0.0001
	3: Pb-2-2 vs. 5: N2-1-1	6.214	3.220 to 9.207	0.8123	10.82	30	<0.0001
	3: Pb-2-2 vs. 5: Nd-1-1	5.036	2.043 to 8.030	0.8123	8.768	30	<0.0001
	3: Pb-2-2 vs. 5: Pb-2-2	6.216	3.223 to 9.210	0.8123	10.82	30	<0.0001
	3: Pb-2-2 vs. 7: Blank	−3.023	−6.016 to −0.02952	0.8123	5.263	30	0.046
	3: Pb-2-2 vs. 7: Pathogen	11.64	8.642 to 14.63	0.8123	20.26	30	<0.0001
	3: Pb-2-2 vs. 7: N2-1-1	8.688	5.695 to 11.68	0.8123	15.13	30	<0.0001
	3: Pb-2-2 vs. 7: Nd-1-1	6.833	3.840 to 9.827	0.8123	11.9	30	<0.0001
	3: Pb-2-2 vs. 7: Pb-2-2	7.457	4.463 to 10.45	0.8123	12.98	30	<0.0001
	5: Blank vs. 5: Pathogen	14.85	11.86 to 17.85	0.8123	25.86	30	<0.0001
	5: Blank vs. 5: N2-1-1	11.65	8.655 to 14.64	0.8123	20.28	30	<0.0001
	5: Blank vs. 5: Nd-1-1	10.47	7.478 to 13.46	0.8123	18.23	30	<0.0001
	5: Blank vs. 5: Pb-2-2	11.65	8.658 to 14.64	0.8123	20.28	30	<0.0001
	5: Blank vs. 7: Blank	2.412	−0.5813 to 5.405	0.8123	4.199	30	0.2225
	5: Blank vs. 7: Pathogen	17.07	14.08 to 20.06	0.8123	29.72	30	<0.0001
	5: Blank vs. 7: N2-1-1	14.12	11.13 to 17.12	0.8123	24.59	30	<0.0001
	5: Blank vs. 7: Nd-1-1	12.27	9.275 to 15.26	0.8123	21.36	30	<0.0001
	5: Blank vs. 7: Pb-2-2	12.89	9.898 to 15.89	0.8123	22.44	30	<0.0001
	5: Pathogen vs. 5: N2-1-1	−3.204	−6.197 to −0.2107	0.8123	5.578	30	0.0271
	5: Pathogen vs. 5: Nd-1-1	−4.381	−7.375 to −1.388	0.8123	7.628	30	0.0006
	5: Pathogen vs. 5: Pb-2-2	−3.201	−6.195 to −0.2080	0.8123	5.573	30	0.0273
	5: Pathogen vs. 7: Blank	−12.44	−15.43 to −9.447	0.8123	21.66	30	<0.0001
	5: Pathogen vs. 7: Pathogen	2.217	−0.7760 to 5.211	0.8123	3.86	30	0.335
	5: Pathogen vs. 7: N2-1-1	−0.7294	−3.723 to 2.264	0.8123	1.27	30	0.9998
	5: Pathogen vs. 7: Nd-1-1	−2.584	−5.578 to 0.4091	0.8123	4.499	30	0.1485
	5: Pathogen vs. 7: Pb-2-2	−1.961	−4.954 to 1.032	0.8123	3.414	30	0.5236
	5: N2-1-1 vs. 5: Nd-1-1	−1.177	−4.171 to 1.816	0.8123	2.05	30	0.9746
	5: N2-1-1 vs. 5: Pb-2-2	0.002695	−2.991 to 2.996	0.8123	0.004692	30	>0.9999
	5: N2-1-1 vs. 7: Blank	−9.237	−12.23 to −6.243	0.8123	16.08	30	<0.0001
	5: N2-1-1 vs. 7: Pathogen	5.421	2.428 to 8.415	0.8123	9.439	30	<0.0001
	5: N2-1-1 vs. 7: N2-1-1	2.475	−0.5187 to 5.468	0.8123	4.308	30	0.1929
	5: N2-1-1 vs. 7: Nd-1-1	0.6197	−2.374 to 3.613	0.8123	1.079	30	>0.9999
	5: N2-1-1 vs. 7: Pb-2-2	1.243	−1.750 to 4.236	0.8123	2.164	30	0.961
	5: Nd-1-1 vs. 5: Pb-2-2	1.18	−1.813 to 4.173	0.8123	2.054	30	0.9741
	5: Nd-1-1 vs. 7: Blank	−8.059	−11.05 to −5.066	0.8123	14.03	30	<0.0001
	5: Nd-1-1 vs. 7: Pathogen	6.599	3.605 to 9.592	0.8123	11.49	30	<0.0001
	5: Nd-1-1 vs. 7: N2-1-1	3.652	0.6585 to 6.645	0.8123	6.358	30	0.0068
	5: Nd-1-1 vs. 7: Nd-1-1	1.797	−1.196 to 4.790	0.8123	3.129	30	0.6548
	5: Nd-1-1 vs. 7: Pb-2-2	2.42	−0.5731 to 5.414	0.8123	4.214	30	0.2185
	5: Pb-2-2 vs. 7: Blank	−9.239	−12.23 to −6.246	0.8123	16.09	30	<0.0001
	5: Pb-2-2 vs. 7: Pathogen	5.419	2.425 to 8.412	0.8123	9.434	30	<0.0001
	5: Pb-2-2 vs. 7: N2-1-1	2.472	−0.5214 to 5.465	0.8123	4.304	30	0.1942
	5: Pb-2-2 vs. 7: Nd-1-1	0.617	−2.376 to 3.610	0.8123	1.074	30	>0.9999
	5: Pb-2-2 vs. 7: Pb-2-2	1.24	−1.753 to 4.234	0.8123	2.159	30	0.9617
	7: Blank vs. 7: Pathogen	14.66	11.66 to 17.65	0.8123	25.52	30	<0.0001
	7: Blank vs. 7: N2-1-1	11.71	8.718 to 14.70	0.8123	20.39	30	<0.0001
	7: Blank vs. 7: Nd-1-1	9.856	6.863 to 12.85	0.8123	17.16	30	<0.0001
	7: Blank vs. 7: Pb-2-2	10.48	7.486 to 13.47	0.8123	18.24	30	<0.0001
	7: Pathogen vs. 7: N2-1-1	−2.947	−5.940 to 0.04663	0.8123	5.13	30	0.057
	7: Pathogen vs. 7: Nd-1-1	−4.802	−7.795 to −1.808	0.8123	8.36	30	0.0002
	7: Pathogen vs. 7: Pb-2-2	−4.178	−7.172 to −1.185	0.8123	7.274	30	0.0012
	7: N2-1-1 vs. 7: Nd-1-1	−1.855	−4.848 to 1.138	0.8123	3.229	30	0.6087
	7: N2-1-1 vs. 7: Pb-2-2	−1.232	−4.225 to 1.762	0.8123	2.144	30	0.9637
	7: Nd-1-1 vs. 7: Pb-2-2	0.6233	−2.370 to 3.617	0.8123	1.085	30	>0.9999

Note: APX, ascorbate peroxidase. The comparisons are denoted as “time point:treatment”, where time points “3”, “5”, and “7” indicate days post-inoculation; “Pathogen” refers to the original pathogen isolate; “N2-1-1”, “Nd-1-1”, and “Pb-2-2” are distinct microbial strains/isolates; “Blank” represents the non-inoculated control. For each pairwise comparison, the mean difference, 95% confidence interval (CI) of the difference, standard error of the difference (SE), studentized range statistic (q), degrees of freedom (df), and adjusted *p*-value (Tukey’s method) are presented. Due to a balanced design and homogeneity of variance, the SE of the difference (0.8123) and the degrees of freedom (30) are constant across all comparisons. The critical significance level was set at α = 0.05, and adjusted *p*-values were computed to control the family-wise error rate.

**Table A9:** Tukey’s multiple comparisons test for PAL enzyme activity.

Pathogen	Comparison	Mean Difference	95.00% CI	SE of Difference	q	*df*	Adjusted *p*
*Alternaria*	3: Blank vs. 3: Pathogen	3.651	−4.311 to 11.61	2.161	2.39	30	0.9203
	3: Blank vs. 3: N2-1-1	−8.687	−16.65 to −0.7245	2.161	5.686	30	0.0225
	3: Blank vs. 3: Nd-1-1	−11.33	−19.30 to −3.371	2.161	7.417	30	0.0009
	3: Blank vs. 3: Pb-2-2	−10.64	−18.61 to −2.680	2.161	6.965	30	0.0022
	3: Blank vs. 5: Blank	−10.95	−18.92 to −2.992	2.161	7.169	30	0.0015
	3: Blank vs. 5: Pathogen	−1.302	−9.265 to 6.661	2.161	0.8522	30	>0.9999
	3: Blank vs. 5: N2-1-1	−15.89	−23.85 to −7.927	2.161	10.4	30	<0.0001
	3: Blank vs. 5: Nd-1-1	−13.81	−21.77 to −5.847	2.161	9.038	30	<0.0001
	3: Blank vs. 5: Pb-2-2	−13.85	−21.81 to −5.883	2.161	9.061	30	<0.0001
	3: Blank vs. 7: Blank	−21.52	−29.49 to −13.56	2.161	14.09	30	<0.0001
	3: Blank vs. 7: Pathogen	0.656	−7.307 to 8.619	2.161	0.4294	30	>0.9999
	3: Blank vs. 7: N2-1-1	−8.034	−16.00 to −0.07076	2.161	5.258	30	0.0463
	3: Blank vs. 7: Nd-1-1	−8.404	−16.37 to −0.4412	2.161	5.5	30	0.0309
	3: Blank vs. 7: Pb-2-2	−8.425	−16.39 to −0.4627	2.161	5.514	30	0.0302
	3: Pathogen vs. 3: N2-1-1	−12.34	−20.30 to −4.376	2.161	8.075	30	0.0003
	3: Pathogen vs. 3: Nd-1-1	−14.98	−22.95 to −7.022	2.161	9.807	30	<0.0001
	3: Pathogen vs. 3: Pb-2-2	−14.29	−22.26 to −6.331	2.161	9.355	30	<0.0001
	3: Pathogen vs. 5: Blank	−14.61	−22.57 to −6.643	2.161	9.559	30	<0.0001
	3: Pathogen vs. 5: Pathogen	−4.954	−12.92 to 3.009	2.161	3.242	30	0.6029
	3: Pathogen vs. 5: N2-1-1	−19.54	−27.50 to −11.58	2.161	12.79	30	<0.0001
	3: Pathogen vs. 5: Nd-1-1	−17.46	−25.42 to −9.498	2.161	11.43	30	<0.0001
	3: Pathogen vs. 5: Pb-2-2	−17.5	−25.46 to −9.534	2.161	11.45	30	<0.0001
	3: Pathogen vs. 7: Blank	−25.17	−33.14 to −17.21	2.161	16.48	30	<0.0001
	3: Pathogen vs. 7: Pathogen	−2.995	−10.96 to 4.967	2.161	1.96	30	0.9825
	3: Pathogen vs. 7: N2-1-1	−11.69	−19.65 to −3.722	2.161	7.647	30	0.0006
	3: Pathogen vs. 7: Nd-1-1	−12.06	−20.02 to −4.093	2.161	7.89	30	0.0004
	3: Pathogen vs. 7: Pb-2-2	−12.08	−20.04 to −4.114	2.161	7.904	30	0.0004
	3: N2-1-1 vs. 3: Nd-1-1	−2.646	−10.61 to 5.317	2.161	1.732	30	0.9942
	3: N2-1-1 vs. 3: Pb-2-2	−1.955	−9.918 to 6.008	2.161	1.28	30	0.9998
	3: N2-1-1 vs. 5: Blank	−2.267	−10.23 to 5.696	2.161	1.484	30	0.9988
	3: N2-1-1 vs. 5: Pathogen	7.385	−0.5776 to 15.35	2.161	4.833	30	0.0909
	3: N2-1-1 vs. 5: N2-1-1	−7.203	−15.17 to 0.7600	2.161	4.714	30	0.1088
	3: N2-1-1 vs. 5: Nd-1-1	−5.123	−13.09 to 2.840	2.161	3.353	30	0.5519
	3: N2-1-1 vs. 5: Pb-2-2	−5.158	−13.12 to 2.805	2.161	3.376	30	0.5411
	3: N2-1-1 vs. 7: Blank	−12.84	−20.80 to −4.873	2.161	8.4	30	0.0001
	3: N2-1-1 vs. 7: Pathogen	9.343	1.381 to 17.31	2.161	6.115	30	0.0105
	3: N2-1-1 vs. 7: N2-1-1	0.6537	−7.309 to 8.617	2.161	0.4278	30	>0.9999
	3: N2-1-1 vs. 7: Nd-1-1	0.2833	−7.680 to 8.246	2.161	0.1854	30	>0.9999
	3: N2-1-1 vs. 7: Pb-2-2	0.2618	−7.701 to 8.225	2.161	0.1714	30	>0.9999
	3: Nd-1-1 vs. 3: Pb-2-2	0.6911	−7.272 to 8.654	2.161	0.4523	30	>0.9999
	3: Nd-1-1 vs. 5: Blank	0.3791	−7.584 to 8.342	2.161	0.2481	30	>0.9999
	3: Nd-1-1 vs. 5: Pathogen	10.03	2.069 to 17.99	2.161	6.565	30	0.0046
	3: Nd-1-1 vs. 5: N2-1-1	−4.557	−12.52 to 3.406	2.161	2.982	30	0.7198
	3: Nd-1-1 vs. 5: Nd-1-1	−2.476	−10.44 to 5.487	2.161	1.621	30	0.997
	3: Nd-1-1 vs. 5: Pb-2-2	−2.512	−10.47 to 5.451	2.161	1.644	30	0.9965
	3: Nd-1-1 vs. 7: Blank	−10.19	−18.15 to −2.226	2.161	6.668	30	0.0038
	3: Nd-1-1 vs. 7: Pathogen	11.99	4.027 to 19.95	2.161	7.847	30	0.0004
	3: Nd-1-1 vs. 7: N2-1-1	3.3	−4.663 to 11.26	2.161	2.16	30	0.9616
	3: Nd-1-1 vs. 7: Nd-1-1	2.93	−5.033 to 10.89	2.161	1.917	30	0.9855
	3: Nd-1-1 vs. 7: Pb-2-2	2.908	−5.055 to 10.87	2.161	1.903	30	0.9864
	3: Pb-2-2 vs. 5: Blank	−0.312	−8.275 to 7.651	2.161	0.2042	30	>0.9999
	3: Pb-2-2 vs. 5: Pathogen	9.34	1.378 to 17.30	2.161	6.113	30	0.0105
	3: Pb-2-2 vs. 5: N2-1-1	−5.248	−13.21 to 2.715	2.161	3.434	30	0.5144
	3: Pb-2-2 vs. 5: Nd-1-1	−3.167	−11.13 to 4.795	2.161	2.073	30	0.9722
	3: Pb-2-2 vs. 5: Pb-2-2	−3.203	−11.17 to 4.760	2.161	2.096	30	0.9696
	3: Pb-2-2 vs. 7: Blank	−10.88	−18.84 to −2.917	2.161	7.121	30	0.0016
	3: Pb-2-2 vs. 7: Pathogen	11.3	3.336 to 19.26	2.161	7.394	30	0.001
	3: Pb-2-2 vs. 7: N2-1-1	2.609	−5.354 to 10.57	2.161	1.707	30	0.995
	3: Pb-2-2 vs. 7: Nd-1-1	2.238	−5.724 to 10.20	2.161	1.465	30	0.9989
	3: Pb-2-2 vs. 7: Pb-2-2	2.217	−5.746 to 10.18	2.161	1.451	30	0.999
	5: Blank vs. 5: Pathogen	9.652	1.690 to 17.62	2.161	6.317	30	0.0073
	5: Blank vs. 5: N2-1-1	−4.936	−12.90 to 3.027	2.161	3.23	30	0.6083
	5: Blank vs. 5: Nd-1-1	−2.855	−10.82 to 5.107	2.161	1.869	30	0.9884
	5: Blank vs. 5: Pb-2-2	−2.891	−10.85 to 5.072	2.161	1.892	30	0.9871
	5: Blank vs. 7: Blank	−10.57	−18.53 to −2.605	2.161	6.917	30	0.0024
	5: Blank vs. 7: Pathogen	11.61	3.648 to 19.57	2.161	7.599	30	0.0007
	5: Blank vs. 7: N2-1-1	2.921	−5.042 to 10.88	2.161	1.912	30	0.9859
	5: Blank vs. 7: Nd-1-1	2.55	−5.412 to 10.51	2.161	1.669	30	0.996
	5: Blank vs. 7: Pb-2-2	2.529	−5.434 to 10.49	2.161	1.655	30	0.9963
	5: Pathogen vs. 5: N2-1-1	−14.59	−22.55 to −6.625	2.161	9.547	30	<0.0001
	5: Pathogen vs. 5: Nd-1-1	−12.51	−20.47 to −4.545	2.161	8.186	30	0.0002
	5: Pathogen vs. 5: Pb-2-2	−12.54	−20.51 to −4.581	2.161	8.209	30	0.0002
	5: Pathogen vs. 7: Blank	−20.22	−28.18 to −12.26	2.161	13.23	30	<0.0001
	5: Pathogen vs. 7: Pathogen	1.958	−6.005 to 9.921	2.161	1.282	30	0.9997
	5: Pathogen vs. 7: N2-1-1	−6.732	−14.69 to 1.231	2.161	4.406	30	0.1692
	5: Pathogen vs. 7: Nd-1-1	−7.102	−15.06 to 0.8609	2.161	4.648	30	0.1199
	5: Pathogen vs. 7: Pb-2-2	−7.123	−15.09 to 0.8394	2.161	4.662	30	0.1175
	5: N2-1-1 vs. 5: Nd-1-1	2.08	−5.883 to 10.04	2.161	1.361	30	0.9995
	5: N2-1-1 vs. 5: Pb-2-2	2.045	−5.918 to 10.01	2.161	1.338	30	0.9996
	5: N2-1-1 vs. 7: Blank	−5.633	−13.60 to 2.330	2.161	3.686	30	0.4041
	5: N2-1-1 vs. 7: Pathogen	16.55	8.583 to 24.51	2.161	10.83	30	<0.0001
	5: N2-1-1 vs. 7: N2-1-1	7.857	−0.1063 to 15.82	2.161	5.142	30	0.056
	5: N2-1-1 vs. 7: Nd-1-1	7.486	−0.4767 to 15.45	2.161	4.899	30	0.0821
	5: N2-1-1 vs. 7: Pb-2-2	7.465	−0.4982 to 15.43	2.161	4.885	30	0.0839
	5: Nd-1-1 vs. 5: Pb-2-2	−0.03577	−7.999 to 7.927	2.161	0.02341	30	>0.9999
	5: Nd-1-1 vs. 7: Blank	−7.713	−15.68 to 0.2499	2.161	5.048	30	0.0651
	5: Nd-1-1 vs. 7: Pathogen	14.47	6.503 to 22.43	2.161	9.467	30	<0.0001
	5: Nd-1-1 vs. 7: N2-1-1	5.776	−2.187 to 13.74	2.161	3.78	30	0.3659
	5: Nd-1-1 vs. 7: Nd-1-1	5.406	−2.557 to 13.37	2.161	3.538	30	0.4679
	5: Nd-1-1 vs. 7: Pb-2-2	5.384	−2.578 to 13.35	2.161	3.524	30	0.4742
	5: Pb-2-2 vs. 7: Blank	−7.677	−15.64 to 0.2857	2.161	5.024	30	0.0675
	5: Pb-2-2 vs. 7: Pathogen	14.5	6.539 to 22.46	2.161	9.491	30	<0.0001
	5: Pb-2-2 vs. 7: N2-1-1	5.812	−2.151 to 13.77	2.161	3.804	30	0.3567
	5: Pb-2-2 vs. 7: Nd-1-1	5.442	−2.521 to 13.40	2.161	3.561	30	0.4576
	5: Pb-2-2 vs. 7: Pb-2-2	5.42	−2.543 to 13.38	2.161	3.547	30	0.4638
	7: Blank vs. 7: Pathogen	22.18	14.22 to 30.14	2.161	14.52	30	<0.0001
	7: Blank vs. 7: N2-1-1	13.49	5.526 to 21.45	2.161	8.828	30	<0.0001
	7: Blank vs. 7: Nd-1-1	13.12	5.156 to 21.08	2.161	8.586	30	<0.0001
	7: Blank vs. 7: Pb-2-2	13.1	5.134 to 21.06	2.161	8.572	30	0.0001
	7: Pathogen vs. 7: N2-1-1	−8.69	−16.65 to −0.7268	2.161	5.687	30	0.0224
	7: Pathogen vs. 7: Nd-1-1	−9.06	−17.02 to −1.097	2.161	5.929	30	0.0146
	7: Pathogen vs. 7: Pb-2-2	−9.082	−17.04 to −1.119	2.161	5.944	30	0.0143
	7: N2-1-1 vs. 7: Nd-1-1	−0.3704	−8.333 to 7.592	2.161	0.2424	30	>0.9999
	7: N2-1-1 vs. 7: Pb-2-2	−0.3919	−8.355 to 7.571	2.161	0.2565	30	>0.9999
*Fusarium*	7: Nd-1-1 vs. 7: Pb-2-2	−0.02147	−7.984 to 7.941	2.161	0.01405	30	>0.9999
	3: Blank vs. 3: Pathogen	1.74	−4.164 to 7.643	1.602	1.536	30	0.9982
	3: Blank vs. 3: N2-1-1	−10.84	−16.74 to −4.932	1.602	9.565	30	<0.0001
	3: Blank vs. 3: Nd-1-1	−10.37	−16.28 to −4.470	1.602	9.157	30	<0.0001
	3: Blank vs. 3: Pb-2-2	−10.46	−16.36 to −4.556	1.602	9.233	30	<0.0001
	3: Blank vs. 5: Blank	−10.95	−16.86 to −5.051	1.602	9.67	30	<0.0001
	3: Blank vs. 5: Pathogen	−5.775	−11.68 to 0.1282	1.602	5.098	30	0.06
	3: Blank vs. 5: N2-1-1	−25.41	−31.32 to −19.51	1.602	22.43	30	<0.0001
	3: Blank vs. 5: Nd-1-1	−23.92	−29.82 to −18.02	1.602	21.11	30	<0.0001
	3: Blank vs. 5: Pb-2-2	−26.4	−32.30 to −20.50	1.602	23.31	30	<0.0001
	3: Blank vs. 7: Blank	−21.52	−27.43 to −15.62	1.602	19	30	<0.0001
	3: Blank vs. 7: Pathogen	−2.045	−7.949 to 3.859	1.602	1.805	30	0.9915
	3: Blank vs. 7: N2-1-1	−16.34	−22.25 to −10.44	1.602	14.43	30	<0.0001
	3: Blank vs. 7: Nd-1-1	−11.33	−17.24 to −5.431	1.602	10.01	30	<0.0001
	3: Blank vs. 7: Pb-2-2	−14.32	−20.22 to −8.414	1.602	12.64	30	<0.0001
	3: Pathogen vs. 3: N2-1-1	−12.58	−18.48 to −6.672	1.602	11.1	30	<0.0001
	3: Pathogen vs. 3: Nd-1-1	−12.11	−18.02 to −6.209	1.602	10.69	30	<0.0001
	3: Pathogen vs. 3: Pb-2-2	−12.2	−18.10 to −6.295	1.602	10.77	30	<0.0001
	3: Pathogen vs. 5: Blank	−12.69	−18.60 to −6.791	1.602	11.21	30	<0.0001
	3: Pathogen vs. 5: Pathogen	−7.515	−13.42 to −1.612	1.602	6.634	30	0.0041
	3: Pathogen vs. 5: N2-1-1	−27.15	−33.06 to −21.25	1.602	23.97	30	<0.0001
	3: Pathogen vs. 5: Nd-1-1	−25.66	−31.56 to −19.76	1.602	22.65	30	<0.0001
	3: Pathogen vs. 5: Pb-2-2	−28.14	−34.04 to −22.24	1.602	24.84	30	<0.0001
	3: Pathogen vs. 7: Blank	−23.26	−29.17 to −17.36	1.602	20.53	30	<0.0001
	3: Pathogen vs. 7: Pathogen	−3.785	−9.688 to 2.119	1.602	3.341	30	0.5572
	3: Pathogen vs. 7: N2-1-1	−18.08	−23.99 to −12.18	1.602	15.96	30	<0.0001
	3: Pathogen vs. 7: Nd-1-1	−13.07	−18.98 to −7.171	1.602	11.54	30	<0.0001
	3: Pathogen vs. 7: Pb-2-2	−16.06	−21.96 to −10.15	1.602	14.17	30	<0.0001
	3: N2-1-1 vs. 3: Nd-1-1	0.4626	−5.441 to 6.366	1.602	0.4084	30	>0.9999
	3: N2-1-1 vs. 3: Pb-2-2	0.3765	−5.527 to 6.280	1.602	0.3324	30	>0.9999
	3: N2-1-1 vs. 5: Blank	−0.1187	−6.022 to 5.785	1.602	0.1048	30	>0.9999
	3: N2-1-1 vs. 5: Pathogen	5.06	−0.8432 to 10.96	1.602	4.467	30	0.1553
	3: N2-1-1 vs. 5: N2-1-1	−14.58	−20.48 to −8.675	1.602	12.87	30	<0.0001
	3: N2-1-1 vs. 5: Nd-1-1	−13.08	−18.99 to −7.180	1.602	11.55	30	<0.0001
	3: N2-1-1 vs. 5: Pb-2-2	−15.57	−21.47 to −9.662	1.602	13.74	30	<0.0001
	3: N2-1-1 vs. 7: Blank	−10.69	−16.59 to −4.783	1.602	9.434	30	<0.0001
	3: N2-1-1 vs. 7: Pathogen	8.791	2.887 to 14.69	1.602	7.76	30	0.0005
	3: N2-1-1 vs. 7: N2-1-1	−5.508	−11.41 to 0.3956	1.602	4.862	30	0.087
	3: N2-1-1 vs. 7: Nd-1-1	−0.4987	−6.402 to 5.405	1.602	0.4403	30	>0.9999
	3: N2-1-1 vs. 7: Pb-2-2	−3.482	−9.385 to 2.422	1.602	3.073	30	0.6796
	3: Nd-1-1 vs. 3: Pb-2-2	−0.0861	−5.990 to 5.818	1.602	0.076	30	>0.9999
	3: Nd-1-1 vs. 5: Blank	−0.5813	−6.485 to 5.322	1.602	0.5131	30	>0.9999
	3: Nd-1-1 vs. 5: Pathogen	4.598	−1.306 to 10.50	1.602	4.059	30	0.2655
	3: Nd-1-1 vs. 5: N2-1-1	−15.04	−20.94 to −9.137	1.602	13.28	30	<0.0001
	3: Nd-1-1 vs. 5: Nd-1-1	−13.55	−19.45 to −7.642	1.602	11.96	30	<0.0001
	3: Nd-1-1 vs. 5: Pb-2-2	−16.03	−21.93 to −10.12	1.602	14.15	30	<0.0001
	3: Nd-1-1 vs. 7: Blank	−11.15	−17.05 to −5.246	1.602	9.842	30	<0.0001
	3: Nd-1-1 vs. 7: Pathogen	8.328	2.425 to 14.23	1.602	7.352	30	0.001
	3: Nd-1-1 vs. 7: N2-1-1	−5.971	−11.87 to −0.06695	1.602	5.27	30	0.0454
	3: Nd-1-1 vs. 7: Nd-1-1	−0.9613	−6.865 to 4.942	1.602	0.8486	30	>0.9999
	3: Nd-1-1 vs. 7: Pb-2-2	−3.944	−9.848 to 1.959	1.602	3.482	30	0.4929
	3: Pb-2-2 vs. 5: Blank	−0.4952	−6.399 to 5.408	1.602	0.4371	30	>0.9999
	3: Pb-2-2 vs. 5: Pathogen	4.684	−1.220 to 10.59	1.602	4.135	30	0.2416
	3: Pb-2-2 vs. 5: N2-1-1	−14.95	−20.86 to −9.051	1.602	13.2	30	<0.0001
	3: Pb-2-2 vs. 5: Nd-1-1	−13.46	−19.36 to −7.556	1.602	11.88	30	<0.0001
	3: Pb-2-2 vs. 5: Pb-2-2	−15.94	−21.85 to −10.04	1.602	14.07	30	<0.0001
	3: Pb-2-2 vs. 7: Blank	−11.06	−16.97 to −5.160	1.602	9.766	30	<0.0001
	3: Pb-2-2 vs. 7: Pathogen	8.414	2.511 to 14.32	1.602	7.428	30	0.0009
	3: Pb-2-2 vs. 7: N2-1-1	−5.884	−11.79 to 0.01915	1.602	5.194	30	0.0514
	3: Pb-2-2 vs. 7: Nd-1-1	−0.8752	−6.779 to 5.028	1.602	0.7726	30	>0.9999
	3: Pb-2-2 vs. 7: Pb-2-2	−3.858	−9.762 to 2.045	1.602	3.406	30	0.5274
	5: Blank vs. 5: Pathogen	5.179	−0.7245 to 11.08	1.602	4.572	30	0.1339
	5: Blank vs. 5: N2-1-1	−14.46	−20.36 to −8.556	1.602	12.76	30	<0.0001
	5: Blank vs. 5: Nd-1-1	−12.96	−18.87 to −7.061	1.602	11.44	30	<0.0001
	5: Blank vs. 5: Pb-2-2	−15.45	−21.35 to −9.543	1.602	13.64	30	<0.0001
	5: Blank vs. 7: Blank	−10.57	−16.47 to −4.665	1.602	9.329	30	<0.0001
	5: Blank vs. 7: Pathogen	8.91	3.006 to 14.81	1.602	7.865	30	0.0004
	5: Blank vs. 7: N2-1-1	−5.389	−11.29 to 0.5144	1.602	4.757	30	0.102
	5: Blank vs. 7: Nd-1-1	−0.38	−6.284 to 5.524	1.602	0.3355	30	>0.9999
	5: Blank vs. 7: Pb-2-2	−3.363	−9.267 to 2.541	1.602	2.969	30	0.7255
	5: Pathogen vs. 5: N2-1-1	−19.64	−25.54 to −13.74	1.602	17.34	30	<0.0001
	5: Pathogen vs. 5: Nd-1-1	−18.14	−24.05 to −12.24	1.602	16.02	30	<0.0001
	5: Pathogen vs. 5: Pb-2-2	−20.63	−26.53 to −14.72	1.602	18.21	30	<0.0001
	5: Pathogen vs. 7: Blank	−15.75	−21.65 to −9.844	1.602	13.9	30	<0.0001
	5: Pathogen vs. 7: Pathogen	3.73	−2.173 to 9.634	1.602	3.293	30	0.5793
	5: Pathogen vs. 7: N2-1-1	−10.57	−16.47 to −4.665	1.602	9.329	30	<0.0001
	5: Pathogen vs. 7: Nd-1-1	−5.559	−11.46 to 0.3445	1.602	4.907	30	0.0811
	5: Pathogen vs. 7: Pb-2-2	−8.542	−14.45 to −2.639	1.602	7.541	30	0.0007
	5: N2-1-1 vs. 5: Nd-1-1	1.495	−4.408 to 7.399	1.602	1.32	30	0.9997
	5: N2-1-1 vs. 5: Pb-2-2	−0.9871	−6.891 to 4.917	1.602	0.8714	30	>0.9999
	5: N2-1-1 vs. 7: Blank	3.892	−2.012 to 9.795	1.602	3.435	30	0.514
	5: N2-1-1 vs. 7: Pathogen	23.37	17.47 to 29.27	1.602	20.63	30	<0.0001
	5: N2-1-1 vs. 7: N2-1-1	9.07	3.167 to 14.97	1.602	8.007	30	0.0003
	5: N2-1-1 vs. 7: Nd-1-1	14.08	8.176 to 19.98	1.602	12.43	30	<0.0001
	5: N2-1-1 vs. 7: Pb-2-2	11.1	5.193 to 17.00	1.602	9.796	30	<0.0001
	5: Nd-1-1 vs. 5: Pb-2-2	−2.482	−8.386 to 3.421	1.602	2.191	30	0.9571
	5: Nd-1-1 vs. 7: Blank	2.396	−3.507 to 8.300	1.602	2.115	30	0.9673
	5: Nd-1-1 vs. 7: Pathogen	21.87	15.97 to 27.78	1.602	19.31	30	<0.0001
	5: Nd-1-1 vs. 7: N2-1-1	7.575	1.672 to 13.48	1.602	6.687	30	0.0037
	5: Nd-1-1 vs. 7: Nd-1-1	12.58	6.681 to 18.49	1.602	11.11	30	<0.0001
	5: Nd-1-1 vs. 7: Pb-2-2	9.601	3.698 to 15.51	1.602	8.476	30	0.0001
	5: Pb-2-2 vs. 7: Blank	4.879	−1.025 to 10.78	1.602	4.307	30	0.1934
	5: Pb-2-2 vs. 7: Pathogen	24.36	18.45 to 30.26	1.602	21.5	30	<0.0001
	5: Pb-2-2 vs. 7: N2-1-1	10.06	4.154 to 15.96	1.602	8.878	30	<0.0001
	5: Pb-2-2 vs. 7: Nd-1-1	15.07	9.163 to 20.97	1.602	13.3	30	<0.0001
	5: Pb-2-2 vs. 7: Pb-2-2	12.08	6.180 to 17.99	1.602	10.67	30	<0.0001
	7: Blank vs. 7: Pathogen	19.48	13.57 to 25.38	1.602	17.19	30	<0.0001
	7: Blank vs. 7: N2-1-1	5.179	−0.7247 to 11.08	1.602	4.572	30	0.1339
	7: Blank vs. 7: Nd-1-1	10.19	4.285 to 16.09	1.602	8.994	30	<0.0001
	7: Blank vs. 7: Pb-2-2	7.205	1.302 to 13.11	1.602	6.36	30	0.0067
	7: Pathogen vs. 7: N2-1-1	−14.3	−20.20 to −8.395	1.602	12.62	30	<0.0001
	7: Pathogen vs. 7: Nd-1-1	−9.29	−15.19 to −3.386	1.602	8.2	30	0.0002
	7: Pathogen vs. 7: Pb-2-2	−12.27	−18.18 to −6.369	1.602	10.83	30	<0.0001
	7: N2-1-1 vs. 7: Nd-1-1	5.009	−0.8944 to 10.91	1.602	4.422	30	0.1654
	7: N2-1-1 vs. 7: Pb-2-2	2.026	−3.877 to 7.930	1.602	1.789	30	0.9922
	7: Nd-1-1 vs. 7: Pb-2-2	−2.983	−8.887 to 2.921	1.602	2.633	30	0.8533
	3: Blank vs. 3: Pathogen	8.803	2.746 to 14.86	1.644	7.574	30	0.0007
	3: Blank vs. 3: N2-1-1	0.5349	−5.522 to 6.592	1.644	0.4603	30	>0.9999
	3: Blank vs. 3: Nd-1-1	0.9402	−5.117 to 6.997	1.644	0.809	30	>0.9999
	3: Blank vs. 3: Pb-2-2	−0.1668	−6.224 to 5.890	1.644	0.1435	30	>0.9999
	3: Blank vs. 5: Blank	−10.95	−17.01 to −4.898	1.644	9.425	30	<0.0001
	3: Blank vs. 5: Pathogen	1.697	−4.360 to 7.753	1.644	1.46	30	0.999
	3: Blank vs. 5: N2-1-1	−13.25	−19.31 to −7.191	1.644	11.4	30	<0.0001
	3: Blank vs. 5: Nd-1-1	−14.62	−20.68 to −8.568	1.644	12.58	30	<0.0001
	3: Blank vs. 5: Pb-2-2	−16.05	−22.11 to −9.995	1.644	13.81	30	<0.0001
	3: Blank vs. 7: Blank	−21.52	−27.58 to −15.47	1.644	18.52	30	<0.0001
	3: Blank vs. 7: Pathogen	5.954	−0.1031 to 12.01	1.644	5.123	30	0.0577
	3: Blank vs. 7: N2-1-1	−4.835	−10.89 to 1.221	1.644	4.16	30	0.2339
	3: Blank vs. 7: Nd-1-1	−6.581	−12.64 to −0.5240	1.644	5.662	30	0.0234
	3: Blank vs. 7: Pb-2-2	−8.099	−14.16 to −2.042	1.644	6.968	30	0.0022
	3: Pathogen vs. 3: N2-1-1	−8.268	−14.32 to −2.211	1.644	7.114	30	0.0016
	3: Pathogen vs. 3: Nd-1-1	−7.862	−13.92 to −1.806	1.644	6.765	30	0.0032
	3: Pathogen vs. 3: Pb-2-2	−8.969	−15.03 to −2.913	1.644	7.717	30	0.0005
	3: Pathogen vs. 5: Blank	−19.76	−25.81 to −13.70	1.644	17	30	<0.0001
	3: Pathogen vs. 5: Pathogen	−7.106	−13.16 to −1.049	1.644	6.114	30	0.0105
	3: Pathogen vs. 5: N2-1-1	−22.05	−28.11 to −15.99	1.644	18.97	30	<0.0001
	3: Pathogen vs. 5: Nd-1-1	−23.43	−29.48 to −17.37	1.644	20.16	30	<0.0001
	3: Pathogen vs. 5: Pb-2-2	−24.85	−30.91 to −18.80	1.644	21.39	30	<0.0001
	3: Pathogen vs. 7: Blank	−30.33	−36.38 to −24.27	1.644	26.09	30	<0.0001
*Botryosphaeriaceae*	3: Pathogen vs. 7: Pathogen	−2.849	−8.906 to 3.208	1.644	2.451	30	0.9057
	3: Pathogen vs. 7: N2-1-1	−13.64	−19.69 to −7.581	1.644	11.73	30	<0.0001
	3: Pathogen vs. 7: Nd-1-1	−15.38	−21.44 to −9.327	1.644	13.24	30	<0.0001
	3: Pathogen vs. 7: Pb-2-2	−16.9	−22.96 to −10.84	1.644	14.54	30	<0.0001
	3: N2-1-1 vs. 3: Nd-1-1	0.4053	−5.652 to 6.462	1.644	0.3487	30	>0.9999
	3: N2-1-1 vs. 3: Pb-2-2	−0.7017	−6.759 to 5.355	1.644	0.6038	30	>0.9999
	3: N2-1-1 vs. 5: Blank	−11.49	−17.55 to −5.433	1.644	9.886	30	<0.0001
	3: N2-1-1 vs. 5: Pathogen	1.162	−4.895 to 7.218	1.644	0.9995	30	>0.9999
	3: N2-1-1 vs. 5: N2-1-1	−13.78	−19.84 to −7.726	1.644	11.86	30	<0.0001
	3: N2-1-1 vs. 5: Nd-1-1	−15.16	−21.22 to −9.103	1.644	13.04	30	<0.0001
	3: N2-1-1 vs. 5: Pb-2-2	−16.59	−22.64 to −10.53	1.644	14.27	30	<0.0001
	3: N2-1-1 vs. 7: Blank	−22.06	−28.11 to −16.00	1.644	18.98	30	<0.0001
	3: N2-1-1 vs. 7: Pathogen	5.419	−0.6380 to 11.48	1.644	4.662	30	0.1174
	3: N2-1-1 vs. 7: N2-1-1	−5.37	−11.43 to 0.6865	1.644	4.621	30	0.1248
	3: N2-1-1 vs. 7: Nd-1-1	−7.116	−13.17 to −1.059	1.644	6.123	30	0.0104
	3: N2-1-1 vs. 7: Pb-2-2	−8.634	−14.69 to −2.577	1.644	7.429	30	0.0009
	3: Nd-1-1 vs. 3: Pb-2-2	−1.107	−7.164 to 4.950	1.644	0.9525	30	>0.9999
	3: Nd-1-1 vs. 5: Blank	−11.89	−17.95 to −5.838	1.644	10.23	30	<0.0001
	3: Nd-1-1 vs. 5: Pathogen	0.7563	−5.301 to 6.813	1.644	0.6507	30	>0.9999
	3: Nd-1-1 vs. 5: N2-1-1	−14.19	−20.25 to −8.132	1.644	12.21	30	<0.0001
	3: Nd-1-1 vs. 5: Nd-1-1	−15.57	−21.62 to −9.508	1.644	13.39	30	<0.0001
	3: Nd-1-1 vs. 5: Pb-2-2	−16.99	−23.05 to −10.94	1.644	14.62	30	<0.0001
	3: Nd-1-1 vs. 7: Blank	−22.46	−28.52 to −16.41	1.644	19.33	30	<0.0001
	3: Nd-1-1 vs. 7: Pathogen	5.013	−1.043 to 11.07	1.644	4.314	30	0.1916
	3: Nd-1-1 vs. 7: N2-1-1	−5.776	−11.83 to 0.2812	1.644	4.969	30	0.0736
	3: Nd-1-1 vs. 7: Nd-1-1	−7.521	−13.58 to −1.464	1.644	6.471	30	0.0055
	3: Nd-1-1 vs. 7: Pb-2-2	−9.039	−15.10 to −2.982	1.644	7.777	30	0.0005
	3: Pb-2-2 vs. 5: Blank	−10.79	−16.84 to −4.731	1.644	9.282	30	<0.0001
	3: Pb-2-2 vs. 5: Pathogen	1.863	−4.194 to 7.920	1.644	1.603	30	0.9973
	3: Pb-2-2 vs. 5: N2-1-1	−13.08	−19.14 to −7.025	1.644	11.26	30	<0.0001
	3: Pb-2-2 vs. 5: Nd-1-1	−14.46	−20.51 to −8.401	1.644	12.44	30	<0.0001
	3: Pb-2-2 vs. 5: Pb-2-2	−15.89	−21.94 to −9.829	1.644	13.67	30	<0.0001
	3: Pb-2-2 vs. 7: Blank	−21.36	−27.41 to −15.30	1.644	18.37	30	<0.0001
	3: Pb-2-2 vs. 7: Pathogen	6.121	0.06367 to 12.18	1.644	5.266	30	0.0457
	3: Pb-2-2 vs. 7: N2-1-1	−4.669	−10.73 to 1.388	1.644	4.017	30	0.2792
	3: Pb-2-2 vs. 7: Nd-1-1	−6.414	−12.47 to −0.3573	1.644	5.519	30	0.0299
	3: Pb-2-2 vs. 7: Pb-2-2	−7.932	−13.99 to −1.875	1.644	6.825	30	0.0028
	5: Blank vs. 5: Pathogen	12.65	6.594 to 18.71	1.644	10.89	30	<0.0001
	5: Blank vs. 5: N2-1-1	−2.294	−8.351 to 3.763	1.644	1.974	30	0.9814
	5: Blank vs. 5: Nd-1-1	−3.67	−9.727 to 2.386	1.644	3.158	30	0.6414
	5: Blank vs. 5: Pb-2-2	−5.098	−11.15 to 0.9592	1.644	4.386	30	0.1737
	5: Blank vs. 7: Blank	−10.57	−16.63 to −4.511	1.644	9.093	30	<0.0001
	5: Blank vs. 7: Pathogen	16.91	10.85 to 22.97	1.644	14.55	30	<0.0001
	5: Blank vs. 7: N2-1-1	6.119	0.06221 to 12.18	1.644	5.265	30	0.0458
	5: Blank vs. 7: Nd-1-1	4.374	−1.683 to 10.43	1.644	3.763	30	0.3728
	5: Blank vs. 7: Pb-2-2	2.856	−3.201 to 8.912	1.644	2.457	30	0.9042
	5: Pathogen vs. 5: N2-1-1	−14.94	−21.00 to −8.888	1.644	12.86	30	<0.0001
	5: Pathogen vs. 5: Nd-1-1	−16.32	−22.38 to −10.26	1.644	14.04	30	<0.0001
	5: Pathogen vs. 5: Pb-2-2	−17.75	−23.81 to −11.69	1.644	15.27	30	<0.0001
	5: Pathogen vs. 7: Blank	−23.22	−29.28 to −17.16	1.644	19.98	30	<0.0001
	5: Pathogen vs. 7: Pathogen	4.257	−1.800 to 10.31	1.644	3.663	30	0.4139
	5: Pathogen vs. 7: N2-1-1	−6.532	−12.59 to −0.4751	1.644	5.62	30	0.0252
	5: Pathogen vs. 7: Nd-1-1	−8.277	−14.33 to −2.221	1.644	7.122	30	0.0016
	5: Pathogen vs. 7: Pb-2-2	−9.795	−15.85 to −3.739	1.644	8.428	30	0.0001
	5: N2-1-1 vs. 5: Nd-1-1	−1.377	−7.434 to 4.680	1.644	1.185	30	0.9999
	5: N2-1-1 vs. 5: Pb-2-2	−2.804	−8.861 to 3.253	1.644	2.412	30	0.9151
	5: N2-1-1 vs. 7: Blank	−8.274	−14.33 to −2.218	1.644	7.119	30	0.0016
	5: N2-1-1 vs. 7: Pathogen	19.2	13.15 to 25.26	1.644	16.52	30	<0.0001
	5: N2-1-1 vs. 7: N2-1-1	8.413	2.356 to 14.47	1.644	7.238	30	0.0013
	5: N2-1-1 vs. 7: Nd-1-1	6.667	0.6105 to 12.72	1.644	5.737	30	0.0206
	5: N2-1-1 vs. 7: Pb-2-2	5.149	−0.9075 to 11.21	1.644	4.431	30	0.1634
	5: Nd-1-1 vs. 5: Pb-2-2	−1.427	−7.484 to 4.630	1.644	1.228	30	0.9998
	5: Nd-1-1 vs. 7: Blank	−6.898	−12.95 to −0.8409	1.644	5.935	30	0.0145
	5: Nd-1-1 vs. 7: Pathogen	20.58	14.52 to 26.64	1.644	17.71	30	<0.0001
	5: Nd-1-1 vs. 7: N2-1-1	9.79	3.733 to 15.85	1.644	8.423	30	0.0001
	5: Nd-1-1 vs. 7: Nd-1-1	8.044	1.987 to 14.10	1.644	6.921	30	0.0024
	5: Nd-1-1 vs. 7: Pb-2-2	6.526	0.4692 to 12.58	1.644	5.615	30	0.0254
	5: Pb-2-2 vs. 7: Blank	−5.471	−11.53 to 0.5863	1.644	4.707	30	0.1099
	5: Pb-2-2 vs. 7: Pathogen	22.01	15.95 to 28.06	1.644	18.93	30	<0.0001
	5: Pb-2-2 vs. 7: N2-1-1	11.22	5.160 to 17.27	1.644	9.651	30	<0.0001
	5: Pb-2-2 vs. 7: Nd-1-1	9.471	3.414 to 15.53	1.644	8.149	30	0.0002
	5: Pb-2-2 vs. 7: Pb-2-2	7.953	1.896 to 14.01	1.644	6.843	30	0.0027
	7: Blank vs. 7: Pathogen	27.48	21.42 to 33.53	1.644	23.64	30	<0.0001
	7: Blank vs. 7: N2-1-1	16.69	10.63 to 22.74	1.644	14.36	30	<0.0001
	7: Blank vs. 7: Nd-1-1	14.94	8.885 to 21.00	1.644	12.86	30	<0.0001
	7: Blank vs. 7: Pb-2-2	13.42	7.367 to 19.48	1.644	11.55	30	<0.0001
*Phomopsis*	7: Pathogen vs. 7: N2-1-1	−10.79	−16.85 to −4.732	1.644	9.283	30	<0.0001
	7: Pathogen vs. 7: Nd-1-1	−12.53	−18.59 to −6.478	1.644	10.78	30	<0.0001
	7: Pathogen vs. 7: Pb-2-2	−14.05	−20.11 to −7.996	1.644	12.09	30	<0.0001
	7: N2-1-1 vs. 7: Nd-1-1	−1.745	−7.802 to 4.311	1.644	1.502	30	0.9986
	7: N2-1-1 vs. 7: Pb-2-2	−3.263	−9.320 to 2.793	1.644	2.808	30	0.7911
	7: Nd-1-1 vs. 7: Pb-2-2	−1.518	−7.575 to 4.539	1.644	1.306	30	0.9997
	3: Blank vs. 3: Pathogen	−0.676	−8.023 to 6.671	1.994	0.4795	30	>0.9999
	3: Blank vs. 3: N2-1-1	−5.384	−12.73 to 1.964	1.994	3.819	30	0.351
	3: Blank vs. 3: Nd-1-1	−6.187	−13.53 to 1.160	1.994	4.388	30	0.1732
	3: Blank vs. 3: Pb-2-2	−7.657	−15.00 to −0.3100	1.994	5.431	30	0.0347
	3: Blank vs. 5: Blank	−10.95	−18.30 to −3.607	1.994	7.77	30	0.0005
	3: Blank vs. 5: Pathogen	−7.078	−14.43 to 0.2698	1.994	5.02	30	0.068
	3: Blank vs. 5: N2-1-1	−14.56	−21.91 to −7.211	1.994	10.33	30	<0.0001
	3: Blank vs. 5: Nd-1-1	−16.16	−23.51 to −8.812	1.994	11.46	30	<0.0001
	3: Blank vs. 5: Pb-2-2	−14.97	−22.32 to −7.620	1.994	10.62	30	<0.0001
	3: Blank vs. 7: Blank	−21.52	−28.87 to −14.18	1.994	15.27	30	<0.0001
	3: Blank vs. 7: Pathogen	4.953	−2.395 to 12.30	1.994	3.513	30	0.479
	3: Blank vs. 7: N2-1-1	−6.334	−13.68 to 1.013	1.994	4.493	30	0.1499
	3: Blank vs. 7: Nd-1-1	−5.045	−12.39 to 2.303	1.994	3.578	30	0.4502
	3: Blank vs. 7: Pb-2-2	−6.04	−13.39 to 1.307	1.994	4.284	30	0.1992
	3: Pathogen vs. 3: N2-1-1	−4.708	−12.06 to 2.640	1.994	3.339	30	0.558
	3: Pathogen vs. 3: Nd-1-1	−5.511	−12.86 to 1.836	1.994	3.909	30	0.3171
	3: Pathogen vs. 3: Pb-2-2	−6.981	−14.33 to 0.3660	1.994	4.952	30	0.0757
	3: Pathogen vs. 5: Blank	−10.28	−17.63 to −2.931	1.994	7.29	30	0.0012
	3: Pathogen vs. 5: Pathogen	−6.402	−13.75 to 0.9458	1.994	4.541	30	0.14
	3: Pathogen vs. 5: N2-1-1	−13.88	−21.23 to −6.535	1.994	9.846	30	<0.0001
	3: Pathogen vs. 5: Nd-1-1	−15.48	−22.83 to −8.136	1.994	10.98	30	<0.0001
	3: Pathogen vs. 5: Pb-2-2	−14.29	−21.64 to −6.944	1.994	10.14	30	<0.0001
	3: Pathogen vs. 7: Blank	−20.85	−28.19 to −13.50	1.994	14.79	30	<0.0001
	3: Pathogen vs. 7: Pathogen	5.629	−1.719 to 12.98	1.994	3.992	30	0.2876
	3: Pathogen vs. 7: N2-1-1	−5.658	−13.01 to 1.689	1.994	4.013	30	0.2806
	3: Pathogen vs. 7: Nd-1-1	−4.369	−11.72 to 2.979	1.994	3.099	30	0.6683
	3: Pathogen vs. 7: Pb-2-2	−5.364	−12.71 to 1.983	1.994	3.805	30	0.3563
	3: N2-1-1 vs. 3: Nd-1-1	−0.8034	−8.151 to 6.544	1.994	0.5698	30	>0.9999
	3: N2-1-1 vs. 3: Pb-2-2	−2.274	−9.621 to 5.074	1.994	1.613	30	0.9971
	3: N2-1-1 vs. 5: Blank	−5.571	−12.92 to 1.777	1.994	3.951	30	0.3019
	3: N2-1-1 vs. 5: Pathogen	−1.694	−9.041 to 5.654	1.994	1.201	30	0.9999
	3: N2-1-1 vs. 5: N2-1-1	−9.174	−16.52 to −1.827	1.994	6.507	30	0.0051
	3: N2-1-1 vs. 5: Nd-1-1	−10.78	−18.12 to −3.428	1.994	7.643	30	0.0006
	3: N2-1-1 vs. 5: Pb-2-2	−9.584	−16.93 to −2.237	1.994	6.798	30	0.003
	3: N2-1-1 vs. 7: Blank	−16.14	−23.49 to −8.792	1.994	11.45	30	<0.0001
	3: N2-1-1 vs. 7: Pathogen	10.34	2.989 to 17.68	1.994	7.331	30	0.0011
	3: N2-1-1 vs. 7: N2-1-1	−0.9502	−8.298 to 6.397	1.994	0.674	30	>0.9999
	3: N2-1-1 vs. 7: Nd-1-1	0.3389	−7.008 to 7.686	1.994	0.2404	30	>0.9999
	3: N2-1-1 vs. 7: Pb-2-2	−0.6566	−8.004 to 6.691	1.994	0.4657	30	>0.9999
	3: Nd-1-1 vs. 3: Pb-2-2	−1.47	−8.818 to 5.877	1.994	1.043	30	>0.9999
	3: Nd-1-1 vs. 5: Blank	−4.767	−12.11 to 2.580	1.994	3.381	30	0.5386
	3: Nd-1-1 vs. 5: Pathogen	−0.8905	−8.238 to 6.457	1.994	0.6316	30	>0.9999
	3: Nd-1-1 vs. 5: N2-1-1	−8.371	−15.72 to −1.023	1.994	5.937	30	0.0144
	3: Nd-1-1 vs. 5: Nd-1-1	−9.972	−17.32 to −2.625	1.994	7.073	30	0.0018
	3: Nd-1-1 vs. 5: Pb-2-2	−8.781	−16.13 to −1.433	1.994	6.228	30	0.0086
	3: Nd-1-1 vs. 7: Blank	−15.34	−22.68 to −7.988	1.994	10.88	30	<0.0001
	3: Nd-1-1 vs. 7: Pathogen	11.14	3.792 to 18.49	1.994	7.901	30	0.0004
	3: Nd-1-1 vs. 7: N2-1-1	−0.1469	−7.494 to 7.201	1.994	0.1042	30	>0.9999
	3: Nd-1-1 vs. 7: Nd-1-1	1.142	−6.205 to 8.490	1.994	0.8102	30	>0.9999
	3: Nd-1-1 vs. 7: Pb-2-2	0.1467	−7.201 to 7.494	1.994	0.1041	30	>0.9999
	3: Pb-2-2 vs. 5: Blank	−3.297	−10.64 to 4.050	1.994	2.339	30	0.9313
	3: Pb-2-2 vs. 5: Pathogen	0.5798	−6.768 to 7.927	1.994	0.4113	30	>0.9999
	3: Pb-2-2 vs. 5: N2-1-1	−6.901	−14.25 to 0.4469	1.994	4.894	30	0.0828
	3: Pb-2-2 vs. 5: Nd-1-1	−8.502	−15.85 to −1.154	1.994	6.03	30	0.0122
	3: Pb-2-2 vs. 5: Pb-2-2	−7.31	−14.66 to 0.03698	1.994	5.185	30	0.0522
	3: Pb-2-2 vs. 7: Blank	−13.87	−21.21 to −6.518	1.994	9.834	30	<0.0001
	3: Pb-2-2 vs. 7: Pathogen	12.61	5.263 to 19.96	1.994	8.944	30	<0.0001
	3: Pb-2-2 vs. 7: N2-1-1	1.323	−6.024 to 8.671	1.994	0.9387	30	>0.9999
	3: Pb-2-2 vs. 7: Nd-1-1	2.613	−4.735 to 9.960	1.994	1.853	30	0.9893
	3: Pb-2-2 vs. 7: Pb-2-2	1.617	−5.730 to 8.964	1.994	1.147	30	>0.9999
	5: Blank vs. 5: Pathogen	3.877	−3.471 to 11.22	1.994	2.75	30	0.813
	5: Blank vs. 5: N2-1-1	−3.603	−10.95 to 3.744	1.994	2.556	30	0.8772
	5: Blank vs. 5: Nd-1-1	−5.205	−12.55 to 2.143	1.994	3.692	30	0.4019
	5: Blank vs. 5: Pb-2-2	−4.013	−11.36 to 3.334	1.994	2.847	30	0.776
	5: Blank vs. 7: Blank	−10.57	−17.92 to −3.221	1.994	7.496	30	0.0008
	5: Blank vs. 7: Pathogen	15.91	8.560 to 23.25	1.994	11.28	30	<0.0001
	5: Blank vs. 7: N2-1-1	4.621	−2.727 to 11.97	1.994	3.277	30	0.5866
	5: Blank vs. 7: Nd-1-1	5.91	−1.438 to 13.26	1.994	4.192	30	0.2248
	5: Blank vs. 7: Pb-2-2	4.914	−2.433 to 12.26	1.994	3.485	30	0.4913
	5: Pathogen vs. 5: N2-1-1	−7.48	−14.83 to −0.1330	1.994	5.306	30	0.0428
	5: Pathogen vs. 5: Nd-1-1	−9.082	−16.43 to −1.734	1.994	6.441	30	0.0058
	5: Pathogen vs. 5: Pb-2-2	−7.89	−15.24 to −0.5429	1.994	5.596	30	0.0262
	5: Pathogen vs. 7: Blank	−14.45	−21.79 to −7.098	1.994	10.25	30	<0.0001
	5: Pathogen vs. 7: Pathogen	12.03	4.683 to 19.38	1.994	8.533	30	0.0001
	5: Pathogen vs. 7: N2-1-1	0.7436	−6.604 to 8.091	1.994	0.5274	30	>0.9999
	5: Pathogen vs. 7: Nd-1-1	2.033	−5.315 to 9.380	1.994	1.442	30	0.9991
	5: Pathogen vs. 7: Pb-2-2	1.037	−6.310 to 8.385	1.994	0.7357	30	>0.9999
	5: N2-1-1 vs. 5: Nd-1-1	−1.601	−8.949 to 5.746	1.994	1.136	30	>0.9999
	5: N2-1-1 vs. 5: Pb-2-2	−0.4099	−7.757 to 6.938	1.994	0.2907	30	>0.9999
	5: N2-1-1 vs. 7: Blank	−6.965	−14.31 to 0.3827	1.994	4.94	30	0.0771
	5: N2-1-1 vs. 7: Pathogen	19.51	12.16 to 26.86	1.994	13.84	30	<0.0001
	5: N2-1-1 vs. 7: N2-1-1	8.224	0.8766 to 15.57	1.994	5.833	30	0.0174
	5: N2-1-1 vs. 7: Nd-1-1	9.513	2.166 to 16.86	1.994	6.748	30	0.0033
	5: N2-1-1 vs. 7: Pb-2-2	8.518	1.170 to 15.87	1.994	6.041	30	0.012
	5: Nd-1-1 vs. 5: Pb-2-2	1.191	−6.156 to 8.539	1.994	0.845	30	>0.9999
	5: Nd-1-1 vs. 7: Blank	−5.363	−12.71 to 1.984	1.994	3.804	30	0.3566
	5: Nd-1-1 vs. 7: Pathogen	21.11	13.76 to 28.46	1.994	14.97	30	<0.0001
	5: Nd-1-1 vs. 7: N2-1-1	9.825	2.478 to 17.17	1.994	6.969	30	0.0022
	5: Nd-1-1 vs. 7: Nd-1-1	11.11	3.767 to 18.46	1.994	7.883	30	0.0004
	5: Nd-1-1 vs. 7: Pb-2-2	10.12	2.771 to 17.47	1.994	7.177	30	0.0015
	5: Pb-2-2 vs. 7: Blank	−6.555	−13.90 to 0.7926	1.994	4.649	30	0.1197
	5: Pb-2-2 vs. 7: Pathogen	19.92	12.57 to 27.27	1.994	14.13	30	<0.0001
	5: Pb-2-2 vs. 7: N2-1-1	8.634	1.286 to 15.98	1.994	6.124	30	0.0103
	5: Pb-2-2 vs. 7: Nd-1-1	9.923	2.576 to 17.27	1.994	7.038	30	0.0019
	5: Pb-2-2 vs. 7: Pb-2-2	8.928	1.580 to 16.27	1.994	6.332	30	0.0071
	7: Blank vs. 7: Pathogen	26.48	19.13 to 33.82	1.994	18.78	30	<0.0001
	7: Blank vs. 7: N2-1-1	15.19	7.841 to 22.54	1.994	10.77	30	<0.0001
	7: Blank vs. 7: Nd-1-1	16.48	9.130 to 23.83	1.994	11.69	30	<0.0001
	7: Blank vs. 7: Pb-2-2	15.48	8.135 to 22.83	1.994	10.98	30	<0.0001
	7: Pathogen vs. 7: N2-1-1	−11.29	−18.63 to −3.939	1.994	8.005	30	0.0003
	7: Pathogen vs. 7: Nd-1-1	−9.998	−17.34 to −2.650	1.994	7.091	30	0.0017
	7: Pathogen vs. 7: Pb-2-2	−10.99	−18.34 to −3.646	1.994	7.797	30	0.0004
	7: N2-1-1 vs. 7: Nd-1-1	1.289	−6.058 to 8.637	1.994	0.9144	30	>0.9999
	7: N2-1-1 vs. 7: Pb-2-2	0.2936	−7.054 to 7.641	1.994	0.2082	30	>0.9999
	7: Nd-1-1 vs. 7: Pb-2-2	−0.9956	−8.343 to 6.352	1.994	0.7061	30	>0.9999

Note: PAL, phenylalanine ammonia-lyase. The comparisons are denoted as “time point:treatment”, where time points “3”, “5”, and “7” indicate days post-inoculation; “Pathogen” refers to the original pathogen isolate; “N2-1-1”, “Nd-1-1”, and “Pb-2-2” are distinct microbial strains/isolates; “Blank” represents the non-inoculated control. For each pairwise comparison, the mean difference, 95% confidence interval (CI) of the difference, standard error of the difference (SE), studentized range statistic (q), degrees of freedom (df), and adjusted *p*-value (Tukey’s method) are presented. Due to a balanced design and homogeneity of variance, the SE of the difference (2.161) and the degrees of freedom (30) are constant across all comparisons. The critical significance level was set at α = 0.05, and adjusted *p*-values were computed to control the family-wise error rate.
